# Imidazolium Ionic Liquids as Designer Solvents Confined in Silica Nanopores

**DOI:** 10.3390/gels8060388

**Published:** 2022-06-19

**Authors:** Ana-Maria Putz, Adél Len, László Trif, Zsolt Endre Horváth, László Almásy

**Affiliations:** 1 “Coriolan Drăgulescu” Institute of Chemistry, Bv. Mihai Viteazul, No. 24, 300223 Timisoara, Romania; 2 Institute for Energy Security and Environmental Safety, Centre for Energy Research, Konkoly-Thege 29-33, 1121 Budapest, Hungary; len.adel@ek-cer.hu; 3 Faculty of Engineering and Information Technology, University of Pécs, Boszorkány u. 2, 7624 Pécs, Hungary; 4 Institute of Materials and Environmental Chemistry, Research Centre for Natural Sciences, Magyar Tudósok Körútja 2, 1117 Budapest, Hungary; trif.laszlo@ttk.hu; 5 Institute for Technical Physics and Material Science, Centre for Energy Research, Konkoly-Thege út 29-33, 1121 Budapest, Hungary; horvath.zsolt.endre@ek-cer.mta.hu

**Keywords:** ionic liquid, 1-butyl-3-methylimidazolium tetrafluoroborate, 1-butyl-3-methylimidazolium chloride, mesoporous silica, small-angle neutron scattering

## Abstract

Composite silica xerogels were prepared via acid catalysed sol–gel route using tetraethoxysilan (TEOS) as silica precursor, and 1-butyl-3-methylimidazolium tetrafluoroborate [BMIM][BF_4_] or 1-butyl-3-methylimidazolium chloride [BMIM][Cl] ionic liquids, used simultaneously as co-solvents, catalysts and pore templates, at various IL-to-silica ratios. Morphology of the xerogels prepared using the different IL templating agents were investigated using scanning electron microscopy (SEM), nitrogen sorption and small angle neutron scattering (SANS). The thermal behavior of the composites was analyzed by thermal gravimetry, whereas the compositions were checked by infrared spectroscopy and EDX. The differences in the morphology and thermal behavior of the composites due to the different IL additives were revealed.

## 1. Introduction

Ionic liquids (IL) are well known as designer solvents for the synthesis of new compounds and functional materials. Thanks to various possibilities in tailoring their molecular structure and fine tuning their solvent properties [1], they are recognized as a third group of solvents, following water and organic solvents. In spite of this, they are considerably affected by Lewis acidity or basicity of their cations or anions (Coulombic interactions), by the directionality of interactions between cations and anions, and by the van der Waals interactions between ions [2], they are typically characterized by unique solvent properties. The selection of different anions or usage of different alkyl groups in the heterocyclic rings of the cations, permits the fine-tuning of physicochemical properties of ILs, such as viscosity, solvation, catalytic activity, hydrophobicity and melting point [3]. By choosing various anions, their miscibility with water and with other solvents, and their ability to form hydrogen bonds can be varied. By choosing different cations, their hydrophobicity can be also varied, causing changes in the properties of the composite materials prepared with ILs [4,5]. ILs used as pore templates in polymer matrices permit tailoring of the gel pore size [6]. Using ILs the stability of polymeric gels can be improved. With increased aging time the pore volume diminishes, whereas short aging produces stronger gel network; these processes are controlled by the solvent evaporation speed [7]. It has been shown that the addition of small amount of IL to aqueous mixtures of silica precursors slows down the polycondensation reaction and produces primary silica particles of larger sizes [8]. Composite materials based on silica and prepared by sol–gel route typically have heterogeneous structure on nanometer length scales, which can be studied by small-angle neutron and X-ray scattering (SANS and SAXS) methods. Small-angle scattering helps elucidating the morphology of silica–IL composites, showing the effect of the IL as catalyst and as pore template [9].

Ionic liquids are highly promising additives for resource-efficient (low-temperature/low-pressure) synthesis of functional materials, ensure higher purity and yield less side-reactions and side-products. The production of new compounds with fascinating compositions and structures and outstanding properties is possible via synthesis near room temperature, thus, at low activation energy and with kinetic synthesis control [1].

One of the most important applications of ILs as solvents is undoubtedly their usage in the energy storage field, because of an increasing demand for clean and sustainable energy [2]. Tuning the hydrophilicity or hydrophobicity, the viscosity and the coordination properties, especially those implied by the anion, makes them suitable for hydrogen storage [10]. When combined with basic anions [11] or cations (e.g., imidazolium) ILs exert a strong effect on the hydrogen yield, showing that IL material blends are competitive with conventional hydrogen storage materials with experimental efficiencies of at least 6.5 wt% H_2_ [12]. ILs have also been described as designer solvents [13]; their properties can be adjusted to meet the expectations of the desired process.

Previous studies have shown that certain short alkyl chain ILs can self-aggregate in aqueous solvents [14,15]. Alkyl tails of the IL cations interact through van der Waals attractions, resulting in micelle formation. The size of micelles increases with IL concentration [15]. In the sol–gel reaction mixture, the condensing silica oligomers adsorb onto the surface of the micelles, reduce the inter-micellar repulsion resulting in aggregation of the micelles with a silica layer between them, thus forming initial silica nuclei. From this point forward, the growth occurs in a cooperative manner, with condensing silica filling the gaps between further aggregating micelles [8].

ILs with long alkyl tails, mixed with silica precursors behave similarly to the more common cationic surfactants, and in appropriate conditions form long parallel micelles with hexagonal ordering [16]. Such micellar solution, mixed with silica precursors, can be used for formation of MCM-41-like silica composites, which can be converted in ordered mesoporous silica after removing the ionic liquid by calcination or solvent extraction [17,18,19,20,21,22].

On the other hand, short-chain ILs do not form proper micelles [14], but do agglomerate or self-associate, providing confinement and promoting the agglomeration of the condensed silica species [9]. In our previous study, new composite materials using a short alkyl chain ionic liquid, N-butyl-3-methylpyridinium tetrafluoroborate as templating agent, and tetramethoxysilane (TMOS) and methyl-trimethoxysilane (MTMS) as silica precursors have been prepared [23]. Varying the amount of the IL over a broad range, the slowing of the condensation reaction and increase in the silica particle size could be observed [23].

The aim of the present study was to explore the influence of the IL type on the resulting morphology of the composite xerogels. The materials have been prepared using short alkyl chain Ils with the same cation, 1-butyl-3-methylimidazolium ([BMIM]), and different anions. Subsequently, the IL template has been removed by extraction with ethanol. The molar ratio IL/Si was varied as follows: 0.1, 0.3 and 0.5. The xerogels were prepared via acid catalyzed sol–gel route using tetraethoxysilane (TEOS) as silica precursor, and 1-butyl-3-methylimidazolium tetrafluoroborate ([BMIM][BF_4_] for one series or 1-butyl-3-methylimidazolium chloride ([BMIM][Cl]), for the second series, acting as co-solvents, catalysts and pore templates, varying only the amount of the IL template and keeping a constant ratio of the others reactants. The ILs anions hydrophobicity is increasing from chlorine to tetrafluoroborate; therefore, the [BMIM][BF_4_]-containing samples were expected to be more hydrophobic compared to the [BMIM][Cl]-containing samples. Morphology of the prepared materials were investigated using different structure-sensitive experimental methods.

## 2. Results and Discussion

### 2.1. FT-IR Analysis of the Neat Ionic Liquids, Xerogels and Xerogels after Solvent Extraction

**Figure 1:**
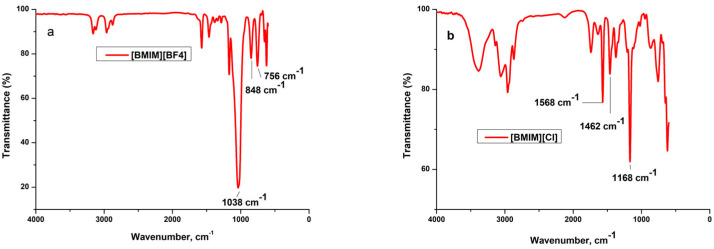
The FT-IR spectra for the neat ionic liquids (**a**) [BMIM][BF_4_] and (**b**) [BMIM][Cl].

**Figure 2:**
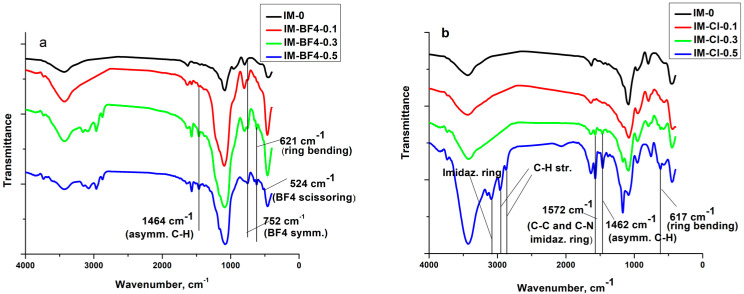
FT-IR spectra for xerogel samples (**a**) IM-BF4-x and (**b**) IM-Cl-x series.

**Figure 3:**
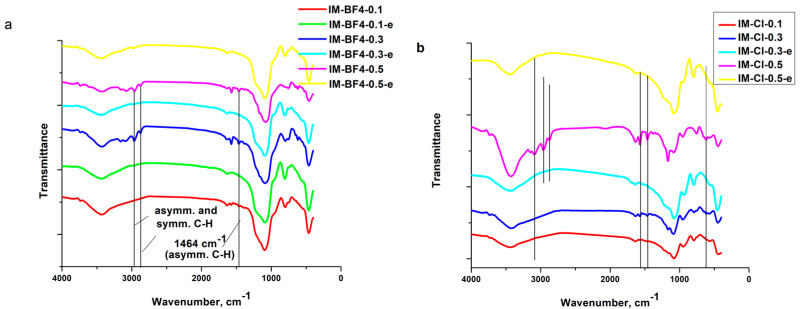
FT-IR spectra for the xerogels and the extracted samples. (**a**) IM-BF4-x series and (**b**) IM-Cl-x series.

The FT-IR spectra of neat ILs, the xerogel samples and the materials resulted after the IL extraction (named “e”), are presented in Figure 1, Figure 2 and Figure 3 and the band assignments are given in Table 1 and Table 2.

#### 2.1.1. Vibration Bands of Ils and Ils in Silica Matrix

The C=C stretching from the imidazolium ring bands around 1635 cm^−1^ [24] and can be well distinguished in the neat [BMIM][Cl].

The imidazolium ring CH_3_–HCH asymmetric stretching vibration at 3150 cm^−1^ and the imidazolium (N)CH_2_–HCH symmetric stretching vibration at 3090 cm^−1^ [25]; as well as aliphatic methylene group vibrations at 2962 and 2873 cm^−1^ [24,26], are present in the neat ILs and in the composites with high IL concentration. After the ILs extraction these bands disappear. The in-plane C–C and C–N stretching vibrations of the imidazolium ring around 1570 cm^−1^ [27,28] are visible in the neat ILs and in the xerogels with high IL content. In the extracted samples of the BF_4_ series this band is still visible, showing proving that [BMIM][BF_4_] was not fully removed in these samples.

The ring CH_3_-CN stretching vibration [25], C–C vibration [29,30] and due to asymmetric C–H bond stretching vibration [31] of the alkyl chain bands are around 1463 cm^−1^ (from literature, found at 1476 cm^−1^) [27]. The band is well visible in the neat ILs and in the xerogels with increased ILs concentration, disappearing in the samples where the ILs has been extracted.

The C–C vibration [29] and asymmetric C–H vibration [31] bands are around 1375 cm^−1^ (present in [BMIM][Cl] and in the xerogel with the highest concentration containing this IL) and from 1382 cm^−1^ (present only in [BMIM][BF_4_]).

The symmetrical C–H vibration of imidazolium ring, assigned to the imidazolium group in the neat ILs, observable only in [BMIM][BF_4_] at 1285 cm^−1^ [27].

The in-plane C–H deformation vibration of imidazolium ring [27], around 1167–1170 cm^−1^, is superposed with the silica matrix bands and can be seen separately only in the xerogels with high [BMIM][Cl] concentration. In the IM-0.5-Cl sample this band is even stronger than the characteristic band of the silica matrix at 1084 cm^−1^ [32].

One of the characteristics bands of BF_4_ group at 1135 cm^−1^ [27] is superposed with the broad band of the silica matrix at 1084 cm^−1^ and could be distinguished only in the neat IL at 1038 cm^−1^.

At 848 cm^−1^ is observable the characteristic band due to C–N stretching vibration [24] and B–F symmetric stretching vibration [25] only in the neat [BMIM][BF_4_].

Between 758–808 cm^−1^ are observable in all the samples, the characteristic band due to C–C stretching vibration [32].

Only in the neat [BMIM][BF_4_] IL and in the xerogels synthetized with it, the characteristic band due to BF_4_ symmetric stretching vibration [25,27] is observable around 750 cm^−1^. In the xerogel with the highest IL concentration, the band at 525 cm^−1^ is from the BF_4_ scissoring vibrations [25]. The characteristic band for ring HC–CH asymmetric bending [25] vibration around 695 cm^−1^ are observable only in the neat ILs. The ones for ring bending [25] vibration around 650 cm^−1^ and 620 cm^−1^ are observable not only in the neat ILs, but also in the xerogels containing ILs in higher concentrations (the band around 650 cm^−1^_,_ only for the xerogels containing [BMIM][BF_4_]).

#### 2.1.2. Vibration Bands of the Silica Matrix and Adsorbed Water

The superposing of the O–H stretching bands of hydrogen-bonded water molecules (H–O–H … H) and Si–O–H stretching of surface silanols hydrogen-bonded to molecular water (Si–O–H … H_2_O) [33] around 3733 cm^−1^ and in the 3421–3436 cm^−1^ range are stronger as the IL concentration increase, in all composites, indicating the higher water content in the ionogels.

The Si–OH bond stretching [32] around 950 cm^−1^, is better observed in samples prepared with [BMIM][Cl]. The Si–O bond rocking [32] vibration around 450 cm^−1^, is observable in all obtained samples.

**Table 1:** FT-IR bands assignments of the silica xerogel IM-0, the composite xerogels and the extracted samples of the IM-BF4 series.

Band Assignment	IM-0	IM-BF4-0.1	IM-BF4-0.3	IM-BF4-0.5	IM-BF4-0.1-e	IM-BF4-0.3-e	IM-BF4-0.5-e	[BMIM][BF_4_] Neat
O–H stretching [33]	-	-	3734	3735		3734	3736	-
	3435	3431	3429	3437	3432	3437	3446	-
Imidazolium ring CH_3_–HCH asymmetric stretching and (N)CH_2_–HCH symmetric stretching [25]	-	-	3159, 3087	3151, 3076	-	-	3175	3161
Aliphatic asymmetric and symmetric C–H stretching [24,26]	-	-	2964, 2875	2964, 2873	-	-	-	2964
OH bending of water [32]	1628	1631	1628	1630	1630	1634	1635	-
In-plane C–C and C–N stretching of imidazolium ring [27,28]	-	1572	1572	1570	1569	1568	1568	1572
Ring CH_3_–CN stretching vibration [25] C–C vibration [29,30] and asymmetric C–H vibration [31]	-	-	1464	1464	-	-	-	1465
CH_2_ scissoring and bending of unreacted TEOS [32]	1446, 1394	-	-	-	-	-	-	-
C–C vibration [29,30] and asymmetric C–H vibration [31]	-	-	-	-	-	-	-	1382
Symmetric C–H vibration of imidazolium ring [27]	-	-	-	-	-	-	-	1285
In-plane C–H deformation vibration of imidazolium ring [27]	-	-	-	-	-	-	-	1170
Asymmetric Si–O–Si stetching [32]	1086	1093	1088	1080	1094	1092	1093	
BF_4_ asymmetric stretching	-	-	-	-	-	-	-	1038
Si–OH bond stretching [32]	958	-	-	-	-	-	-	
C–N stretching [24] and BF_4_ symmetric stretching [25]	-	-	-	-	-	-	-	848
C–C stretching vibration [32]	795	800	796	802	805	806	808	
BF_4_ symmetric stretching [25,27]	-	750	752	754	-	-	-	756
Ring HC–CH asymmetric bending [25]	-	-	-	-	-	-	-	697
Ring bending [25]	-	-	650	652	-	-	-	651
	-	-	621	621	-	-	-	622
BF_4_ scissoring [25]	-	-	-	525	-	-	-	-
Si–O bond rocking [32]	447	465	463	461	464	463	461	-

**Table 2:** FT-IR bands assignments of the xerogels and extracted samples for IM-Cl series.

Band Assignment	IM-0	IM-Cl-0.1	IM-Cl-0.3	IM-Cl-0.5	IM-Cl-0.3-e	IM-Cl-0.5-e	[BMIM][Cl]
O–H stretching bands [33]			3734	3735	-	-	-
	3435	3438	3421	3429	3437	3437	3387
Imidazolium ring CH_3_–HCH asymmetric stretching and (N)CH_2_ HCH symmetric stretching [25]	-	-	-	3149, 3091	-	-	3141, 3060
Aliphatic asymmetric and symmetric C–H stretching vibration [24,25]	-	-	-	2962, 2874	-	-	2959, 2870
OH bending molecular water [32]	1628	1635	1641	1635	1638	1627	1637
In-plane C–C and C–N stretching vibrations of the imidazolium ring [27,28]	-	-	1572	1572	-	-	1568
Ring CH_3_–CN stretching [25], C–C vibration [31] and asymmetric C–H vibration [31]	-	-	1466	1462	-	-	1462
CH_2_ scissoring and bending of unreacted TEOS [32]	1446, 1394	-	-	-	-	-	-
C–C vibration [31] and asymmetric C–H vibration [31]	-	-		1383	-	-	1375
In-plane C–H deformation vibration of imidazolium ring [27]	-	-	1167	1169	-	-	1168
Asymmetric Si–O–Si stetching [32]	1086	1080	1084	1084	1083	1081	
Si–OH bond stretching [32]	958	949	953	955	936	951	948
C–N stretching [24]	-	-	-	-	-	-	840
C–C stretching [32]	795	793	793	756	794	794	757
Ring HC–CH asymmetric bending [25]	-	-	-	-	-	-	692
Ring bending [25]	-	-	-	-	-	-	650
Ring bending [25] Si–O bond rocking [32]	-	-	617	617	-	-	620
	447	444	447	447	456	453	-

### 2.2. Nitrogen Porosimetry

The xerogel composite samples containing the ionic liquids were essentially non-porous or had closed pores, resulting in non-measurable porosity by nitrogen sorption. The xerogels with after IL extraction were further analysed and their porosity evaluated.

**Figure 4:**
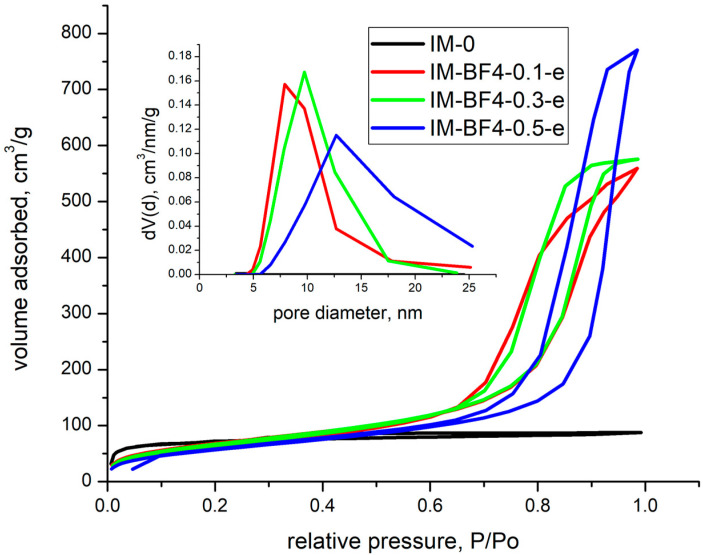
Nitrogen sorption isotherms and the pore size distribution for the samples synthetized with [BMIM][BF_4_] after extraction of the IL.

The nitrogen sorption isotherms and the pore size distribution for the samples synthetized with [BMIM][BF_4_] are shown in Figure 4. In the case of samples synthetized with [BMIM][Cl], the results indicated that all pores were closed, and the surface area was close to 0.

The textural parameters are collected in Table 3. In case of blank sample, IM-0-e, the isotherms are type IVa with H4 hysteresis type, meaning that the sample presents also microporosity beside mesoporosity. When [BMIM][BF_4_] was added, a type IVa isotherm with a H2b hysteresis has been obtained in all cases. This type of hysteresis is specific for samples with broad pore size distribution. By analysing the data, it was observed that both, the surface area and rugosity are decreasing with the increase in [BMIM][BF_4_] concentration. In case of blank sample, IM-0-e, microporosity constitutes 70 % (193 m^2^/g) from the total specific surface area of 273 m^2^/g. In the samples synthetised with [BMIM][BF_4_], mesoporosity only has been observed and the specific surface area decreased with increasing the IL amount). At the same time, the total pore volume was four times higher than that in silica for the intermediate [BMIM][BF_4_] concentrations 0.1 and 0.3.

The pore sizes were calculated in the 0.35–1 P/P_0_ domain and their value increase with [BMIM][BF_4_] concentration, from 2.2 nm (for the blank sample) to 10–14.5 nm (for the samples synthetised with IL) demonstrating the porogen effect of the IL, even though the IL was not fully removed by extraction.

Additionally, in previous studies from literature, the solid state NMR spectra (^29^Si, ^1^H and ^13^C) showed that some residual [BMIM][BF_4_] remained attached to the aerogel network after the attempt to remove it via thermal treatment, thermal analysis demonstrating that around of 6% still remained entrapped into the silica matrix [34,35].

### 2.3. Thermal Analysis

**Figure 5:**
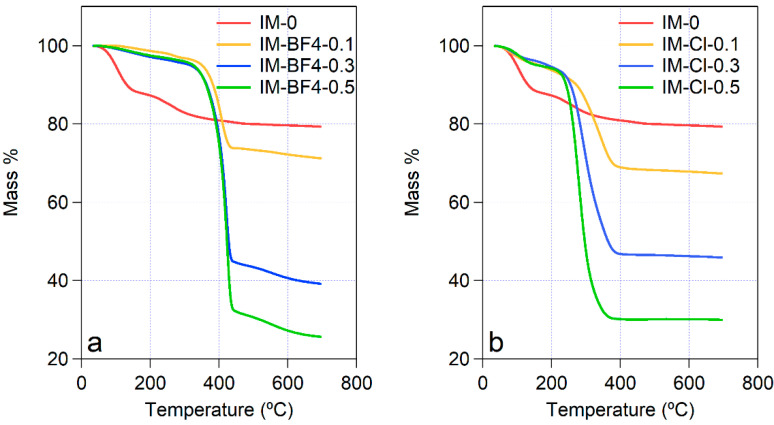
Thermogravimetric curves for (**a**) IM-BF4 series and (**b**) IM-Cl series of ionogels.

Thermal gravimetric curves are shown in Figure 5. The first weight loss step below 150 °C shows the evaporation of the water. The water content in the pure silica material IM-0 is much higher than in the more hydrophobic silica—IL composites. The water evaporation is much slower in the composites, and it continues until approximately 250–350 °C, for the different samples. Above 250 °C, a different behavior is observed between the samples of the two series. For all three samples in the IM-BF4 series, a sharp onset of a large mass loss is seen at 300 °C, followed by a second smaller mass loss starting at 500 °C. For the IM-Cl series, only one mass loss process is observed, which is broader than that in the IM-BF4 series, and starts at lowest temperature 230 °C for IM-Cl-0.1, and at higher temperature of 330 °C for IM-Cl-0.5. The organic groups of the Ils degrade and burn out in this step, furthermore no weight change occurs above 400 °C.

Comparing the observed behaviors, it can be said that the BF_4_-containing samples contain less water, that evaporate below 150 °C, and they are stable until higher temperature than the other series. Further on, the degradation and elimination of the BF_4_ group is responsible for the second mass loss region between 500 and 650 °C.

Concerning the thermal stability of the composite xerogels, by increasing the content of the more hydrophilic [BMIM][Cl], the thermal degradation onset temperature is appreciably lowering from about 280 °C to 240 °C. Conversely, in the [BMIM][BF_4_]-containing composites the mass loss due to the burning of the organic cation starts at about 240 °C in all three samples.

### 2.4. SANS Analysis

**Figure 6:**
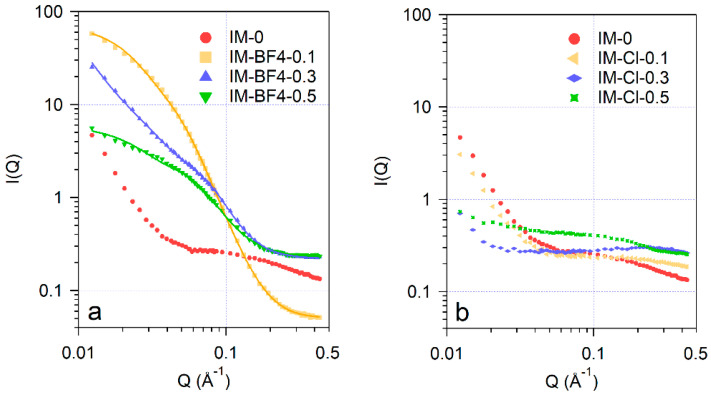
SANS scattering data of the xerogel composites: (**a**)—samples IM-BF4 and (**b**)—series IM-Cl. Solid lines are approximations by fitting the Beaucage model.

Small-angle neutron scattering curves of the nanocomposites are shown in Figure 6. The scattering data show the characteristic features of dry xerogels, which usually display a heterogeneous structure in the size range 1–50 nm, resulting from the non-uniform arrangement of the silica phase and the interparticle voids [36,37,38]. The shapes of the scattering curves for the two series of IL-silica composites are markedly different: samples containing [BMIM][BF_4_] show a high intensity plateau in the momentum transfer (*Q*) range 0.02–0.1 Å^−1^ indicating strongly heterogeneous structure with characteristic dimensions of 5–10 nm. The scattering intensities for the blank xerogel and samples prepared with [BMIM][Cl] in this *Q* range are by 1–2 orders of magnitude weaker, and only at the lowest *Q* the upturn of the intensity curve is seen, corresponding aggregates with size larger than 30 nm. The measured data have been analyzed by the empirical model function proposed by Beaucage [39], which provides the domain size, characterized by radius of gyration *Rg*, and the power law exponent *P* that characterizes the interface roughness between the constituent phases or particles:

$$I\left(Q\right)≅A\;\exp\left(-\frac{Q^{2}R_{g}{}^{2}}{3}\right)+B\;{\left\{\frac{{\left[\mathrm{erf}\left(\frac{QR_{g}}{\sqrt{6}}\right)\right]}^{3}}{Q}\right\}}^{P}+Bg$$

Parameter values obtained by least squares fitting are collected in Table 4. The power law exponents for the two lower IL content materials are close to −4, indicating sharp interfaces according to Porod law, between the silica and the IL or water phases, whereas in the highest IL content sample IM-BF4-0.5 this value is reduced to −3.1, corresponding to a rougher interface. The similar smooth character of the interface in dry [BMIM][BF_4_]–silica composite aerogels was also found by small-angle X-ray scattering by Wu and Lin [38]. The strong difference in the nanostruture of the composite gels prepared by using the two ILs can be understood considering the self-associtation features of the various ILs in their aqueous solutions. The hydrophilic Cl^−^ anion in [BMIM][Cl] favours a homogeneous distribution of the cation and anion in their aqueous solutions [40], whereas the less hydrophilic, and moderately hydrophobic BF_4_^−^ anion [41] causes a strongly heterogeneous structure of water—[BMIM][BF_4_] mixtures, most pronounced around their equivolumetric compositions [14,42,43]. This type of microheterogeneity is observable by small-angle neutron scattering, and is characterized by domain sizes around 1–2 nm at room temperature. The heterogeneity in aqueous IL solutions is strongly temperature dependent [43], and can also be affected by the presence of cosolvents, including TEOS and its hydrolysis products. Thus, addition of TEOS to solutions of tri-block copolymers results in change of the micelle shape from spherical to elongated [44]. In the sol–gel synthesis mixtures the heterogeneous nature of the mixed solvent leads to different distribution of the silica precursors in the water-rich and IL-rich domains, causing therefore different dynamics of the reactions and resulting, in turn, in different nanostructures of the composites made with the two kinds ILs.

### 2.5. Scanning Electron Microscopy and EDS Analysis

**Figure 7:**
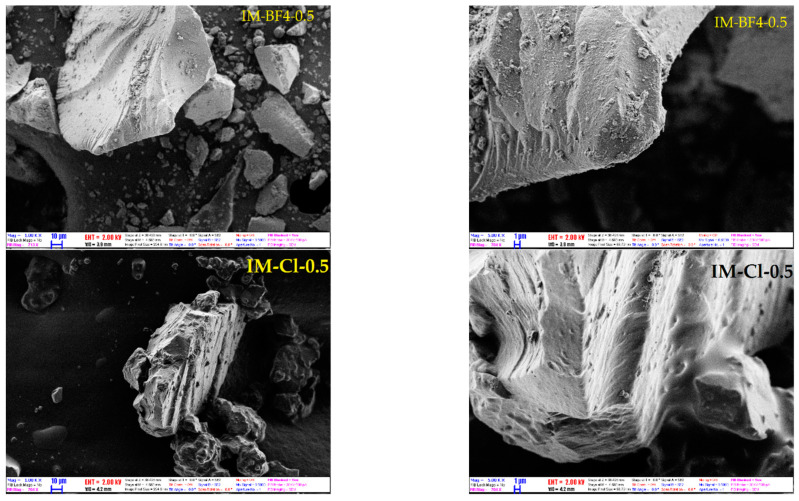
Characteristic SEM images of the hybrid silica materials IM-BF4-0.5 and IM-Cl-0.5, taken with magnifications 1000× (left) and 5000× (right).

Morphology of all prepared materials has been characterized by scanning electron microscopy. Images of samples from both series with the highest IL content are shown in Figure 7. Materials appeared as solid non-porous monolith particles with smooth surfaces with sizes mainly between 10 and 100 µm. No discernible difference could be seen between the [BMIM][BF_4_] and [BMIM][Cl] containing particles. EDS analysis confirmed the expected composition of the materials, with F and Cl atom content gradually increasing in the samples prepared with increasing IL concentration. Variations in atomic compositions compared to the reactant ratios used in synthesis were below 20 %. Analysis of the sample morphology in the micrometer size ranges therefore show little or no difference between the materials prepared using the two ionic liquid co-solvents. SEM images of all samples, Figures S1–S7, and EDS data, Figures S8 and S9, are placed in the Supplementary Materials.

## 3. Conclusions

Based on their unique characteristics with a high flexibility in varying the cations and the anions, the ILs have been generally named as designer solvents. In the present study, Ils with imidazolium cation and different anions, tetrafluoroborate chloride, were used for preparation of composite xerogels. The FT-IR and EDX analyses proved the entrapment of the two Ils into the silica matrix, the majority of the vibration bands being stronger with the increase in ILs concentration. The xerogel synthetised without IL present mesoporosity and microporosity as well. Different characteristic dimensions of the mesoscopic structure have been obtained for the two types of Ils, and attributed to the different character of structuring in the aqueous solutions of the two IL, which remains also in the mixtures with the silica precursor. The homogeneous [BMIM][Cl] solution led to formation of a more homogeneous composite xerogel, whereas from the heterogeneous [BMIM][BF_4_] solution rather heterogeneous nanocomposite had formed. The present results contribute to the understanding of phase separation in the mixtures of the gel precursors, and can serve for tailored design of templated xerogel composites.

## 4. Materials and Methods

### 4.1. Sample Preparation

All reagents were used as received: 1-butyl-3-methylimidazolium tetrafluoroborate ([BMIM][BF_4_] for synthesis 98%, Merck, Darmstadt, Germany); 1-butyl-3-methylimidazolium chloride ([BMIM][Cl] for synthesis 98%, Merck), tetraethoxysilane (TEOS, 99%, Sigma-Aldrich, Darmstadt, Germany); distilled water; and hydrochloric acid (HCl, 37%, S.C. Silal Trading SRL, Bucharest, Romania). Silica xerogels were prepared by mixing (for one sample): 0.0183 moles (3.81 g) TEOS, 0.145 moles distilled water (2.525 g H_2_O) and 0.0515 moles (0.93 g) acidic water (adjusted to pH = 1.5 by addition of HCl). Both the simple water and the acidic water were added drop wise for 5 min. The reaction mixture was mechanically vigorously stirred (30 min) in a round bottom flask until a homogenous sol was obtained. Next, the ionic liquid solutions in water were prepared by dissolving different quantities (Table 5) of [BMIM][BF_4_] and [BMIM][Cl] in 6.25 mL H_2_O. These IL/water mixtures were added into solution of the silica precursor in various IL/Si molar ratios (Table 5) under continuous stirring for 30 min. The obtained sols were left to gel at room temperature. The [BMIM][BF_4_], containing samples were gelled from 3 h up to the next day, therefore in less than 24 h; the blank sample and the [BMIM][Cl] containing samples, gelled in four days. The xerogel samples were obtained after drying (12 h) at 100 °C (for the IM-Cl-0.5 sample another 6 h of drying were necessary). For comparison purposes, blank sample was prepared using the same procedure, but without ionic liquid (sample IM-0); instead, an equivalent quantity of water was added (6.25 mL H_2_O).

The imidazolium ionic liquids were extracted from the xerogels samples by using the method of extraction with a solvent. For 1 g of xerogel material, around 25 mL ethanol was added. The extraction was realized with 5 h of stirring, and then the solutions were left overnight in ethanol. In the next day, the operation was repeated with a new ethanol washing solution, therefore, another 5 h of stirring and then, left overnight for complete filtration on the filter paper and drying at room temperature. In the third day, the samples were dried at 40 °C for one hour and at 100 °C, for 2 h. The efficiency of the extraction method was checked by IR analysis, taken before and after the extraction.

### 4.2. Characterization Methods

FT-IR spectra were recorded in KBr pellets using a JASCO FT/IR-4200 apparatus. Samples for transmittance measurements were prepared typically by mixing 3 mg of sample with 600 mg of KCl. The powdered samples were pelletized by applying 7.5 tons for several minutes under vacuum of several mm Hg.

SANS measurements were performed on the *Yellow Submarine* instrument at BNC in Budapest (Hungary) [45]. Collimation distance of 5 m and circular beam apertures of 25 and 7 mm in diameter defined the incoming beam divergence, whereas the sample for detector distances of 1.2 and 5.5 m and mean neutron wavelength of 4.1 Å defined the momentum transfer range of 0.015–0.42 Å^−1^. The scattered neutrons were detected by a two-dimensional position-sensitive BF_3_ gas detector (64 × 64 cells of 1 cm × 1 cm area). The raw data were corrected for sample transmissions and scattering of the empty cell, and converted to absolute units by comparison with the incoherent scattering of a 1 mm thick water sample. The powdered samples were filled into Hellma quartz cells of 2 mm flight path, and the measurements were conducted at room temperature. Nitrogen sorption was measured at 77K with a QuantaChrome Nova 2000-e instrument (Boynton Beach, Florida, United States). Before measurements, each sample was outgassed at room temperature for 6 h. The NovaWin software was used to evaluate the isotherms. The specific surface area was determined by the Brunauer–Emmett–Teller (BET) method in the relative pressure (P/P_0_) range 0.01–0.25. The surface area of micropores was determined in the 0.15–0.50 P/P_0_ interval using V-t method. The pore size distribution was obtained using the BJH (Barrett-Joyner-Halenda) method and density functional theory method (DFT). The total pore volumes were determined using the point closest to 1 for the relative pressure P/P_0_. Surface fractal dimension was determined by FHH (Frenkel-Halsey-Hill) method from the adsorption branch.

Thermal measurements were performed on a Setaram LabsysEvo (Lyon, France) TG-DSC system, in flowing (90 mL/min) high purity (99.999%) synthetic air (20% O_2_ in N_2_) atmosphere. Samples were weighed into 100 μL Al_2_O_3_ crucibles (the reference cell was empty) and were heated from 25 °C to 800 °C with a heating rate of 10 °C/min. The obtained data was blank corrected and further processed with the thermoanalyzer’s processing software (Calisto Processing, ver. 2.08, Wheaton, IL, USA).

The morphology and surface of the prepared materials have been analyzed by scanning electron microscopy using a LEO 1540XB system (Carl Zeiss, Jena, Germany) equipped with an Ultimax 40 energy dispersive spectroscopy (EDS) Si drift detector (Oxford Instruments, Abingdon, UK). SEM images were recorded at different magnifications up to 20,000× using 2.0 kV accelerating voltage. During EDS analysis an accelerating voltage of 5 kV was used.

## Figures and Tables

**Table 3:** Textural parameters of samples obtained after the extraction of ILs with ethanol.

Sample	S_BET_ [m^2^/g]	S_t-plot_ [m^2^/g]	d_DFT_ [nm]	d_BJH ads_ [nm]	d_BJH des_ [nm]	Vt (cm^3^/g)	d_f_ FHH
IM-0	273	193	2.1	3.4	3.8	0.14	2.7
IM-BF4-0.1-e	252	-	10.1	12.1	7.8	0.87	1.31
IM-BF4-0.3-e	251	-	11.3	17.1	9.7	0.89	1.24
IM-BF4-0.5-e	211	-	14.5	23.1	12.6	0.12	1.17

**Table 4:** Parameter values obtained by least squares fitting the SANS data of the nanocomposites.

Sample	Guinier Term		Power law		*Bg* (cm^−1^)
	*A*	*Rg* (nm)	*B*	*P*	
IM-BF4-0.1	75.0 ± 0.9	7.12 ± 0.06	6 × 10^−5^ ± 0.2·10^−5^	3.98 ± 0.01	0.05 ± 0.01
IM-BF4-0.3	6.4 ± 0.4	4.61 ± 0.05	11 × 10^−5^ ± 2·10^−5^	3.73 ± 0.08	0.22 ± 0.01
IM-BF4-0.5	5.8 ± 0.1	5.64 ± 0.06	30 × 10^−5^ ± 3·10^−5^	3.07 ± 0.04	0.23 ± 0.01

**Table 5:** Xerogels samples compositions.

Samples	IL/Si Molar Ratio	IL [g]	TEOS [g]	Total Water [g]
IM-0	0	0	3.81	9.775
IM-BF4-0.1	0.1	0.414	3.81	9.775
IM-BF4-0.3	0.3	1.24	3.81	9.775
IM-BF4-0.5	0.5	2.068	3.81	9.775
IM-Cl-0.1	0.1	0.319	3.81	9.775
IM-Cl-0.3	0.3	0.959	3.81	9.775
IM-Cl-0.5	0.5	1.598	3.81	9.775

## Data Availability

Not applicable.

## Supplementary Materials

The following supporting information can be downloaded at: https://www.mdpi.com/article/10.3390/gels8060388/s1, Figure S1: IM-0; Figure S2: IM-BF4-0.1; Figure S3: IM-BF4-0.3; Figure S4: IM-BF4-0.5; Figure S5: IM-Cl-0.1; Figure S6: IM-Cl-0.3; Figure S7: IM-BF4-0.5; Figure S8: EDS spectra of samples IM-BF4-0.1, IM-BF4-0.3, IM-BF4-0.5; Figure S9: EDS spectra of samples IM-Cl-0.1, IM-Cl-0.3, IM-Cl-0.5.
