# Supplementary material for: Imidazolium Ionic Liquids as Designer Solvents Confined in Silica Nanopores
Source: Gels. 2022 Jun 19;8(6):388. doi: 10.3390/gels8060388 (PMC9222874; doi:10.3390/gels8060388)
Supplement: Supplementary file 1 [file gels-08-00388-s001.zip › gels-1757171-supplementary.pdf]

Supplementary Materials

Imidazolium ionic liquids as designer solvents confined in silica nanopores

Ana-Maria Putz, Adél Len, László Trif, Zsolt Endre Horváth and László Almásy

Content

1. SEM micrographs of the IL – silica nanocomposites

2. EDS spectra of the IL – silica nanocomposites

1. SEM micrographs of the IL – silica nanocomposites

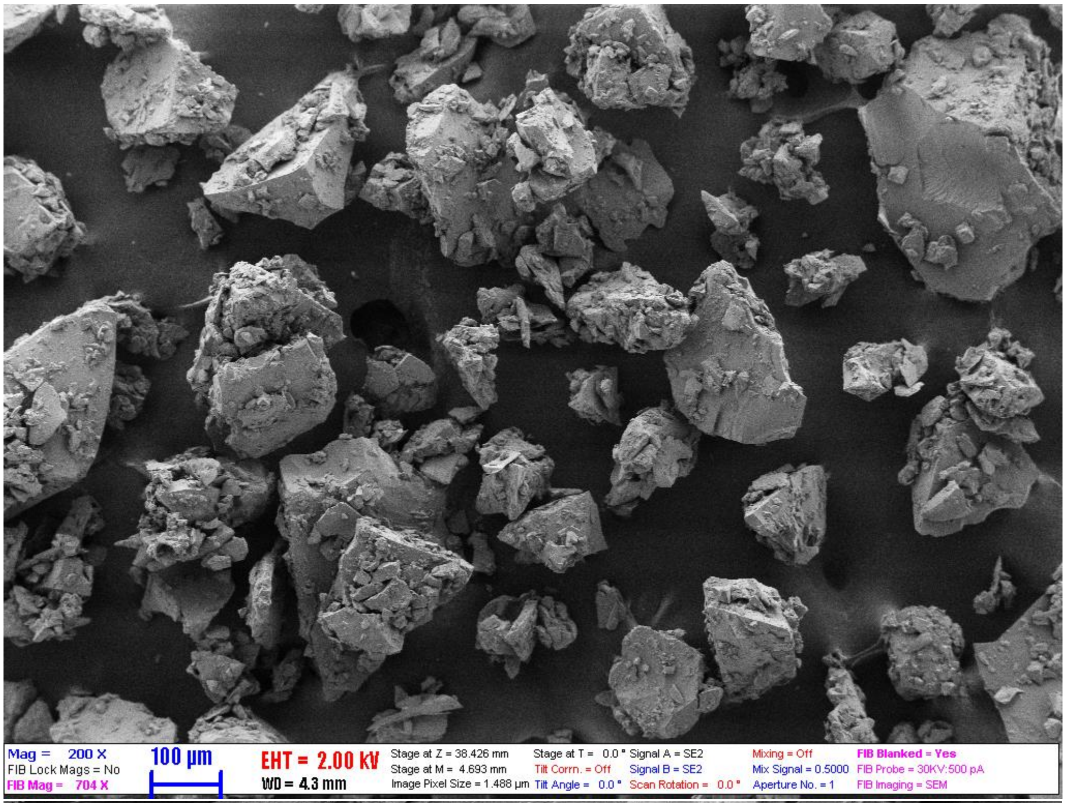
Mag = 200 X
FIB Lock Mags = No
FIB Mag = 704 X
100 µm
EHT = 2.00 kV
WD = 4.3 mm
Stage at Z = 38.426 mm
Stage at M = 4.693 mm
Image Pixel Size = 1.488 µm
Stage at T = 0.0 °
Tilt Corrn. = Off
Tilt Angle = 0.0 °
Signal A = SE2
Signal B = SE2
Scan Rotation = 0.0 °
Mixing = Off
Mix Signal = 0.5000
Aperture No. = 1
FIB Blanked = Yes
FIB Probe = 30KV:500 pA
FIB Imaging = SEM

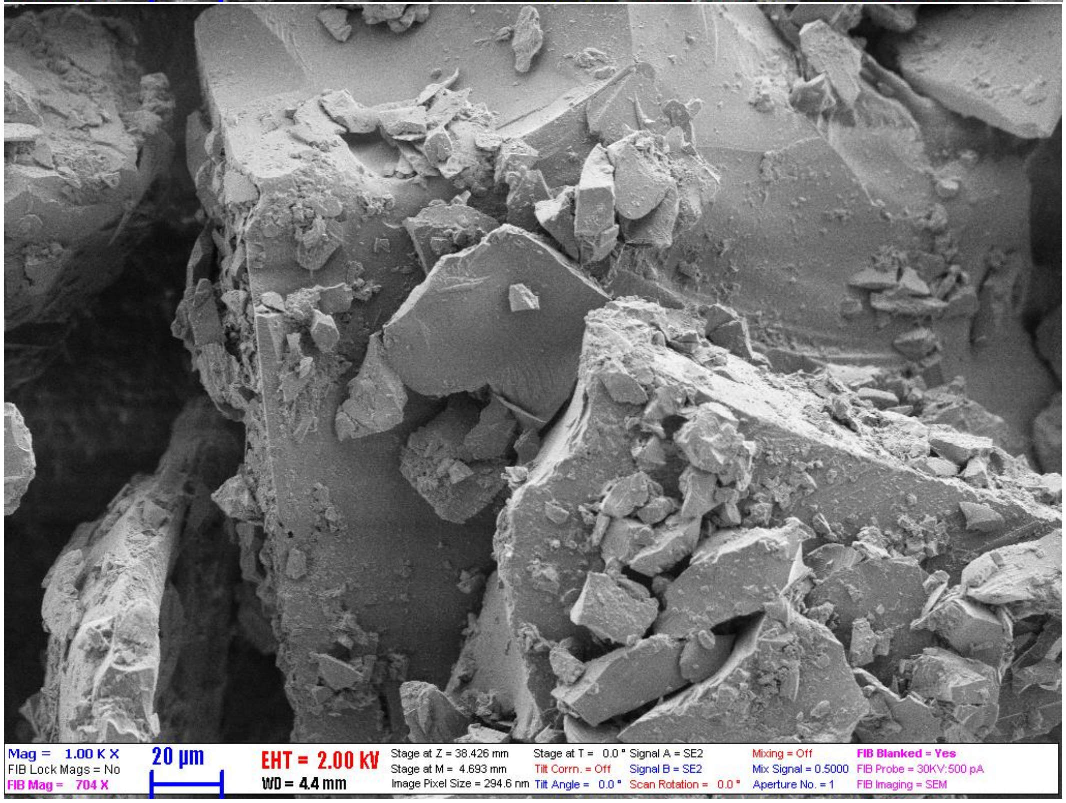
Mag = 1.00 K X
FIB Lock Mags = No
FIB Mag = 704 X
20 µm
EHT = 2.00 kV
WD = 4.4 mm
Stage at Z = 38.426 mm
Stage at M = 4.693 mm
Image Pixel Size = 294.6 nm
Stage at T = 0.0 °
Tilt Corrn. = Off
Tilt Angle = 0.0 °
Signal A = SE2
Signal B = SE2
Scan Rotation = 0.0 °
Mixing = Off
Mix Signal = 0.5000
Aperture No. = 1
FIB Blanked = Yes
FIB Probe = 30KV:500 pA
FIB Imaging = SEM

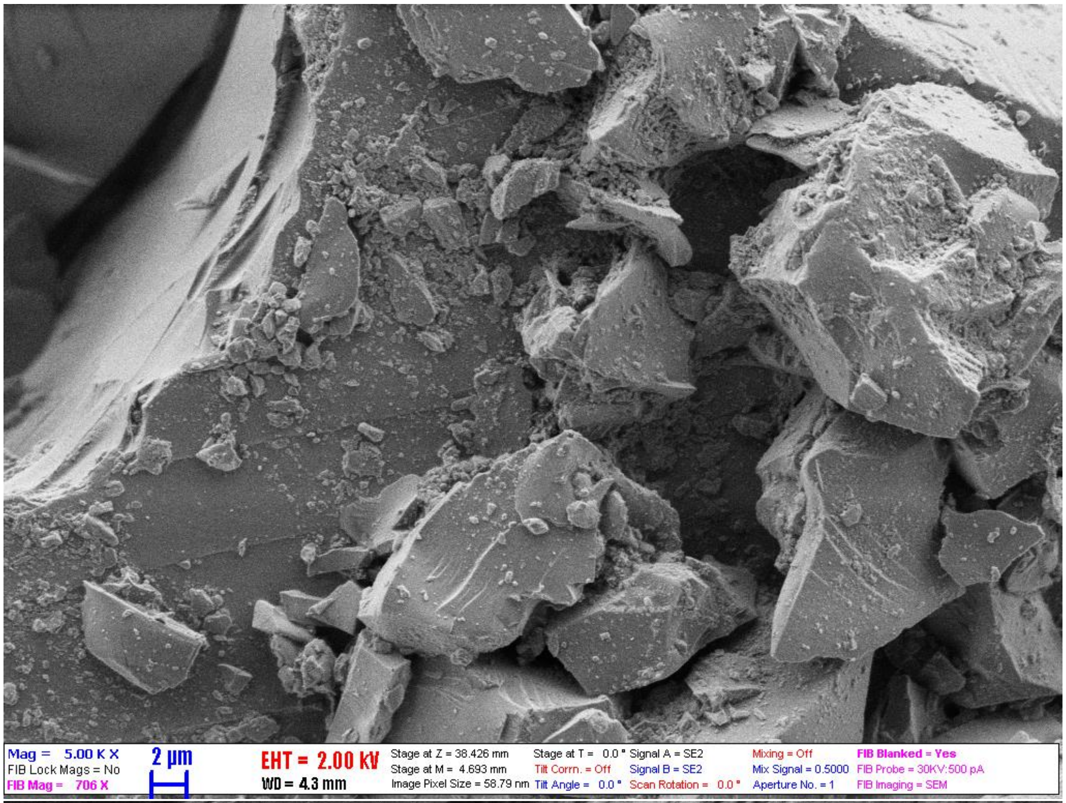
Mag = 5.00 K X
FIB Lock Mags = No
FIB Mag = 706 X
2 µm
EHT = 2.00 kV
WD = 4.3 mm
Stage at Z = 38.426 mm
Stage at M = 4.693 mm
Image Pixel Size = 58.79 nm
Stage at T = 0.0 °
Tilt Corrn. = Off
Tilt Angle = 0.0 °
Signal A = SE2
Signal B = SE2
Scan Rotation = 0.0 °
Mixing = Off
Mix Signal = 0.5000
Aperture No. = 1
FIB Blanked = Yes
FIB Probe = 30KV:500 pA
FIB Imaging = SEM

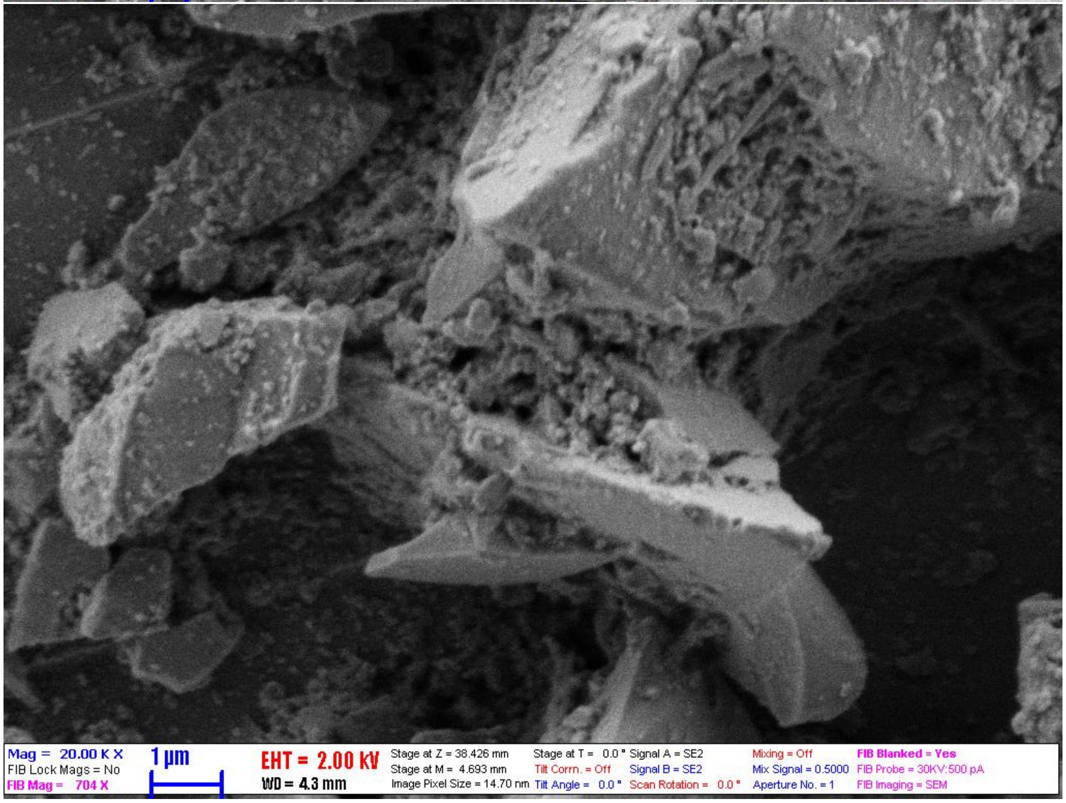
Mag = 20.00 K X
FIB Lock Mags = No
FIB Mag = 704 X
1 µm
EHT = 2.00 kV
WD = 4.3 mm
Stage at Z = 38.426 mm
Stage at M = 4.693 mm
Image Pixel Size = 14.70 nm
Stage at T = 0.0 °
Tilt Corrn. = Off
Tilt Angle = 0.0 °
Signal A = SE2
Signal B = SE2
Scan Rotation = 0.0 °
Mixing = Off
Mix Signal = 0.5000
Aperture No. = 1
FIB Blanked = Yes
FIB Probe = 30KV:500 pA
FIB Imaging = SEM

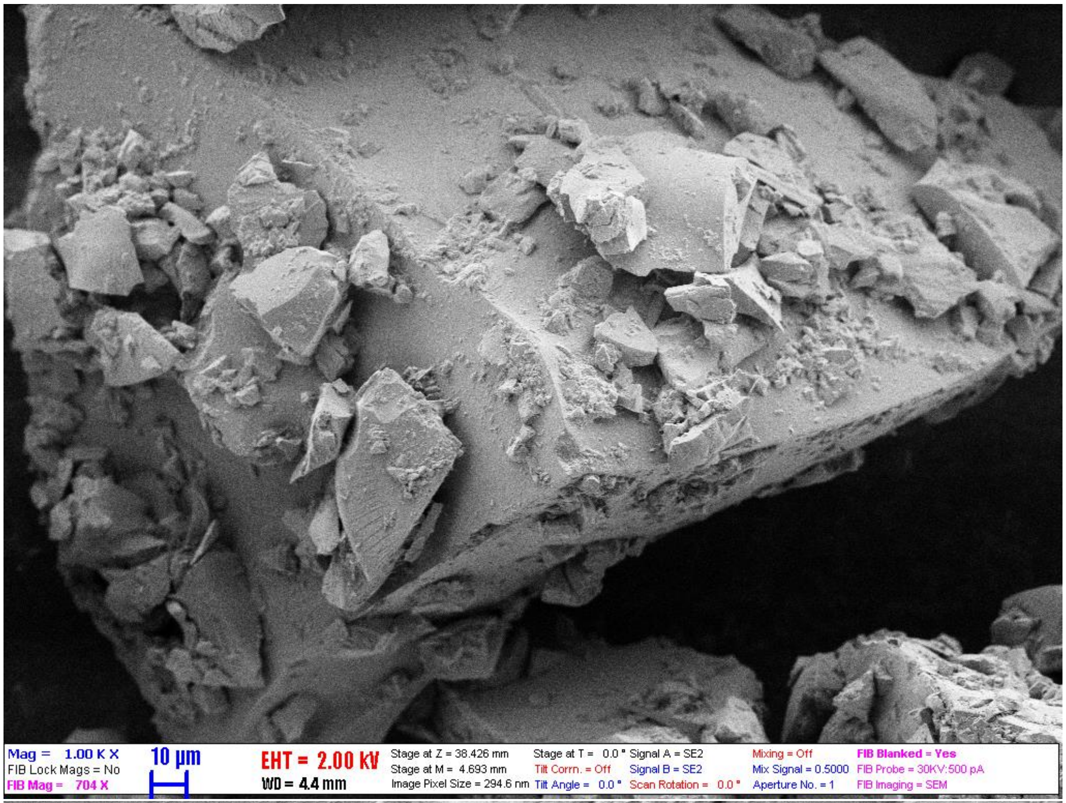
Mag = 1.00 K X
FIB Lock Mags = No
FIB Mag = 704 X
10 µm
EHT = 2.00 kV
WD = 4.4 mm
Stage at Z = 38.426 mm
Stage at M = 4.693 mm
Image Pixel Size = 294.6 nm
Stage at T = 0.0 °
Tilt Corrn. = Off
Tilt Angle = 0.0 °
Signal A = SE2
Signal B = SE2
Scan Rotation = 0.0 °
Mixing = Off
Mix Signal = 0.5000
Aperture No. = 1
FIB Blanked = Yes
FIB Probe = 30KV:500 pA
FIB Imaging = SEM

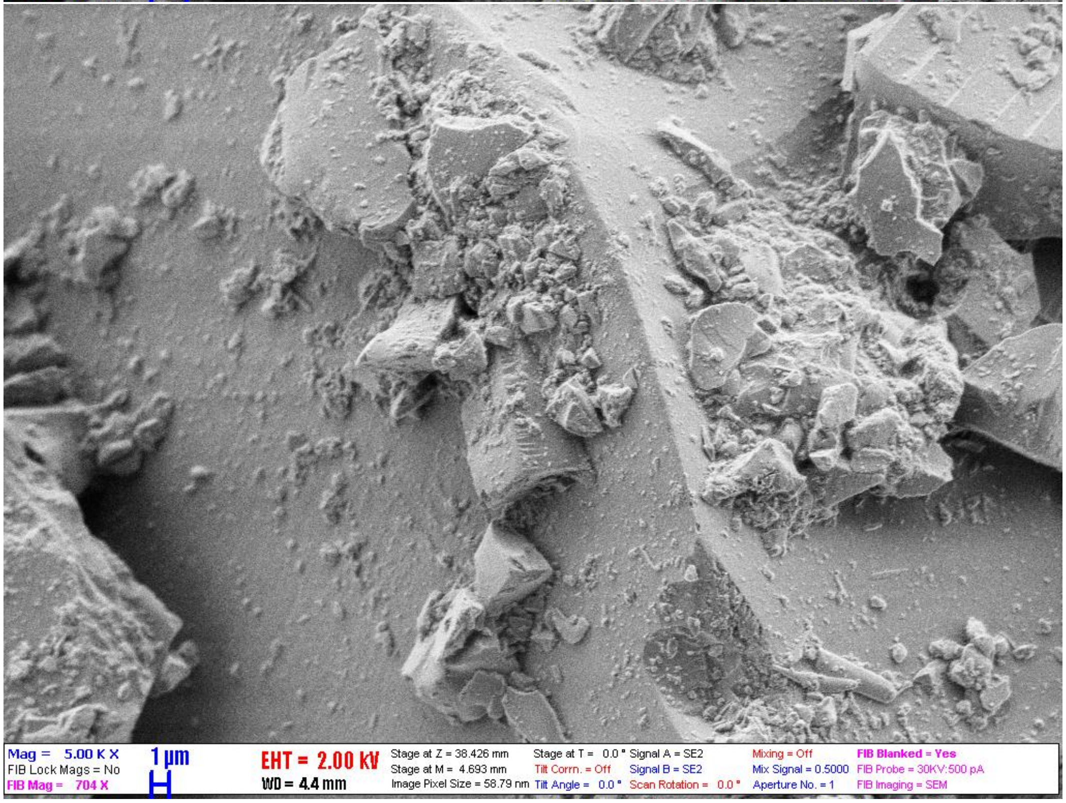
Mag = 5.00 K X
FIB Lock Mags = No
FIB Mag = 704 X
1 µm
EHT = 2.00 kV
WD = 4.4 mm
Stage at Z = 38.426 mm
Stage at M = 4.693 mm
Image Pixel Size = 58.79 nm
Stage at T = 0.0 °
Tilt Corrn. = Off
Tilt Angle = 0.0 °
Signal A = SE2
Signal B = SE2
Scan Rotation = 0.0 °
Mixing = Off
Mix Signal = 0.5000
Aperture No. = 1
FIB Blanked = Yes
FIB Probe = 30KV:500 pA
FIB Imaging = SEM

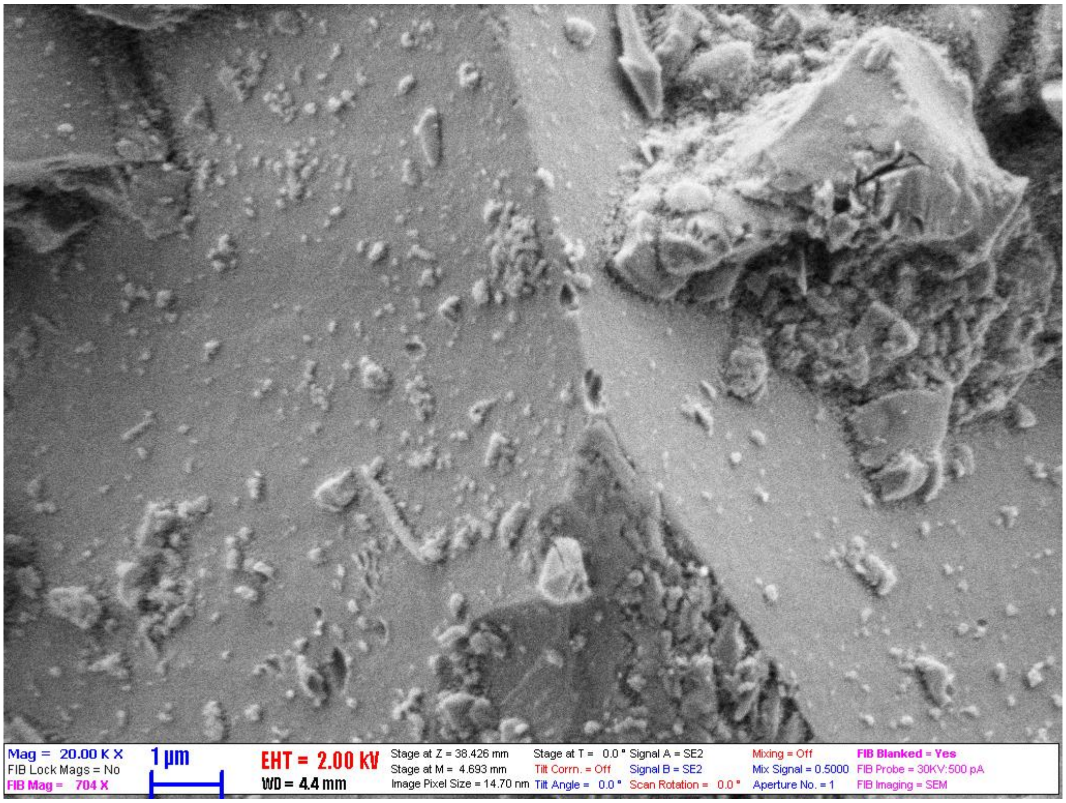

Figure S1 **IM-0**

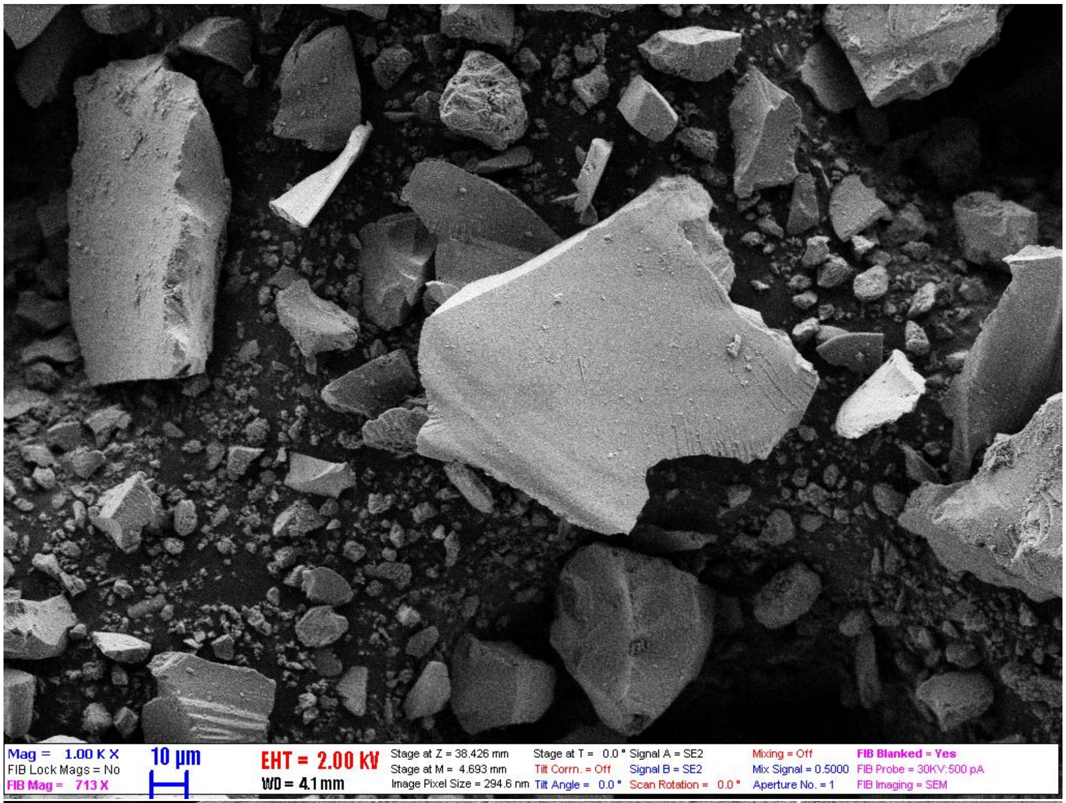
Mag = 1.00 K X
FIB Lock Mags = No
FIB Mag = 713 X
10 µm
EHT = 2.00 kV
WD = 4.1 mm
Stage at Z = 38.426 mm
Stage at M = 4.693 mm
Image Pixel Size = 294.6 nm
Stage at T = 0.0 °
Tilt Corrn. = Off
Tilt Angle = 0.0 °
Signal A = SE2
Signal B = SE2
Scan Rotation = 0.0 °
Mixing = Off
Mix Signal = 0.5000
Aperture No. = 1
FIB Blanked = Yes
FIB Probe = 30KV:500 pA
FIB Imaging = SEM

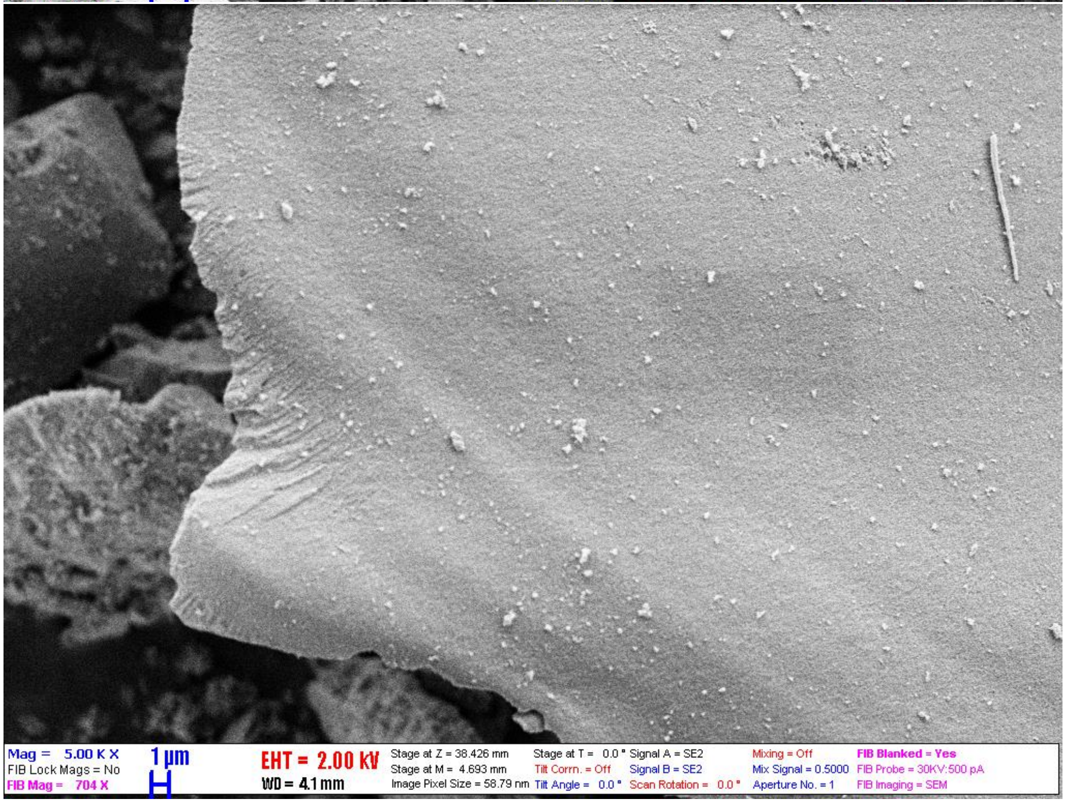
Mag = 5.00 K X
FIB Lock Mags = No
FIB Mag = 704 X
1 µm
EHT = 2.00 kV
WD = 4.1 mm
Stage at Z = 38.426 mm
Stage at M = 4.693 mm
Image Pixel Size = 58.79 nm
Stage at T = 0.0 °
Tilt Corrn. = Off
Tilt Angle = 0.0 °
Signal A = SE2
Signal B = SE2
Scan Rotation = 0.0 °
Mixing = Off
Mix Signal = 0.5000
Aperture No. = 1
FIB Blanked = Yes
FIB Probe = 30KV:500 pA
FIB Imaging = SEM

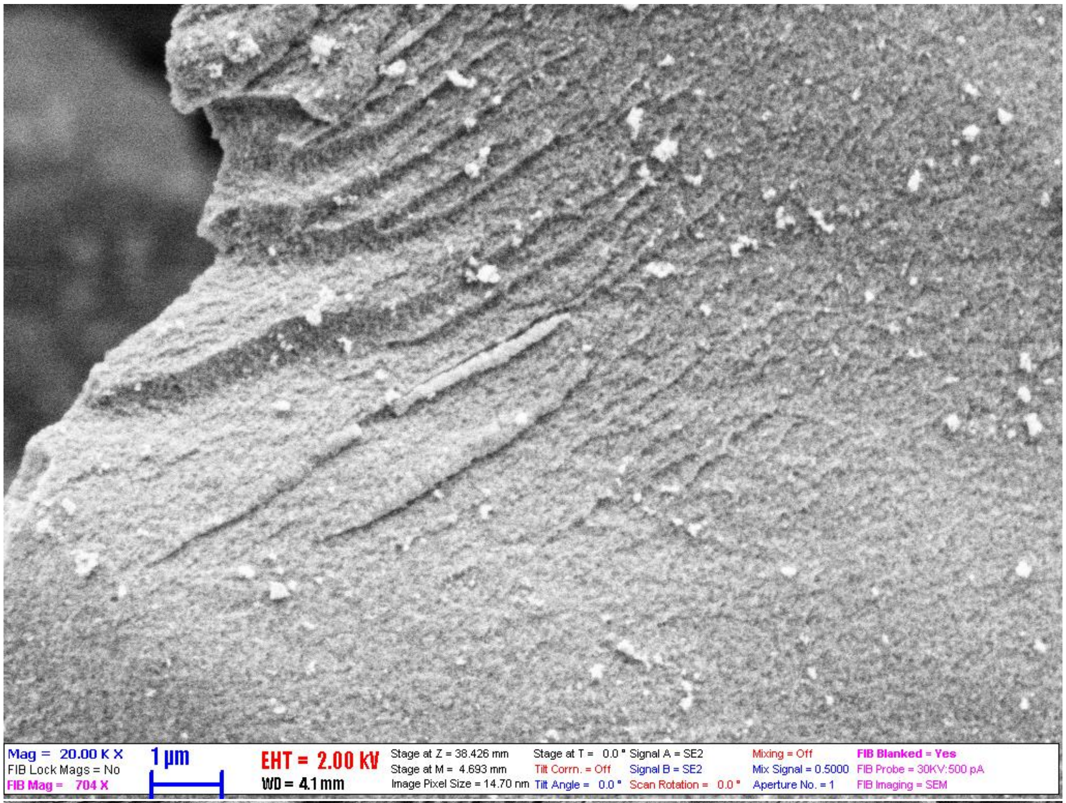
Mag = 20.00 K X
FIB Lock Mags = No
FIB Mag = 704 X
1 µm
EHT = 2.00 kV
WD = 4.1 mm
Stage at Z = 38.426 mm
Stage at M = 4.693 mm
Image Pixel Size = 14.70 nm
Stage at T = 0.0 °
Tilt Corrn. = Off
Tilt Angle = 0.0 °
Signal A = SE2
Signal B = SE2
Scan Rotation = 0.0 °
Mixing = Off
Mix Signal = 0.5000
Aperture No. = 1
FIB Blanked = Yes
FIB Probe = 30KV:500 pA
FIB Imaging = SEM

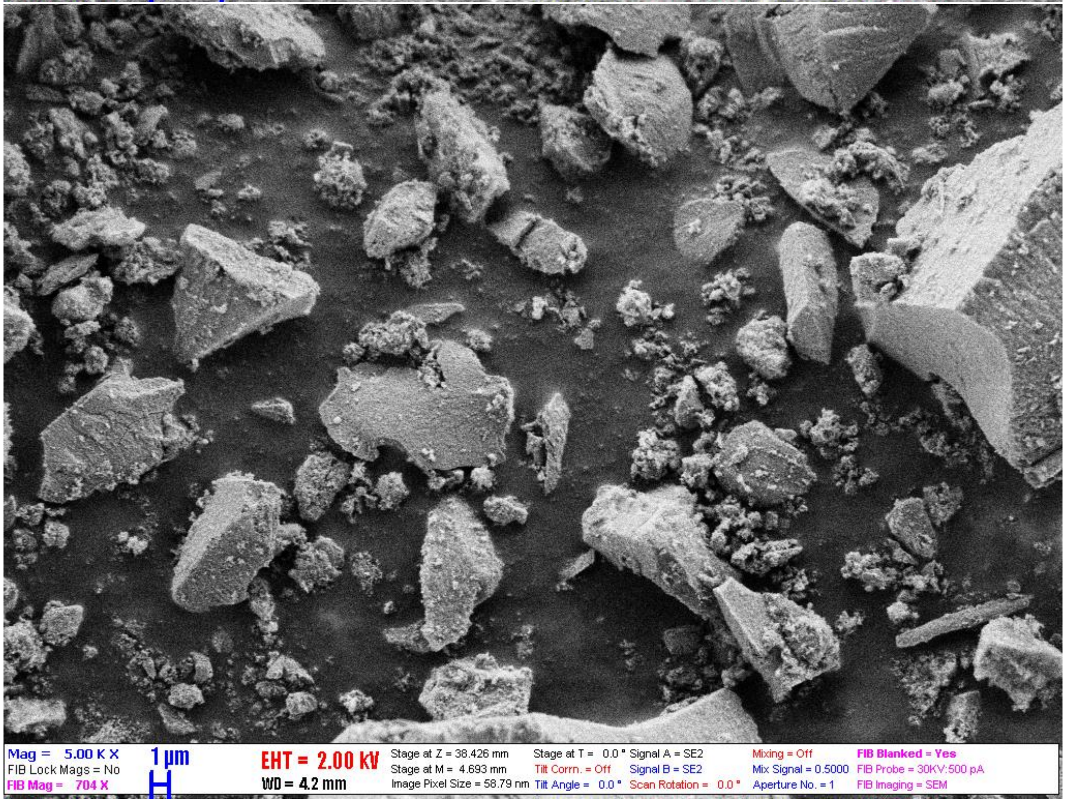
Mag = 5.00 K X
FIB Lock Mags = No
FIB Mag = 704 X
1 µm
EHT = 2.00 kV
WD = 4.2 mm
Stage at Z = 38.426 mm
Stage at M = 4.693 mm
Image Pixel Size = 58.79 nm
Stage at T = 0.0 °
Tilt Corrn. = Off
Tilt Angle = 0.0 °
Signal A = SE2
Signal B = SE2
Scan Rotation = 0.0 °
Mixing = Off
Mix Signal = 0.5000
Aperture No. = 1
FIB Blanked = Yes
FIB Probe = 30KV:500 pA
FIB Imaging = SEM

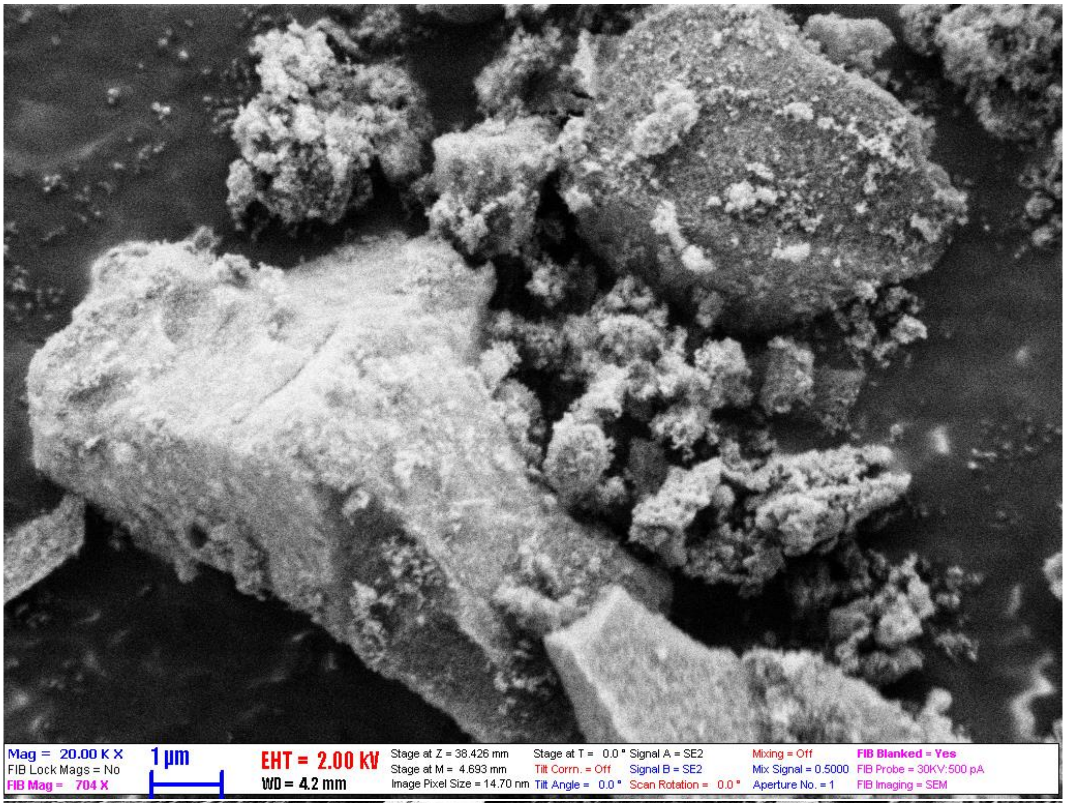
Mag = 20.00 K X
FIB Lock Mags = No
FIB Mag = 704 X
1 µm
EHT = 2.00 kV
WD = 4.2 mm
Stage at Z = 38.426 mm
Stage at M = 4.693 mm
Image Pixel Size = 14.70 nm
Stage at T = 0.0 °
Tilt Corrn. = Off
Tilt Angle = 0.0 °
Signal A = SE2
Signal B = SE2
Scan Rotation = 0.0 °
Mixing = Off
Mix Signal = 0.5000
Aperture No. = 1
FIB Blanked = Yes
FIB Probe = 30KV:500 pA
FIB Imaging = SEM
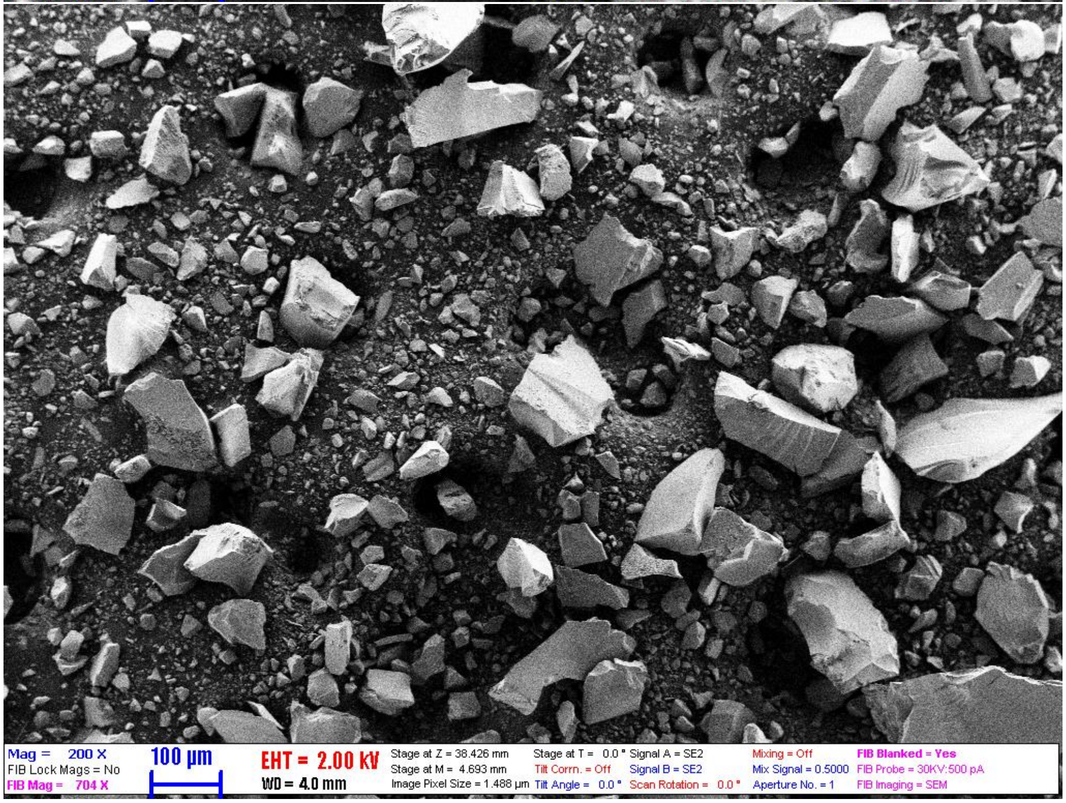
Mag = 200 X
FIB Lock Mags = No
FIB Mag = 704 X
100 µm
EHT = 2.00 kV
WD = 4.0 mm
Stage at Z = 38.426 mm
Stage at M = 4.693 mm
Image Pixel Size = 1.488 µm
Stage at T = 0.0 °
Tilt Corrn. = Off
Tilt Angle = 0.0 °
Signal A = SE2
Signal B = SE2
Scan Rotation = 0.0 °
Mixing = Off
Mix Signal = 0.5000
Aperture No. = 1
FIB Blanked = Yes
FIB Probe = 30KV:500 pA
FIB Imaging = SEM

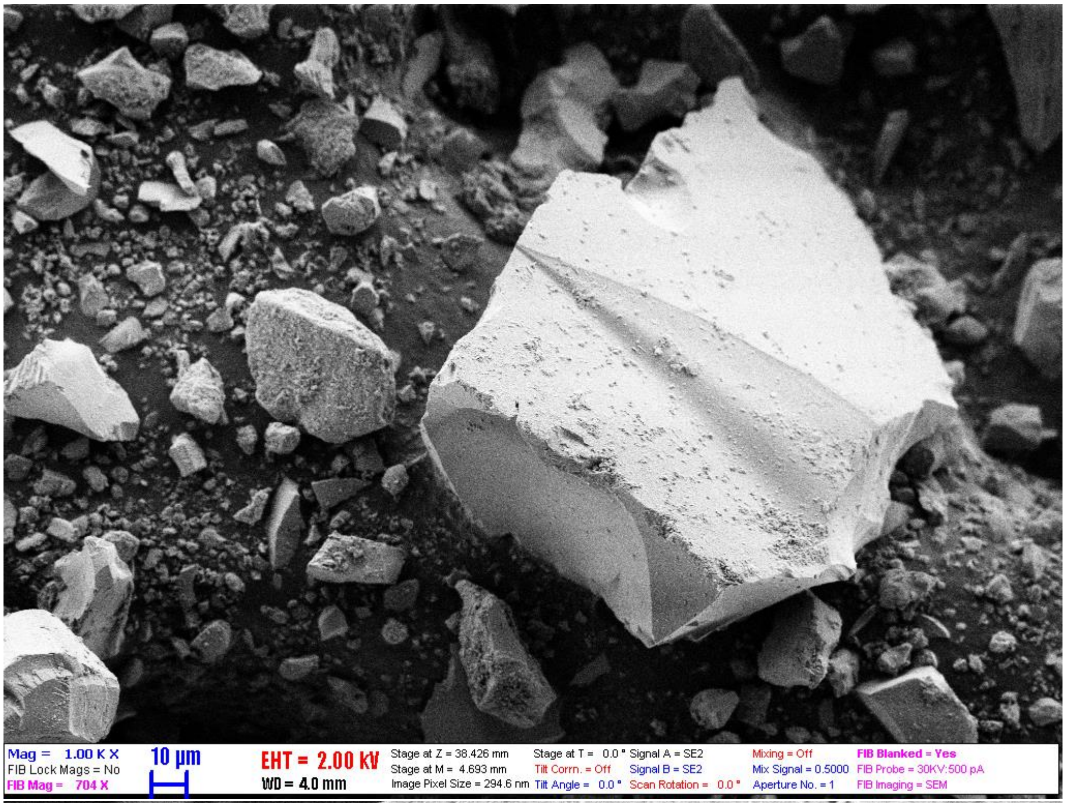
Mag = 1.00 K X
FIB Lock Mags = No
FIB Mag = 704 X
10 µm
EHT = 2.00 kV
WD = 4.0 mm
Stage at Z = 38.426 mm
Stage at M = 4.693 mm
Image Pixel Size = 294.6 nm
Stage at T = 0.0 °
Tilt Corrn. = Off
Tilt Angle = 0.0 °
Signal A = SE2
Signal B = SE2
Scan Rotation = 0.0 °
Mixing = Off
Mix Signal = 0.5000
Aperture No. = 1
FIB Blanked = Yes
FIB Probe = 30KV:500 pA
FIB Imaging = SEM
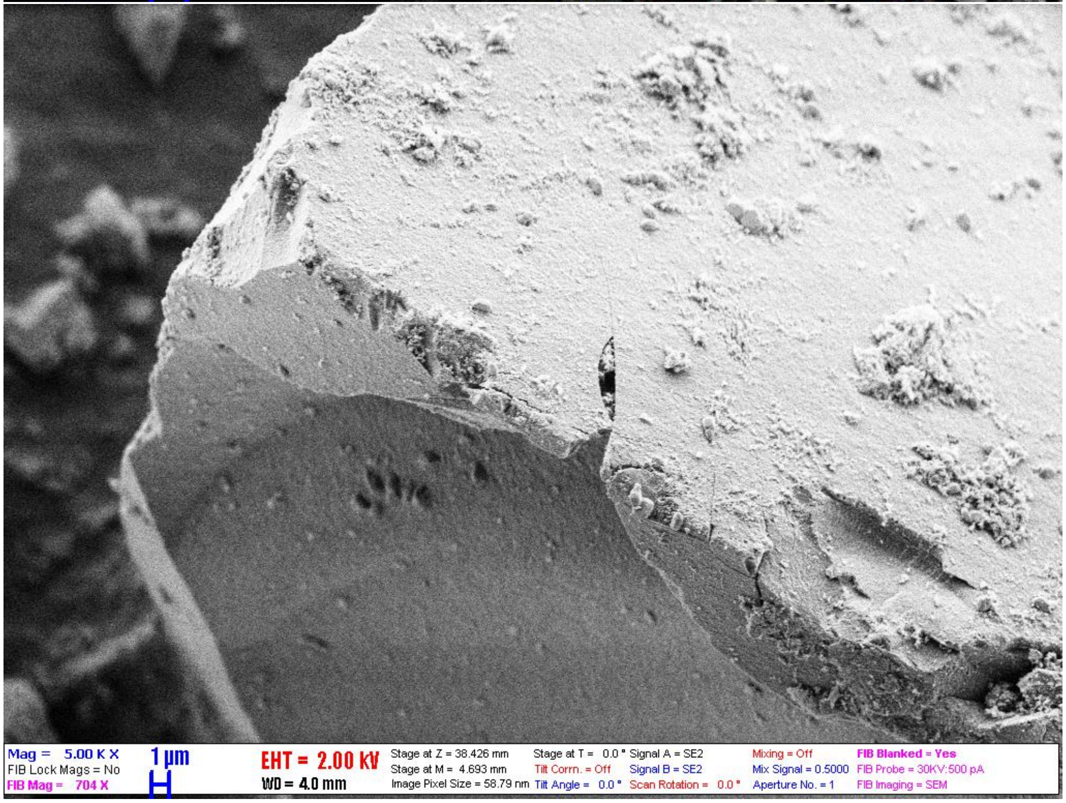
Mag = 5.00 K X
FIB Lock Mags = No
FIB Mag = 704 X
1 µm
EHT = 2.00 kV
WD = 4.0 mm
Stage at Z = 38.426 mm
Stage at M = 4.693 mm
Image Pixel Size = 58.79 nm
Stage at T = 0.0 °
Tilt Corrn. = Off
Tilt Angle = 0.0 °
Signal A = SE2
Signal B = SE2
Scan Rotation = 0.0 °
Mixing = Off
Mix Signal = 0.5000
Aperture No. = 1
FIB Blanked = Yes
FIB Probe = 30KV:500 pA
FIB Imaging = SEM

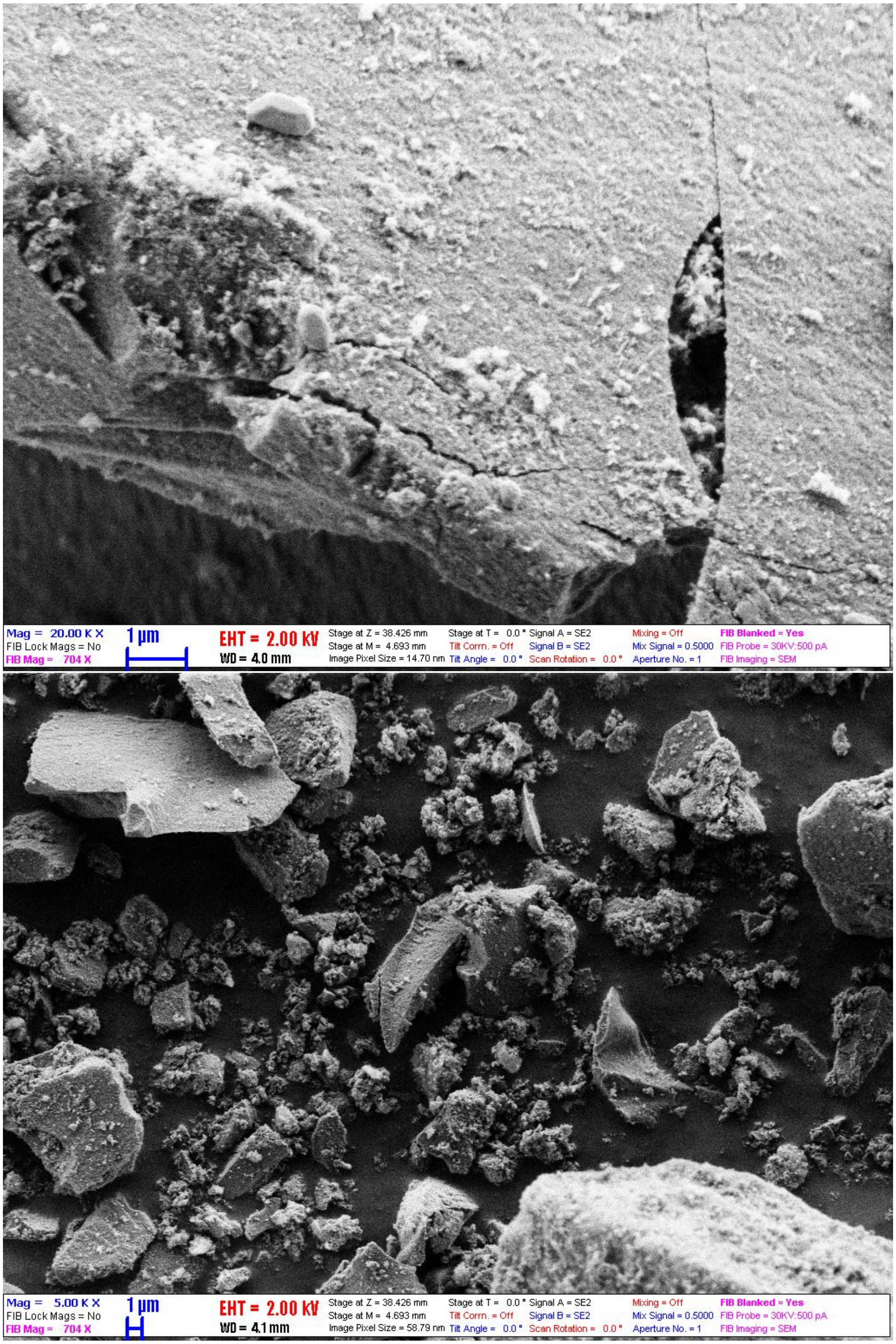

Figure S2. **IM-BF4-0.1**

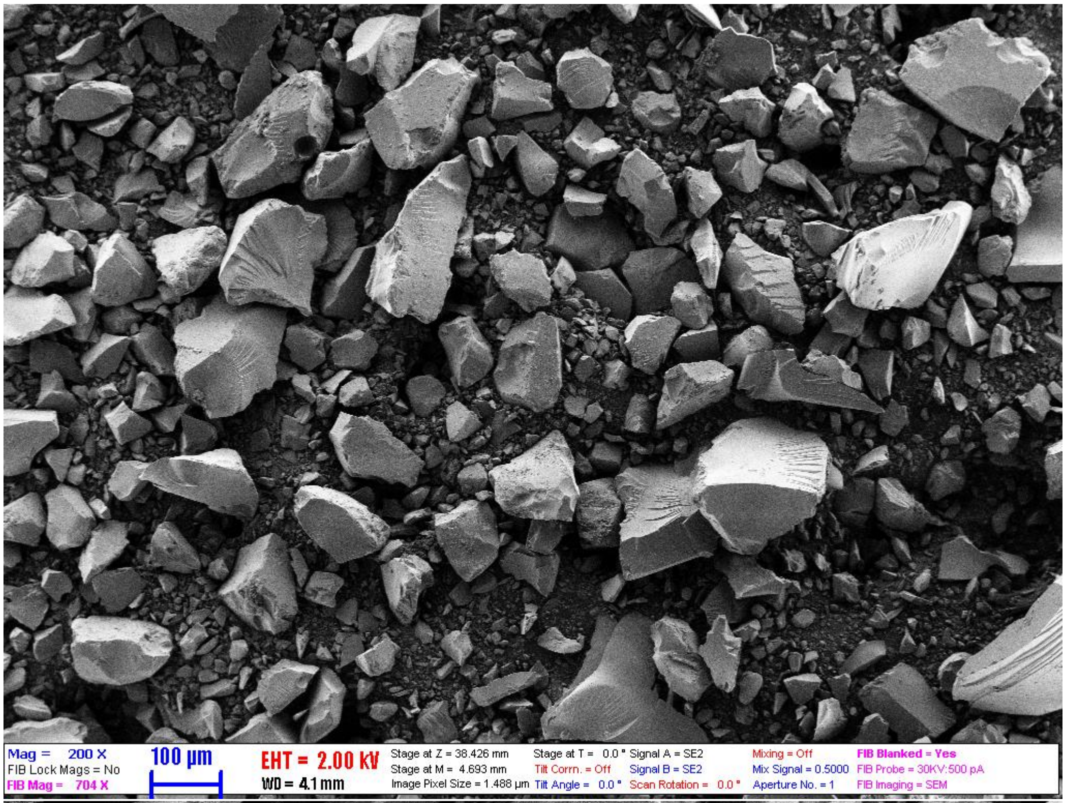
Mag = 200 X
FIB Lock Mags = No
FIB Mag = 704 X
100 µm
EHT = 2.00 kV
WD = 4.1 mm
Stage at Z = 38.426 mm
Stage at M = 4.693 mm
Image Pixel Size = 1.488 µm
Stage at T = 0.0 °
Tilt Corrn. = Off
Tilt Angle = 0.0 °
Signal A = SE2
Signal B = SE2
Scan Rotation = 0.0 °
Mixing = Off
Mix Signal = 0.5000
Aperture No. = 1
FIB Blanked = Yes
FIB Probe = 30KV:500 pA
FIB Imaging = SEM

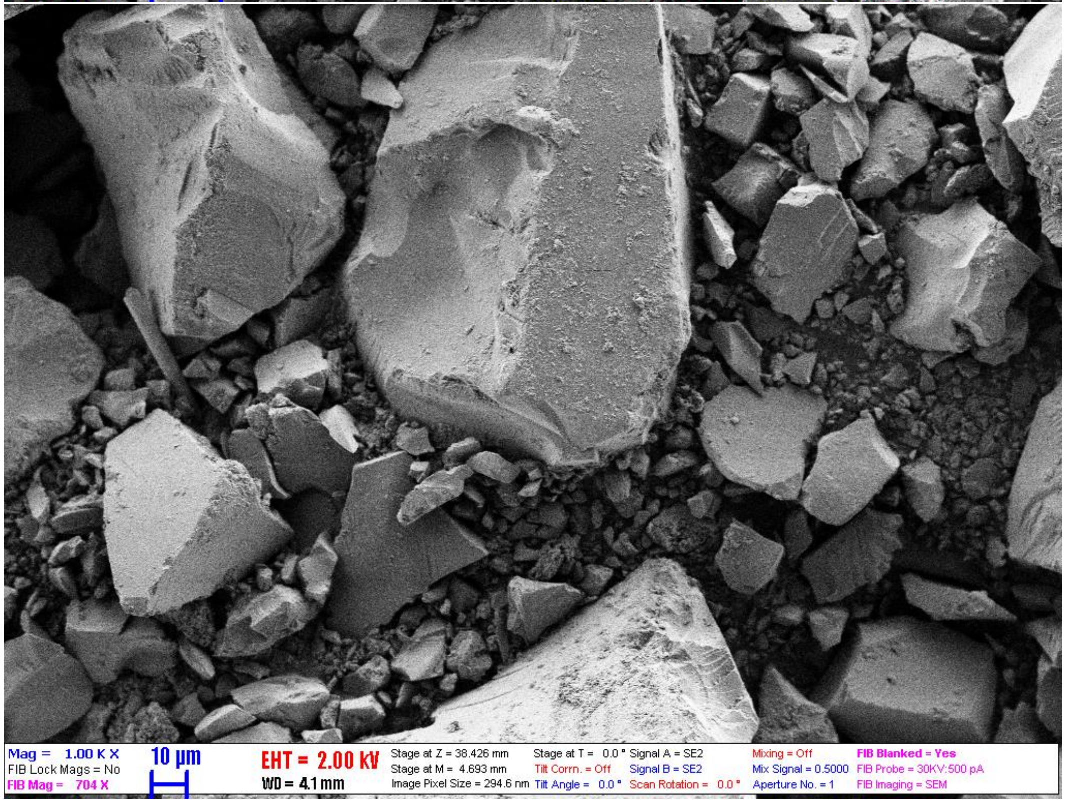
Mag = 1.00 K X
FIB Lock Mags = No
FIB Mag = 704 X
10 µm
EHT = 2.00 kV
WD = 4.1 mm
Stage at Z = 38.426 mm
Stage at M = 4.693 mm
Image Pixel Size = 294.6 nm
Stage at T = 0.0 °
Tilt Corrn. = Off
Tilt Angle = 0.0 °
Signal A = SE2
Signal B = SE2
Scan Rotation = 0.0 °
Mixing = Off
Mix Signal = 0.5000
Aperture No. = 1
FIB Blanked = Yes
FIB Probe = 30KV:500 pA
FIB Imaging = SEM

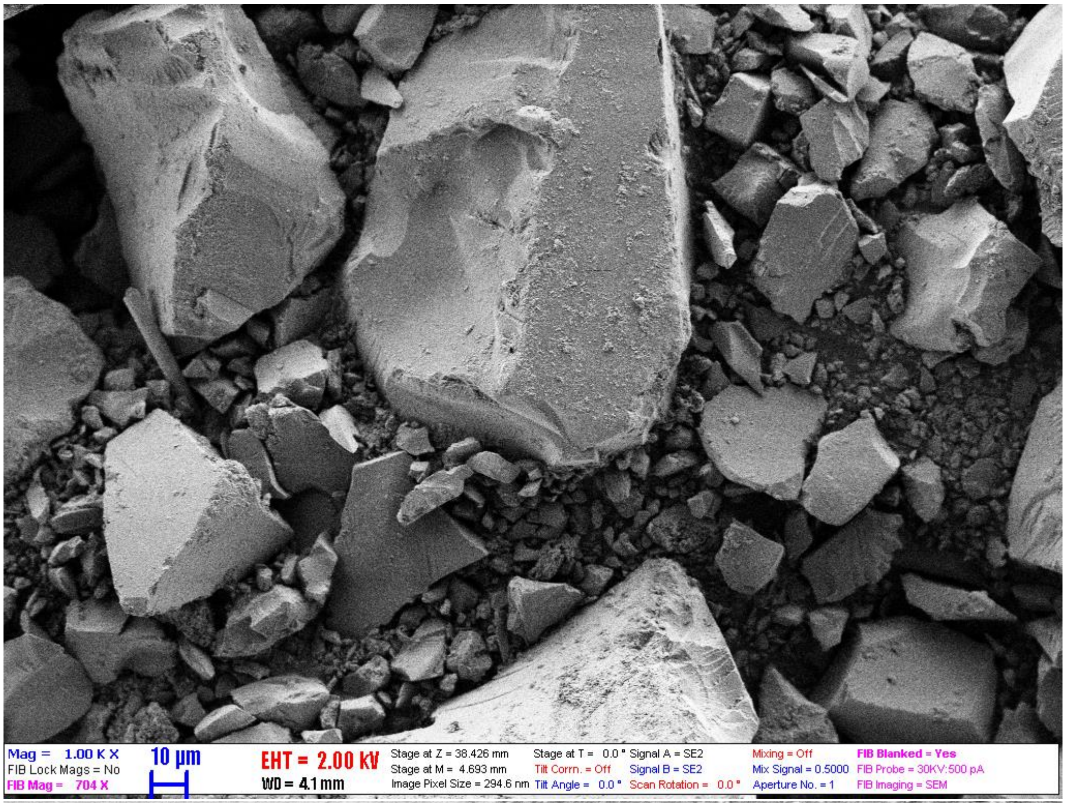
Mag = 1.00 K X
FIB Lock Mags = No
FIB Mag = 704 X
10 µm
EHT = 2.00 kV
WD = 4.1 mm
Stage at Z = 38.426 mm
Stage at M = 4.693 mm
Image Pixel Size = 294.6 nm
Stage at T = 0.0 °
Tilt Corrn. = Off
Tilt Angle = 0.0 °
Signal A = SE2
Signal B = SE2
Scan Rotation = 0.0 °
Mixing = Off
Mix Signal = 0.5000
Aperture No. = 1
FIB Blanked = Yes
FIB Probe = 30KV:500 pA
FIB Imaging = SEM

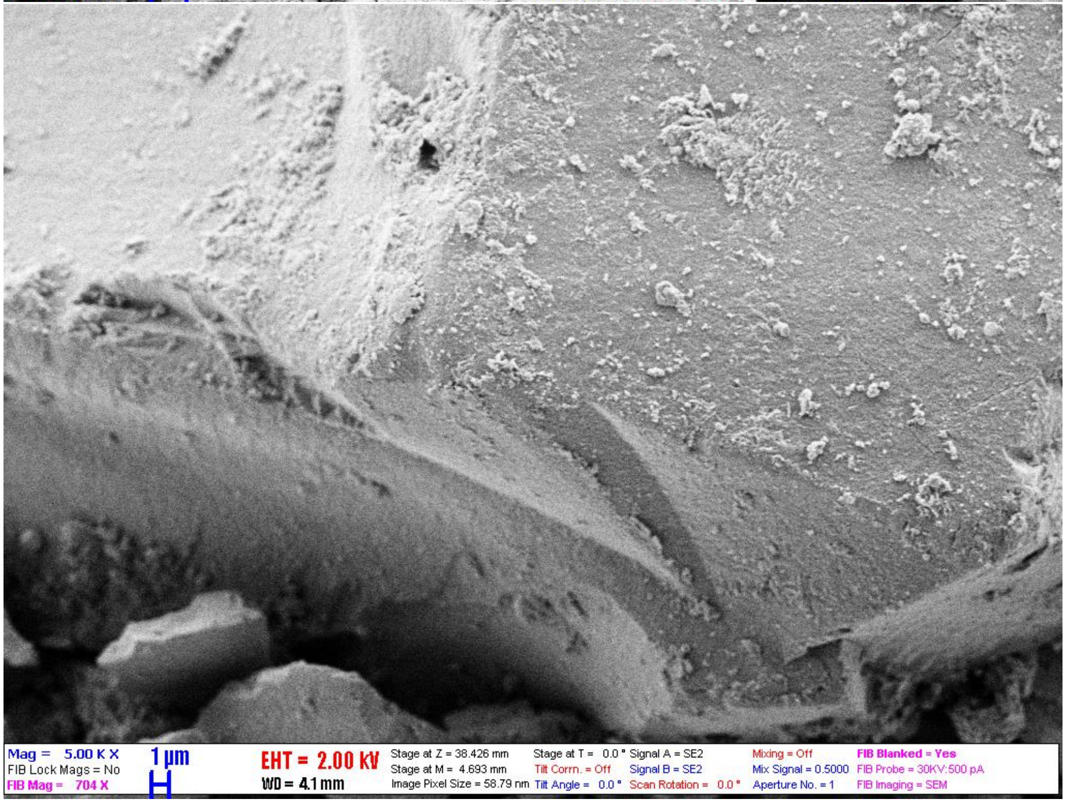
Mag = 5.00 K X
FIB Lock Mags = No
FIB Mag = 704 X
1 µm
EHT = 2.00 kV
WD = 4.1 mm
Stage at Z = 38.426 mm
Stage at M = 4.693 mm
Image Pixel Size = 58.79 nm
Stage at T = 0.0 °
Tilt Corrn. = Off
Tilt Angle = 0.0 °
Signal A = SE2
Signal B = SE2
Scan Rotation = 0.0 °
Mixing = Off
Mix Signal = 0.5000
Aperture No. = 1
FIB Blanked = Yes
FIB Probe = 30KV:500 pA
FIB Imaging = SEM

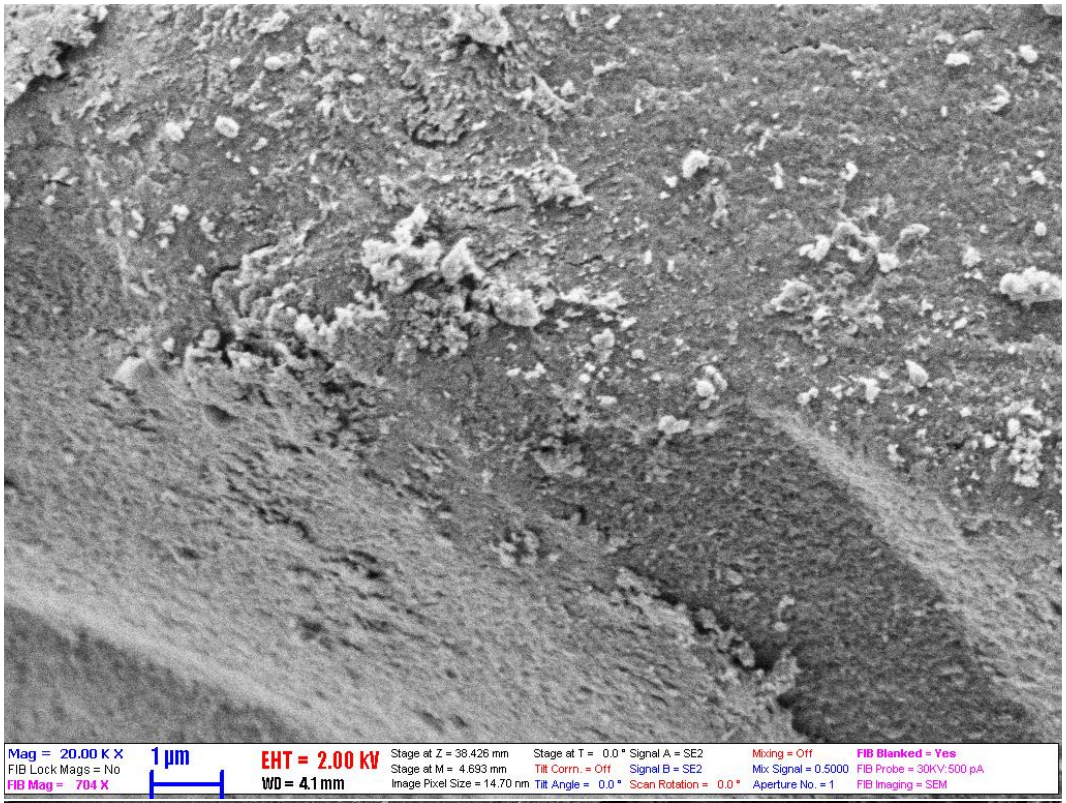
Mag = 20.00 K X
FIB Lock Mags = No
FIB Mag = 704 X
1 µm
EHT = 2.00 kV
WD = 4.1 mm
Stage at Z = 38.426 mm
Stage at M = 4.693 mm
Image Pixel Size = 14.70 nm
Stage at T = 0.0 °
Tilt Corrn. = Off
Tilt Angle = 0.0 °
Signal A = SE2
Signal B = SE2
Scan Rotation = 0.0 °
Mixing = Off
Mix Signal = 0.5000
Aperture No. = 1
FIB Blanked = Yes
FIB Probe = 30KV:500 pA
FIB Imaging = SEM

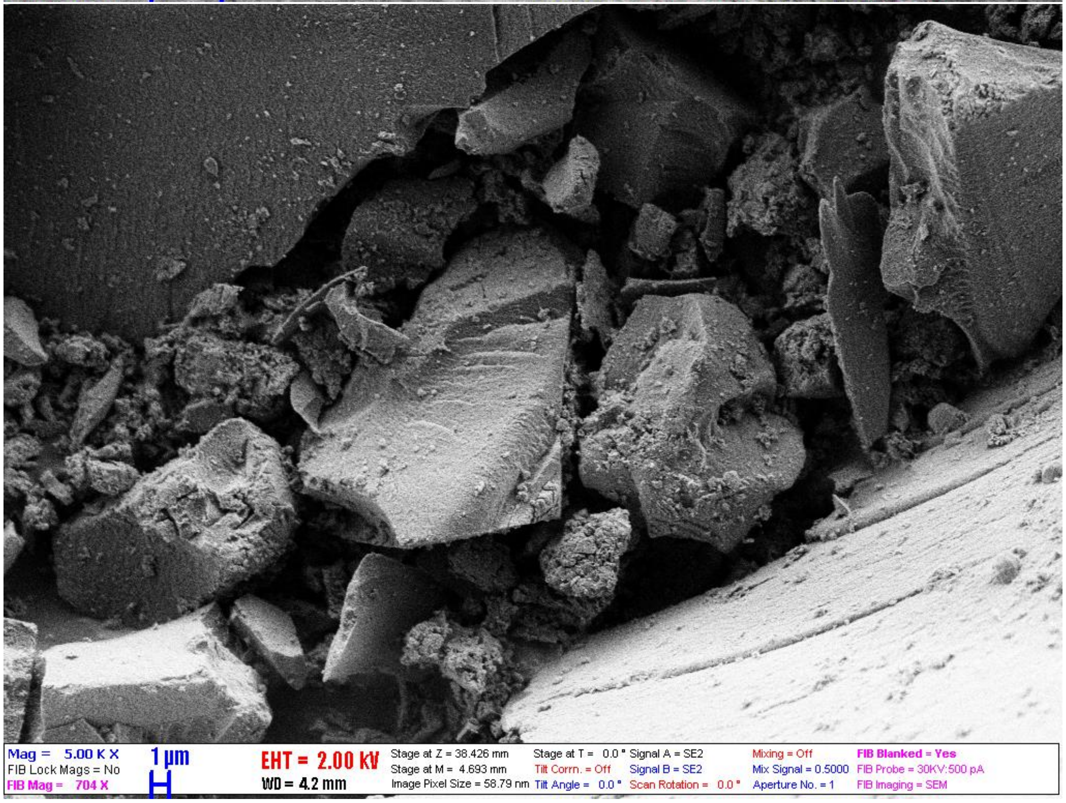
Mag = 5.00 K X
FIB Lock Mags = No
FIB Mag = 704 X
1 µm
EHT = 2.00 kV
WD = 4.2 mm
Stage at Z = 38.426 mm
Stage at M = 4.693 mm
Image Pixel Size = 58.79 nm
Stage at T = 0.0 °
Tilt Corrn. = Off
Tilt Angle = 0.0 °
Signal A = SE2
Signal B = SE2
Scan Rotation = 0.0 °
Mixing = Off
Mix Signal = 0.5000
Aperture No. = 1
FIB Blanked = Yes
FIB Probe = 30KV:500 pA
FIB Imaging = SEM

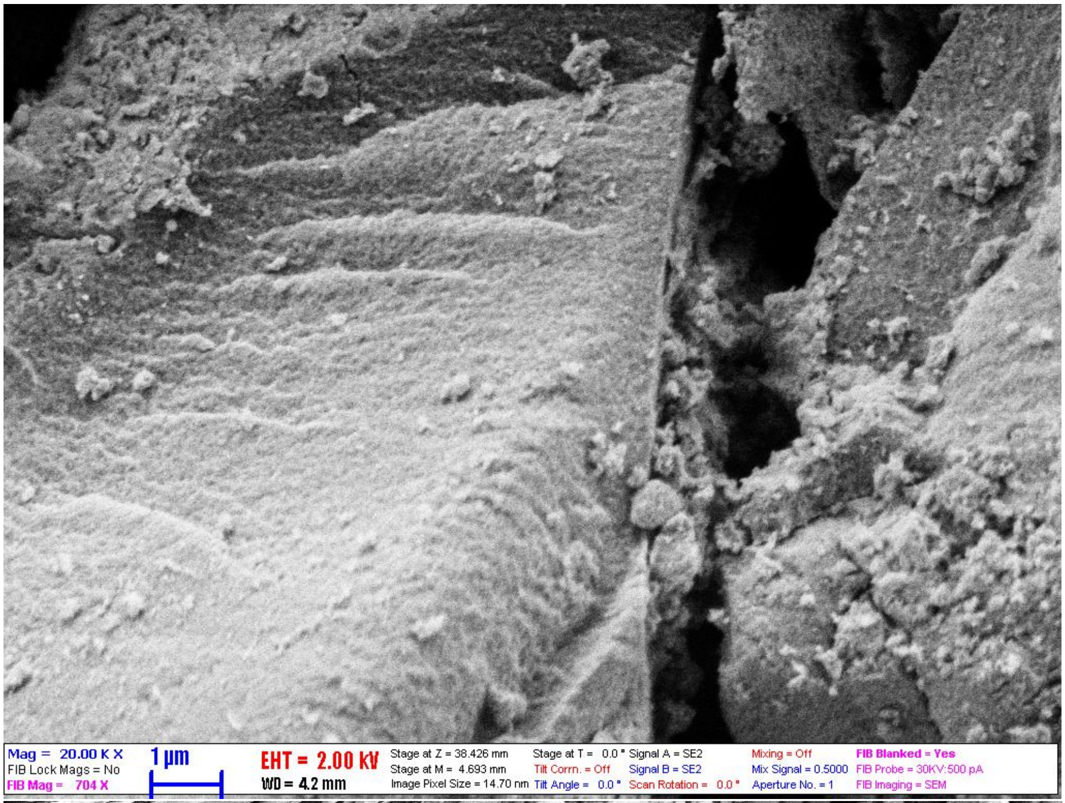
Mag = 20.00 K X
FIB Lock Mags = No
FIB Mag = 704 X
1 µm
EHT = 2.00 kV
WD = 4.2 mm
Stage at Z = 38.426 mm
Stage at M = 4.693 mm
Image Pixel Size = 14.70 nm
Stage at T = 0.0 °
Tilt Corrn. = Off
Tilt Angle = 0.0 °
Signal A = SE2
Signal B = SE2
Scan Rotation = 0.0 °
Mixing = Off
Mix Signal = 0.5000
Aperture No. = 1
FIB Blanked = Yes
FIB Probe = 30KV:500 pA
FIB Imaging = SEM

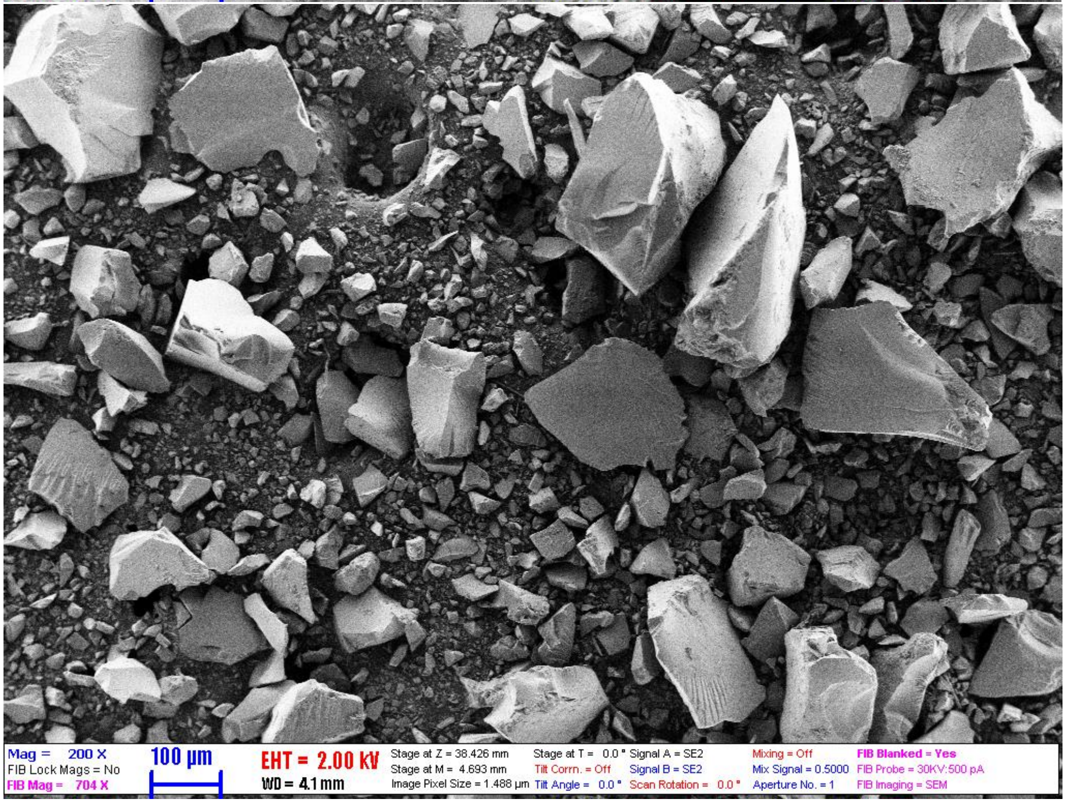
Mag = 200 X
FIB Lock Mags = No
FIB Mag = 704 X
100 µm
EHT = 2.00 kV
WD = 4.1 mm
Stage at Z = 38.426 mm
Stage at M = 4.693 mm
Image Pixel Size = 1.488 µm
Stage at T = 0.0 °
Tilt Corrn. = Off
Tilt Angle = 0.0 °
Signal A = SE2
Signal B = SE2
Scan Rotation = 0.0 °
Mixing = Off
Mix Signal = 0.5000
Aperture No. = 1
FIB Blanked = Yes
FIB Probe = 30KV:500 pA
FIB Imaging = SEM

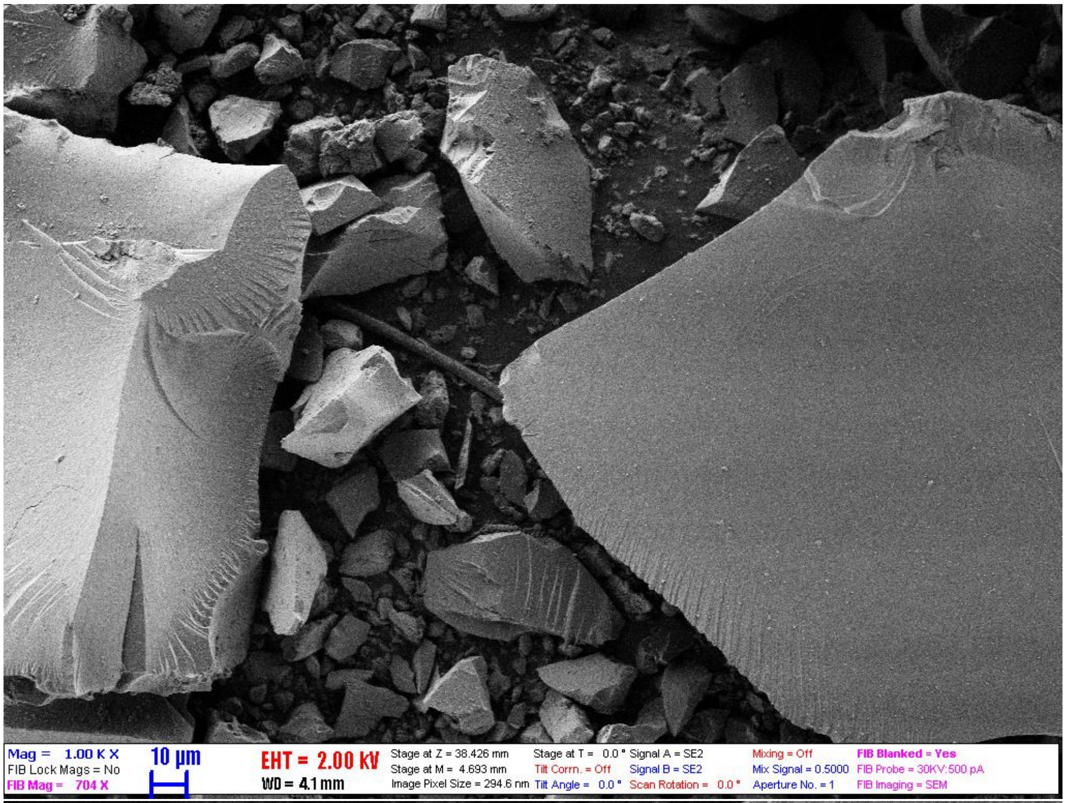
Mag = 1.00 K X
FIB Lock Mags = No
FIB Mag = 704 X
10 µm
EHT = 2.00 kV
WD = 4.1 mm
Stage at Z = 38.426 mm
Stage at M = 4.693 mm
Image Pixel Size = 294.6 nm
Stage at T = 0.0 °
Tilt Corrn. = Off
Tilt Angle = 0.0 °
Signal A = SE2
Signal B = SE2
Scan Rotation = 0.0 °
Mixing = Off
Mix Signal = 0.5000
Aperture No. = 1
FIB Blanked = Yes
FIB Probe = 30KV:500 pA
FIB Imaging = SEM

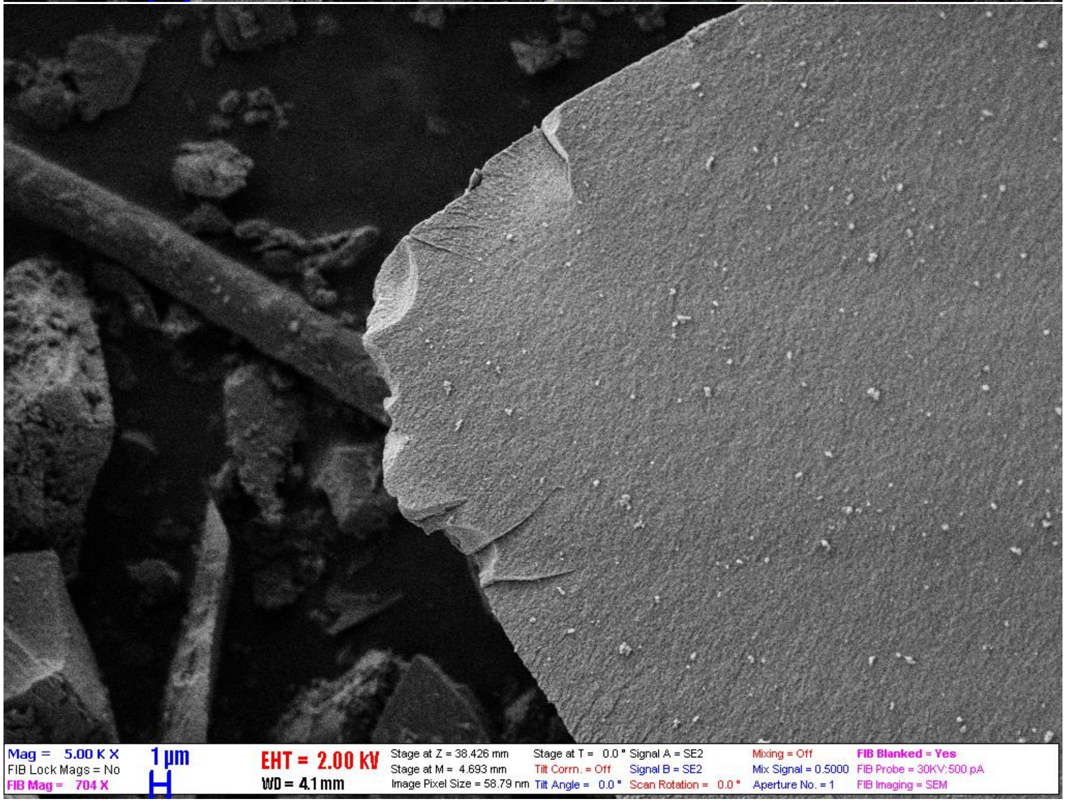
Mag = 5.00 K X
FIB Lock Mags = No
FIB Mag = 704 X
1 µm
EHT = 2.00 kV
WD = 4.1 mm
Stage at Z = 38.426 mm
Stage at M = 4.693 mm
Image Pixel Size = 58.79 nm
Stage at T = 0.0 °
Tilt Corrn. = Off
Tilt Angle = 0.0 °
Signal A = SE2
Signal B = SE2
Scan Rotation = 0.0 °
Mixing = Off
Mix Signal = 0.5000
Aperture No. = 1
FIB Blanked = Yes
FIB Probe = 30KV:500 pA
FIB Imaging = SEM

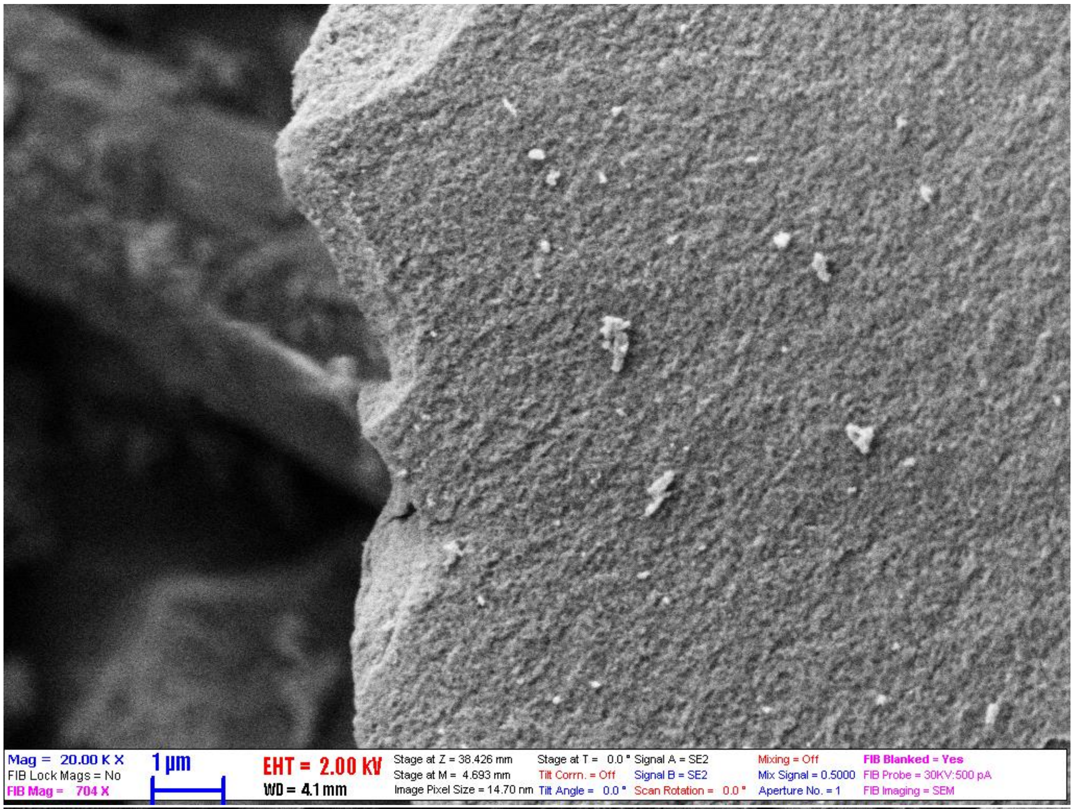
Mag = 20.00 K X
FIB Lock Mags = No
FIB Mag = 704 X
1 µm
EHT = 2.00 kV
WD = 4.1 mm
Stage at Z = 38.426 mm
Stage at M = 4.693 mm
Image Pixel Size = 14.70 nm
Stage at T = 0.0 °
Tilt Corrn. = Off
Tilt Angle = 0.0 °
Signal A = SE2
Signal B = SE2
Scan Rotation = 0.0 °
Mixing = Off
Mix Signal = 0.5000
Aperture No. = 1
FIB Blanked = Yes
FIB Probe = 30KV:500 pA
FIB Imaging = SEM

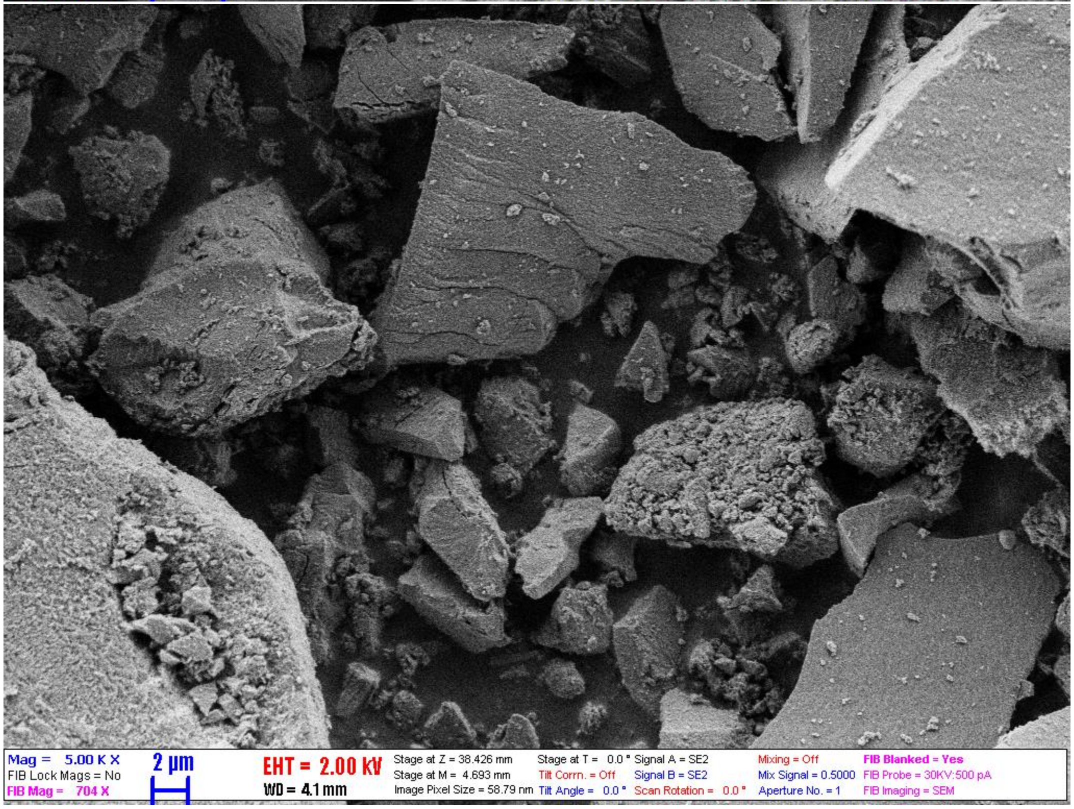
Mag = 5.00 K X
FIB Lock Mags = No
FIB Mag = 704 X
2 µm
EHT = 2.00 kV
WD = 4.1 mm
Stage at Z = 38.426 mm
Stage at M = 4.693 mm
Image Pixel Size = 58.79 nm
Stage at T = 0.0 °
Tilt Corrn. = Off
Tilt Angle = 0.0 °
Signal A = SE2
Signal B = SE2
Scan Rotation = 0.0 °
Mixing = Off
Mix Signal = 0.5000
Aperture No. = 1
FIB Blanked = Yes
FIB Probe = 30KV:500 pA
FIB Imaging = SEM

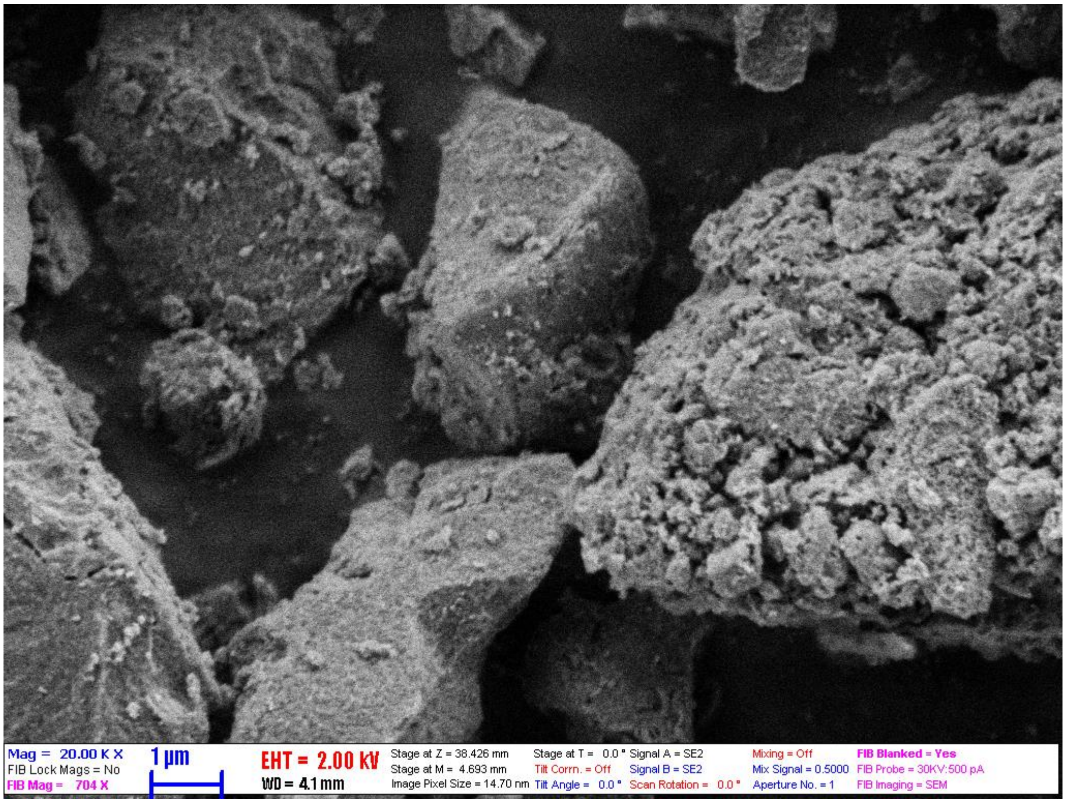

Figure S3 **IM-BF4-0.3**

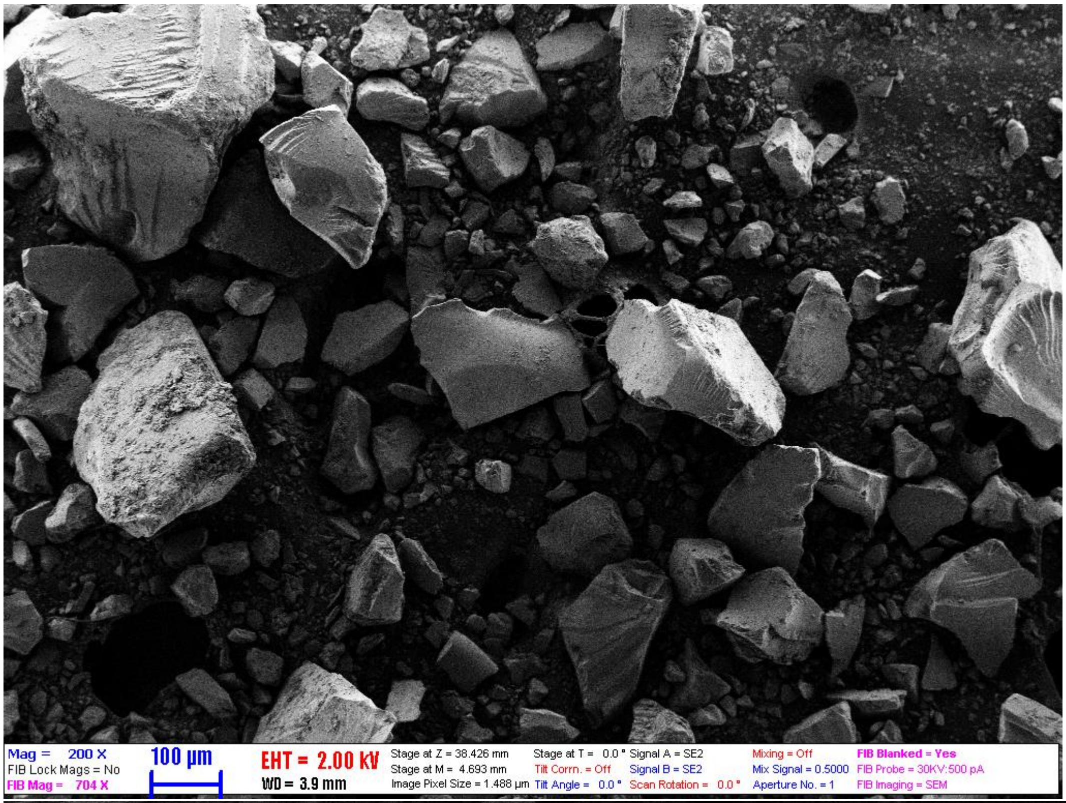
Mag = 200 X
FIB Lock Mags = No
FIB Mag = 704 X
100 µm
EHT = 2.00 kV
WD = 3.9 mm
Stage at Z = 38.426 mm
Stage at M = 4.693 mm
Image Pixel Size = 1.488 µm
Stage at T = 0.0 °
Tilt Corrn. = Off
Tilt Angle = 0.0 °
Signal A = SE2
Signal B = SE2
Scan Rotation = 0.0 °
Mixing = Off
Mix Signal = 0.5000
Aperture No. = 1
FIB Blanked = Yes
FIB Probe = 30KV:500 pA
FIB Imaging = SEM

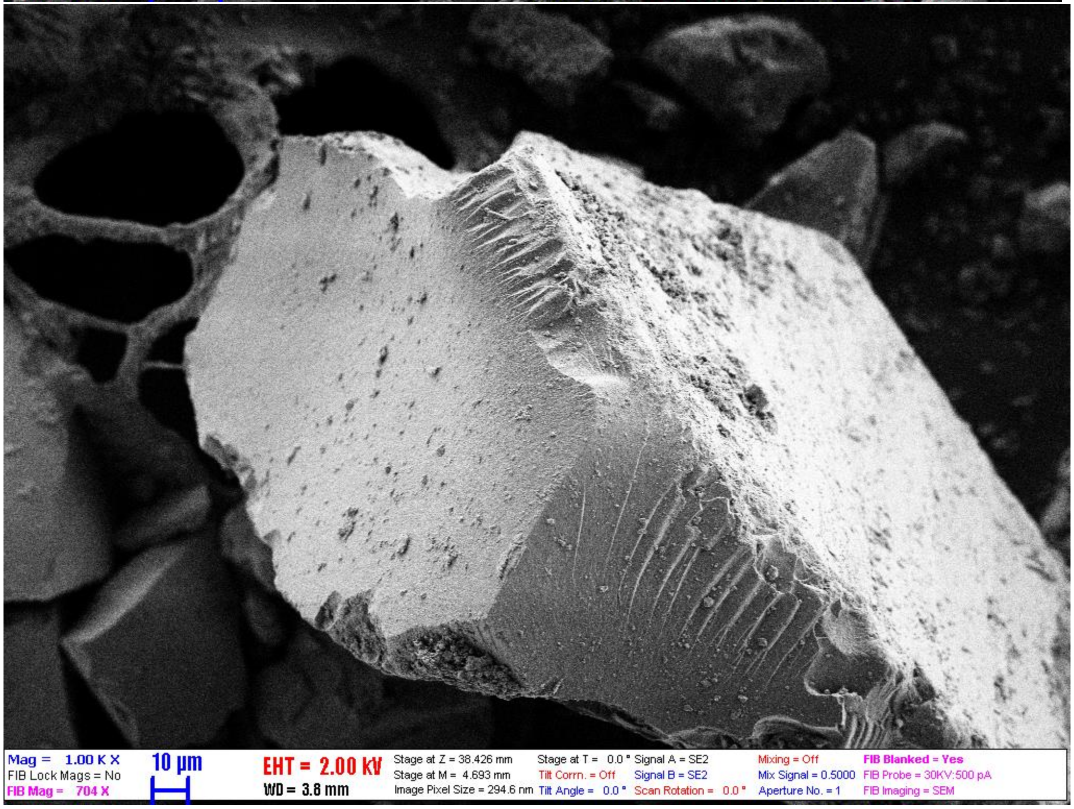
Mag = 1.00 K X
FIB Lock Mags = No
FIB Mag = 704 X
10 µm
EHT = 2.00 kV
WD = 3.8 mm
Stage at Z = 38.426 mm
Stage at M = 4.693 mm
Image Pixel Size = 294.6 nm
Stage at T = 0.0 °
Tilt Corrn. = Off
Tilt Angle = 0.0 °
Signal A = SE2
Signal B = SE2
Scan Rotation = 0.0 °
Mixing = Off
Mix Signal = 0.5000
Aperture No. = 1
FIB Blanked = Yes
FIB Probe = 30KV:500 pA
FIB Imaging = SEM

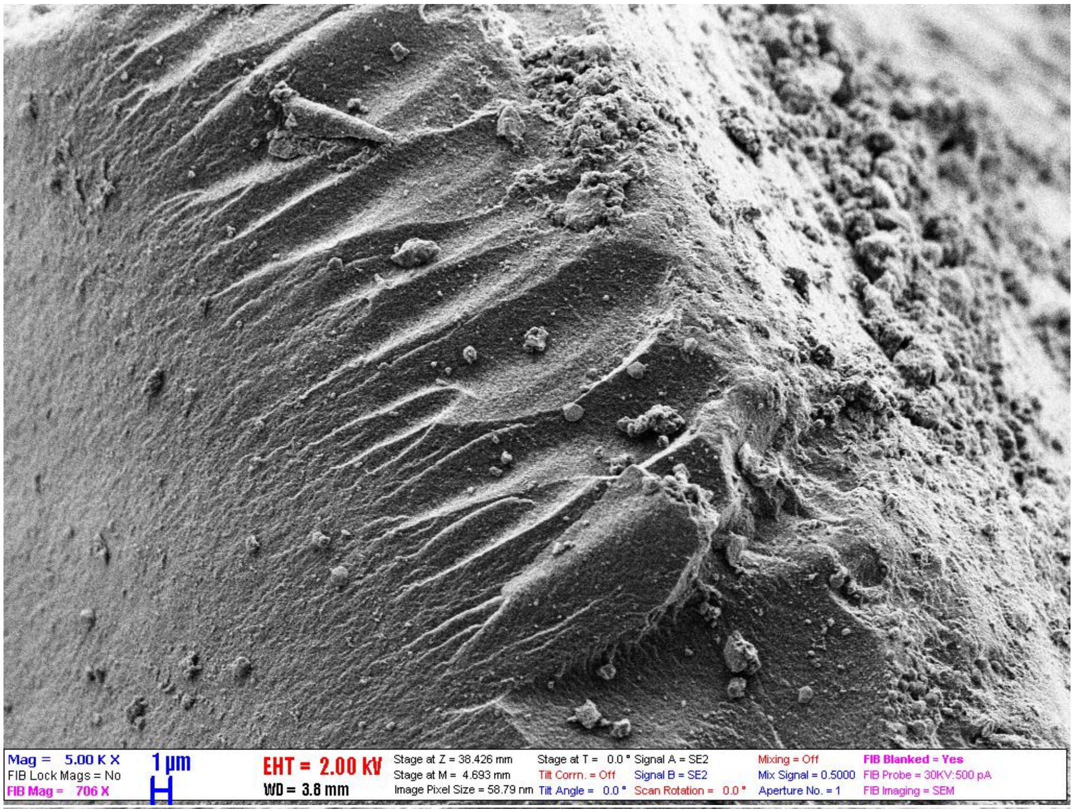
Mag = 5.00 K X
FIB Lock Mags = No
FIB Mag = 706 X
1 µm
EHT = 2.00 kV
WD = 3.8 mm
Stage at Z = 38.426 mm
Stage at M = 4.693 mm
Image Pixel Size = 58.79 nm
Stage at T = 0.0 °
Tilt Corrn. = Off
Tilt Angle = 0.0 °
Signal A = SE2
Signal B = SE2
Scan Rotation = 0.0 °
Mixing = Off
Mix Signal = 0.5000
Aperture No. = 1
FIB Blanked = Yes
FIB Probe = 30KV:500 pA
FIB Imaging = SEM

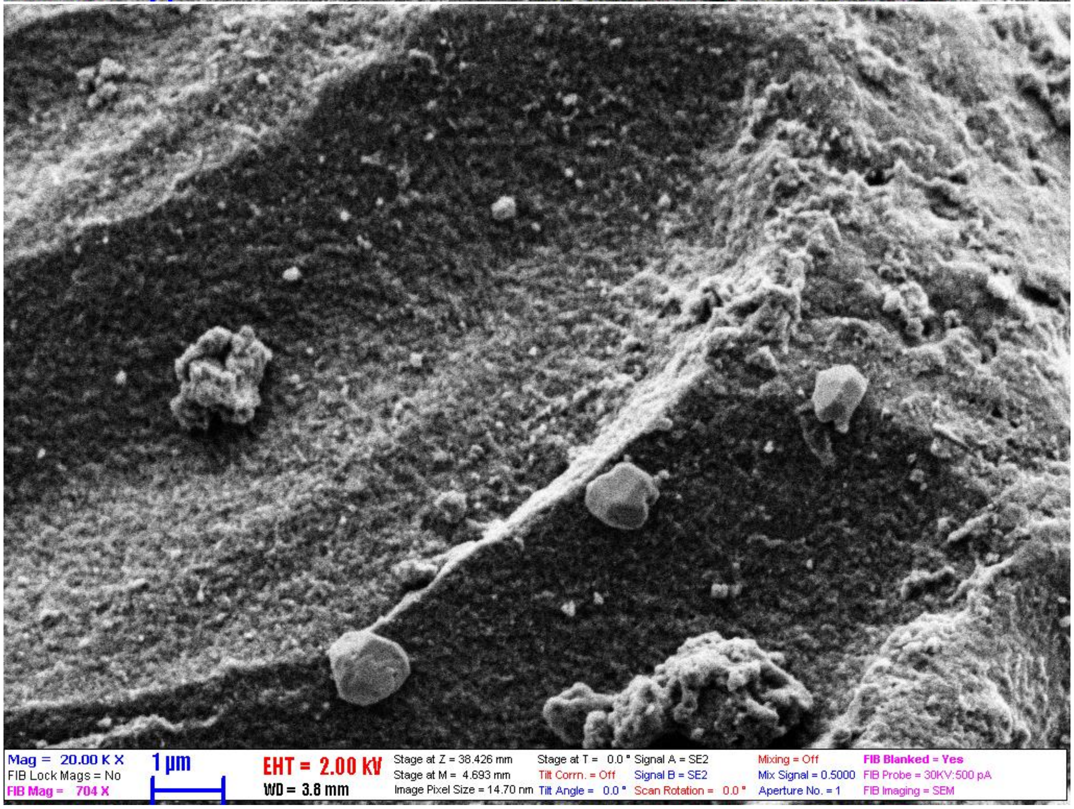
Mag = 20.00 K X
FIB Lock Mags = No
FIB Mag = 704 X
1 µm
EHT = 2.00 kV
WD = 3.8 mm
Stage at Z = 38.426 mm
Stage at M = 4.693 mm
Image Pixel Size = 14.70 nm
Stage at T = 0.0 °
Tilt Corrn. = Off
Tilt Angle = 0.0 °
Signal A = SE2
Signal B = SE2
Scan Rotation = 0.0 °
Mixing = Off
Mix Signal = 0.5000
Aperture No. = 1
FIB Blanked = Yes
FIB Probe = 30KV:500 pA
FIB Imaging = SEM

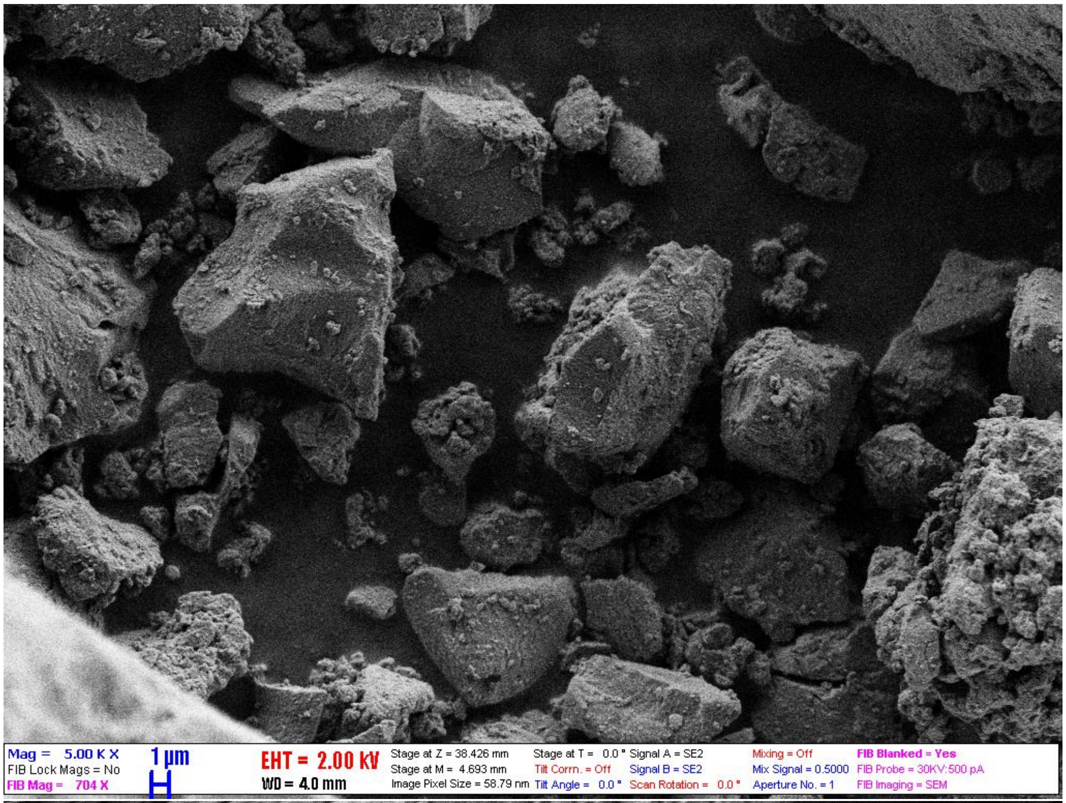
Mag = 5.00 K X
FIB Lock Mags = No
FIB Mag = 704 X
1 µm
EHT = 2.00 kV
WD = 4.0 mm
Stage at Z = 38.426 mm
Stage at M = 4.693 mm
Image Pixel Size = 58.79 nm
Stage at T = 0.0 °
Tilt Corrn. = Off
Tilt Angle = 0.0 °
Signal A = SE2
Signal B = SE2
Scan Rotation = 0.0 °
Mixing = Off
Mix Signal = 0.5000
Aperture No. = 1
FIB Blanked = Yes
FIB Probe = 30KV:500 pA
FIB Imaging = SEM

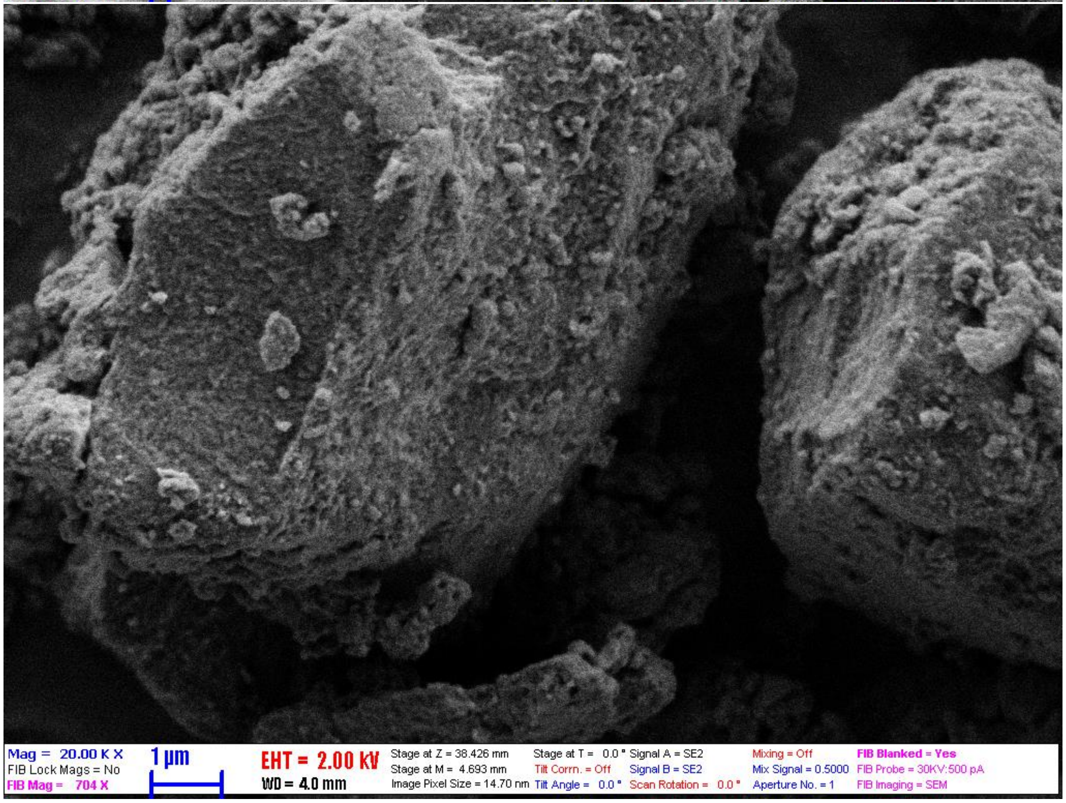
Mag = 20.00 K X
FIB Lock Mags = No
FIB Mag = 704 X
1 µm
EHT = 2.00 kV
WD = 4.0 mm
Stage at Z = 38.426 mm
Stage at M = 4.693 mm
Image Pixel Size = 14.70 nm
Stage at T = 0.0 °
Tilt Corrn. = Off
Tilt Angle = 0.0 °
Signal A = SE2
Signal B = SE2
Scan Rotation = 0.0 °
Mixing = Off
Mix Signal = 0.5000
Aperture No. = 1
FIB Blanked = Yes
FIB Probe = 30KV:500 pA
FIB Imaging = SEM

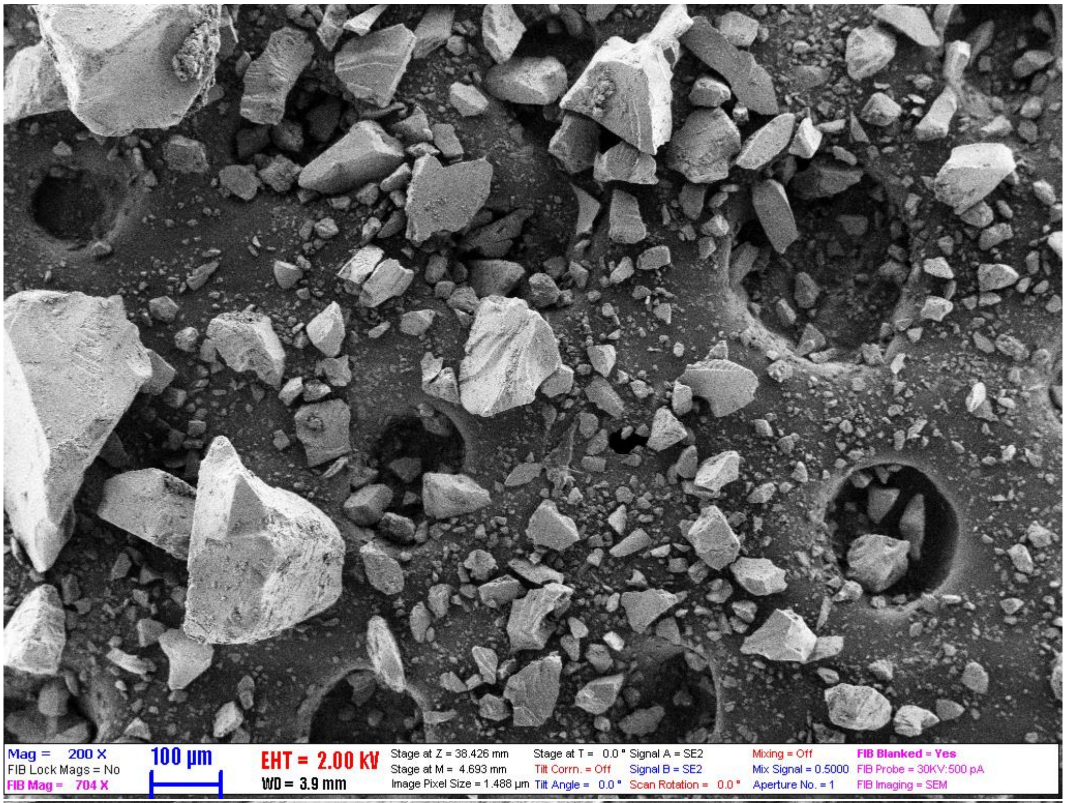
Mag = 200 X
FIB Lock Mags = No
FIB Mag = 704 X
100 µm
EHT = 2.00 kV
WD = 3.9 mm
Stage at Z = 38.426 mm
Stage at M = 4.693 mm
Image Pixel Size = 1.488 µm
Stage at T = 0.0 °
Tilt Corrn. = Off
Tilt Angle = 0.0 °
Signal A = SE2
Signal B = SE2
Scan Rotation = 0.0 °
Mixing = Off
Mix Signal = 0.5000
Aperture No. = 1
FIB Blanked = Yes
FIB Probe = 30KV:500 pA
FIB Imaging = SEM

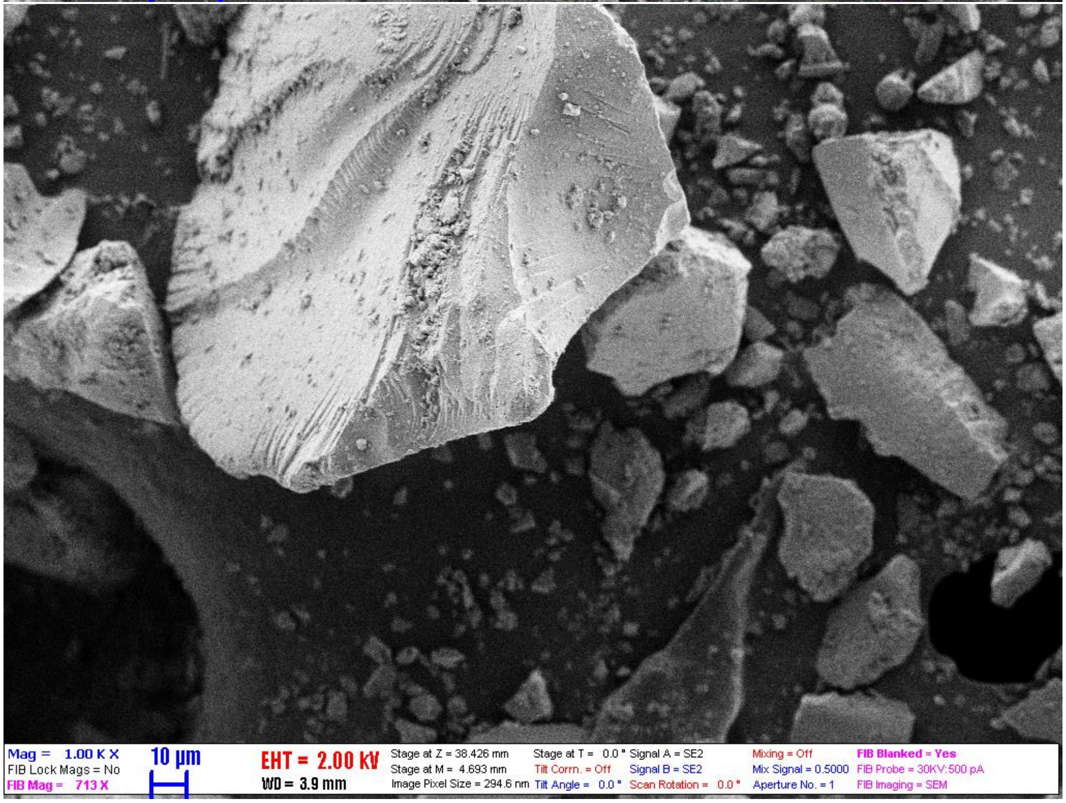
Mag = 1.00 K X
FIB Lock Mags = No
FIB Mag = 713 X
10 µm
EHT = 2.00 kV
WD = 3.9 mm
Stage at Z = 38.426 mm
Stage at M = 4.693 mm
Image Pixel Size = 294.6 nm
Stage at T = 0.0 °
Tilt Corrn. = Off
Tilt Angle = 0.0 °
Signal A = SE2
Signal B = SE2
Scan Rotation = 0.0 °
Mixing = Off
Mix Signal = 0.5000
Aperture No. = 1
FIB Blanked = Yes
FIB Probe = 30KV:500 pA
FIB Imaging = SEM

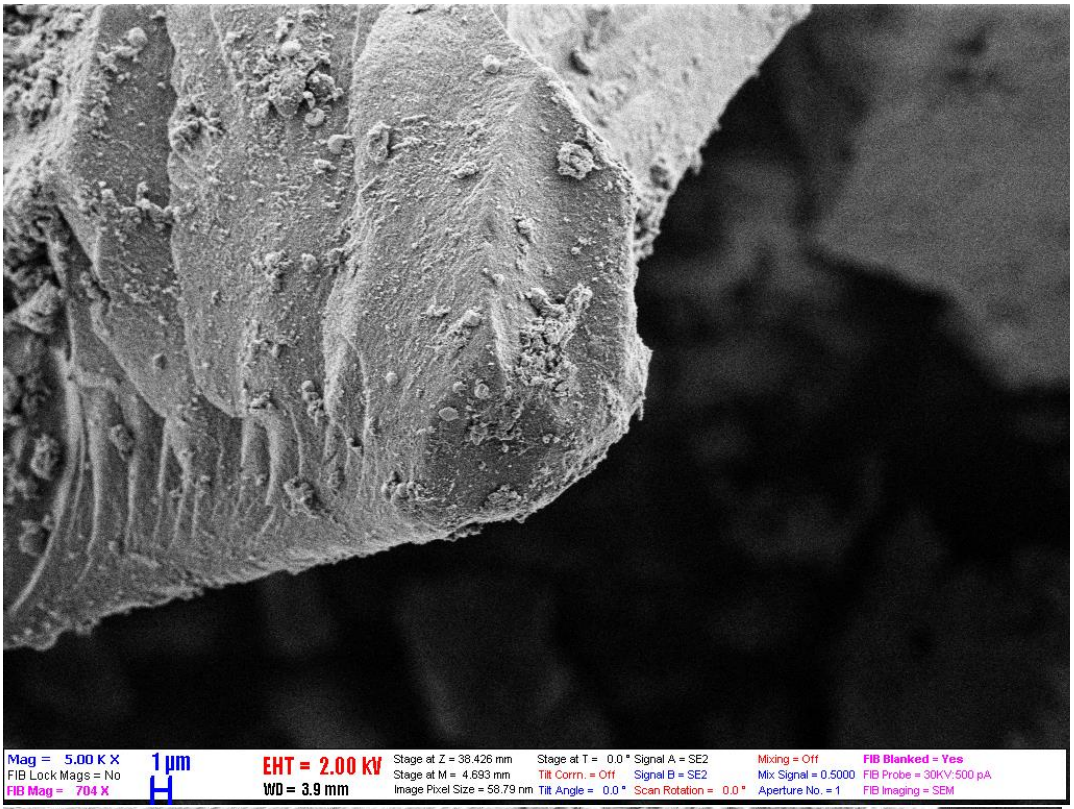
Mag = 5.00 K X
FIB Lock Mags = No
FIB Mag = 704 X
1 µm
EHT = 2.00 kV
WD = 3.9 mm
Stage at Z = 38.426 mm
Stage at M = 4.693 mm
Image Pixel Size = 58.79 nm
Stage at T = 0.0 °
Tilt Corrn. = Off
Tilt Angle = 0.0 °
Signal A = SE2
Signal B = SE2
Scan Rotation = 0.0 °
Mixing = Off
Mix Signal = 0.5000
Aperture No. = 1
FIB Blanked = Yes
FIB Probe = 30KV:500 pA
FIB Imaging = SEM

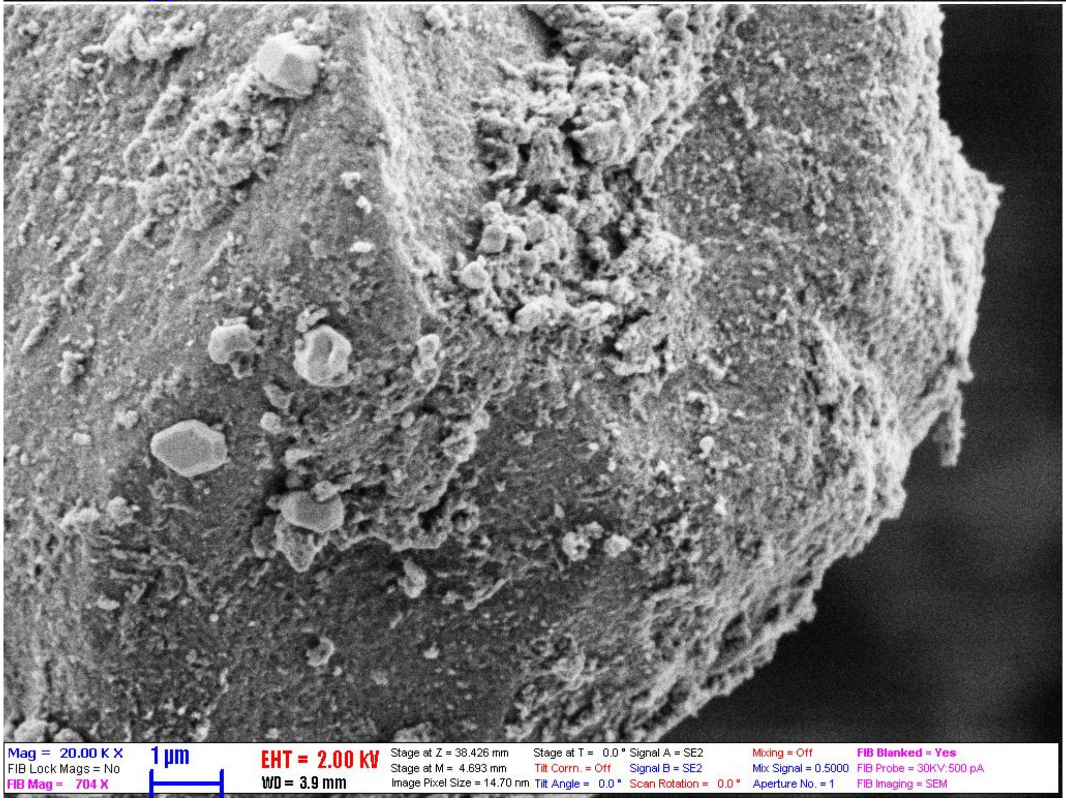
Mag = 20.00 K X
FIB Lock Mags = No
FIB Mag = 704 X
1 µm
EHT = 2.00 kV
WD = 3.9 mm
Stage at Z = 38.426 mm
Stage at M = 4.693 mm
Image Pixel Size = 14.70 nm
Stage at T = 0.0 °
Tilt Corrn. = Off
Tilt Angle = 0.0 °
Signal A = SE2
Signal B = SE2
Scan Rotation = 0.0 °
Mixing = Off
Mix Signal = 0.5000
Aperture No. = 1
FIB Blanked = Yes
FIB Probe = 30KV:500 pA
FIB Imaging = SEM

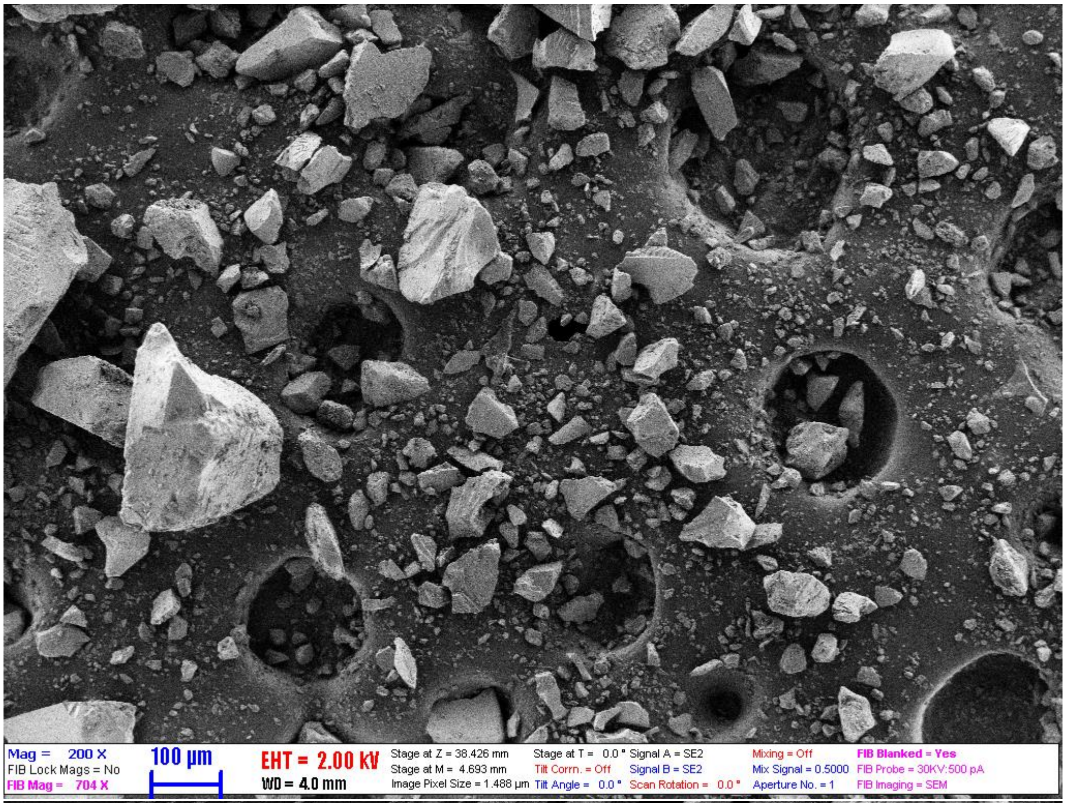
Mag = 200 X
FIB Lock Mags = No
FIB Mag = 704 X
100 µm
EHT = 2.00 kV
WD = 4.0 mm
Stage at Z = 38.426 mm
Stage at M = 4.693 mm
Image Pixel Size = 1.488 µm
Stage at T = 0.0 °
Tilt Corrn. = Off
Tilt Angle = 0.0 °
Signal A = SE2
Signal B = SE2
Scan Rotation = 0.0 °
Mixing = Off
Mix Signal = 0.5000
Aperture No. = 1
FIB Blanked = Yes
FIB Probe = 30KV:500 pA
FIB Imaging = SEM

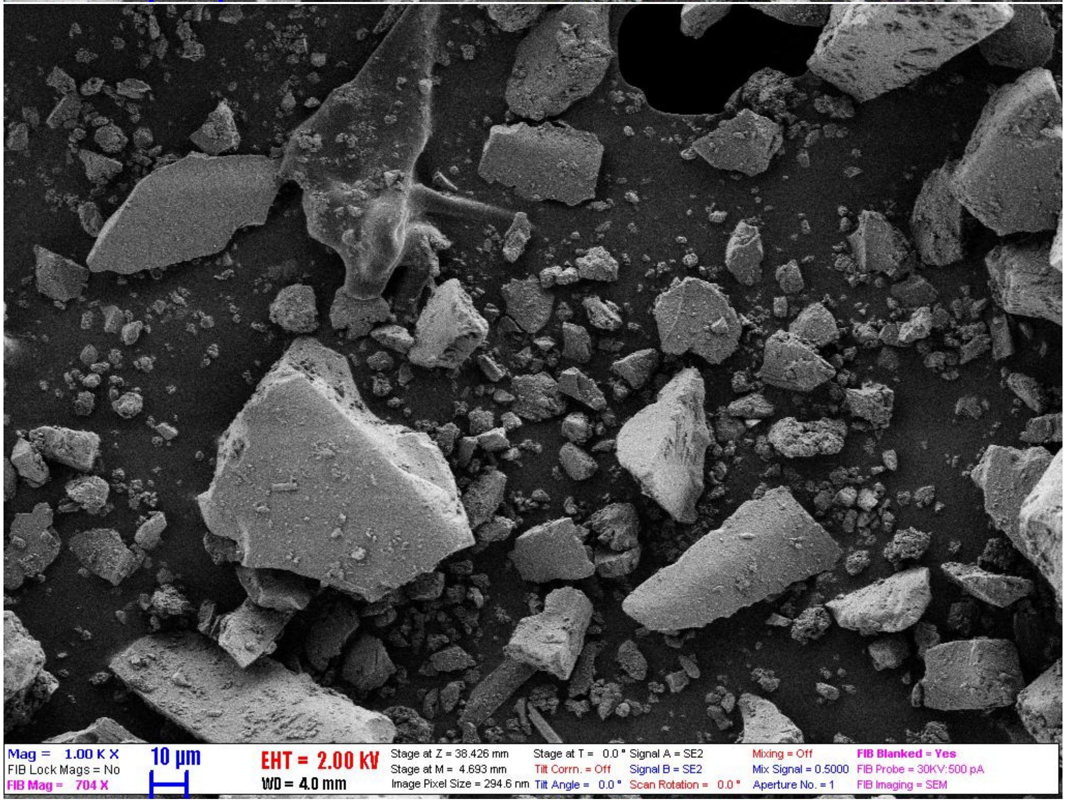
Mag = 1.00 K X
FIB Lock Mags = No
FIB Mag = 704 X
10 µm
EHT = 2.00 kV
WD = 4.0 mm
Stage at Z = 38.426 mm
Stage at M = 4.693 mm
Image Pixel Size = 294.6 nm
Stage at T = 0.0 °
Tilt Corrn. = Off
Tilt Angle = 0.0 °
Signal A = SE2
Signal B = SE2
Scan Rotation = 0.0 °
Mixing = Off
Mix Signal = 0.5000
Aperture No. = 1
FIB Blanked = Yes
FIB Probe = 30KV:500 pA
FIB Imaging = SEM

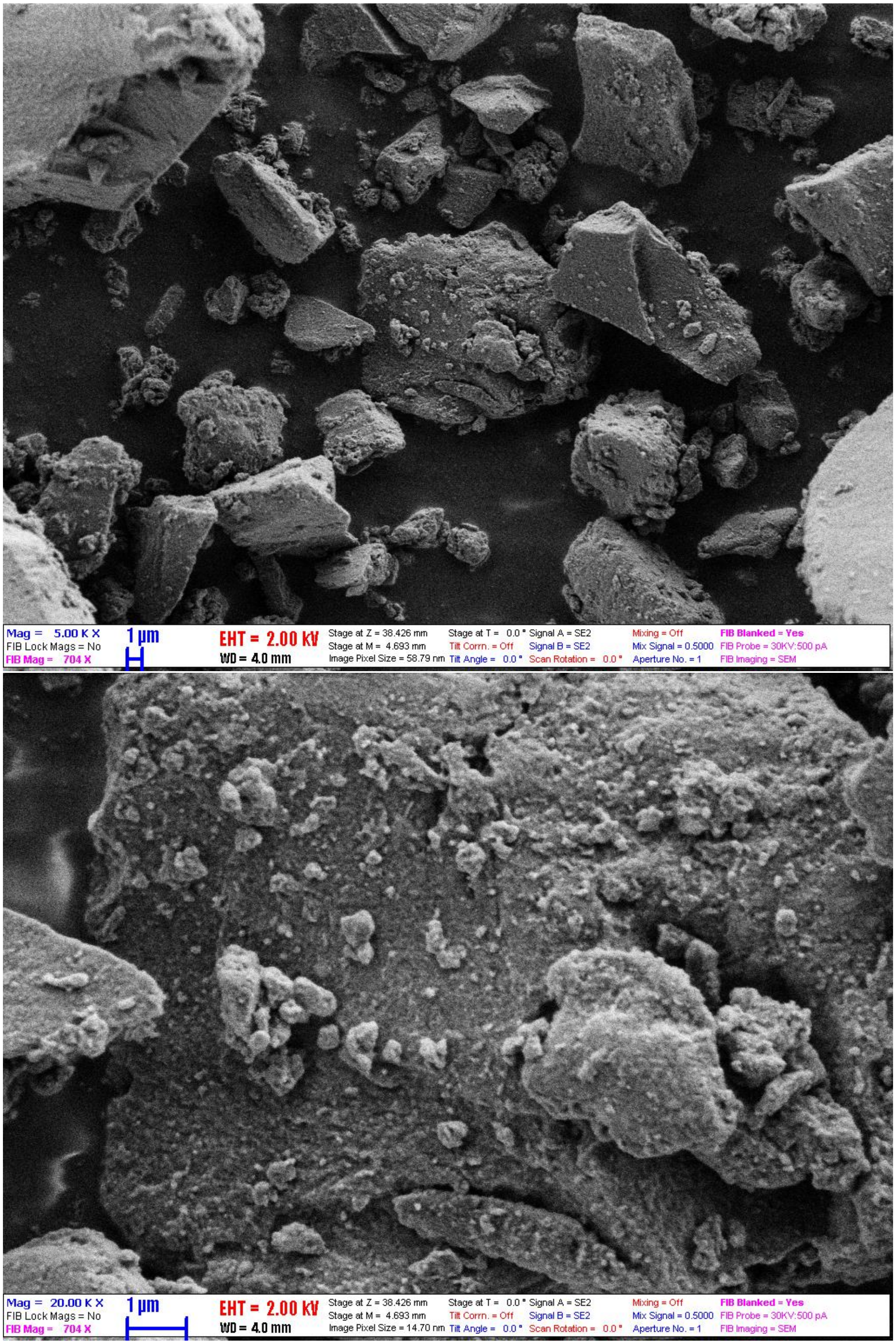

Figure S4 **IM-BF4-0.5**

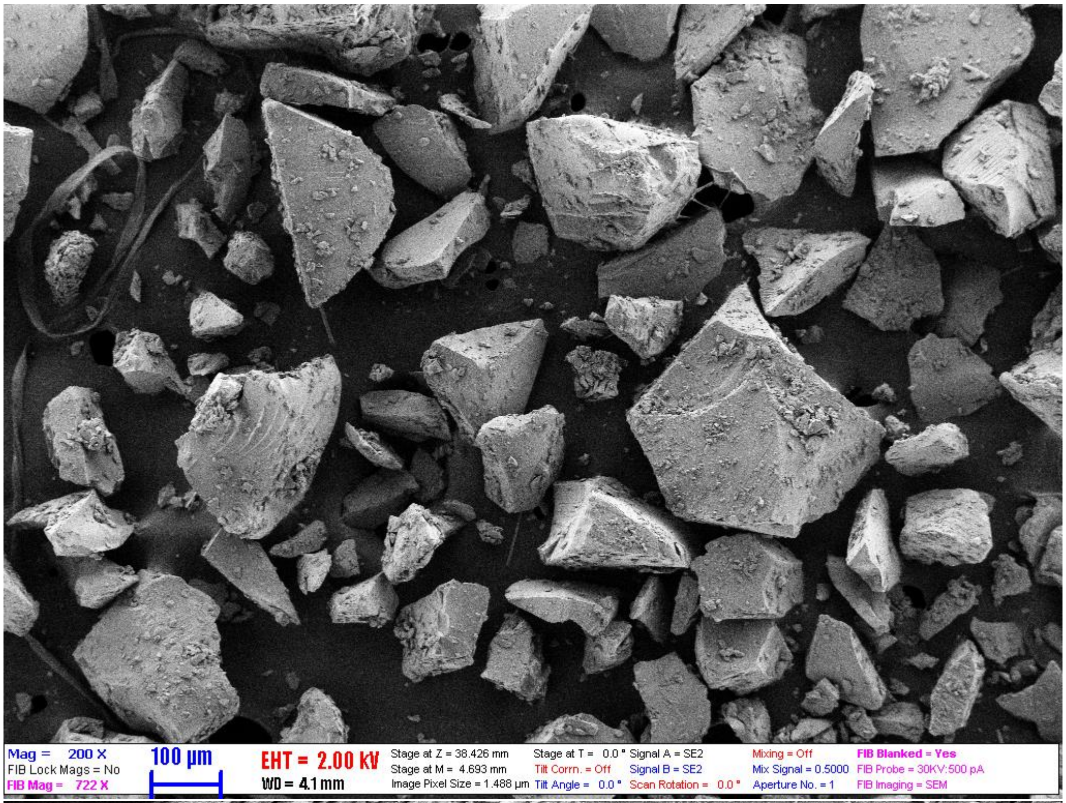
Mag = 200 X
FIB Lock Mags = No
FIB Mag = 722 X
100 µm
EHT = 2.00 kV
WD = 4.1 mm
Stage at Z = 38.426 mm
Stage at M = 4.693 mm
Image Pixel Size = 1.488 µm
Stage at T = 0.0 °
Tilt Corrn. = Off
Tilt Angle = 0.0 °
Signal A = SE2
Signal B = SE2
Scan Rotation = 0.0 °
Mixing = Off
Mix Signal = 0.5000
Aperture No. = 1
FIB Blanked = Yes
FIB Probe = 30KV:500 pA
FIB Imaging = SEM

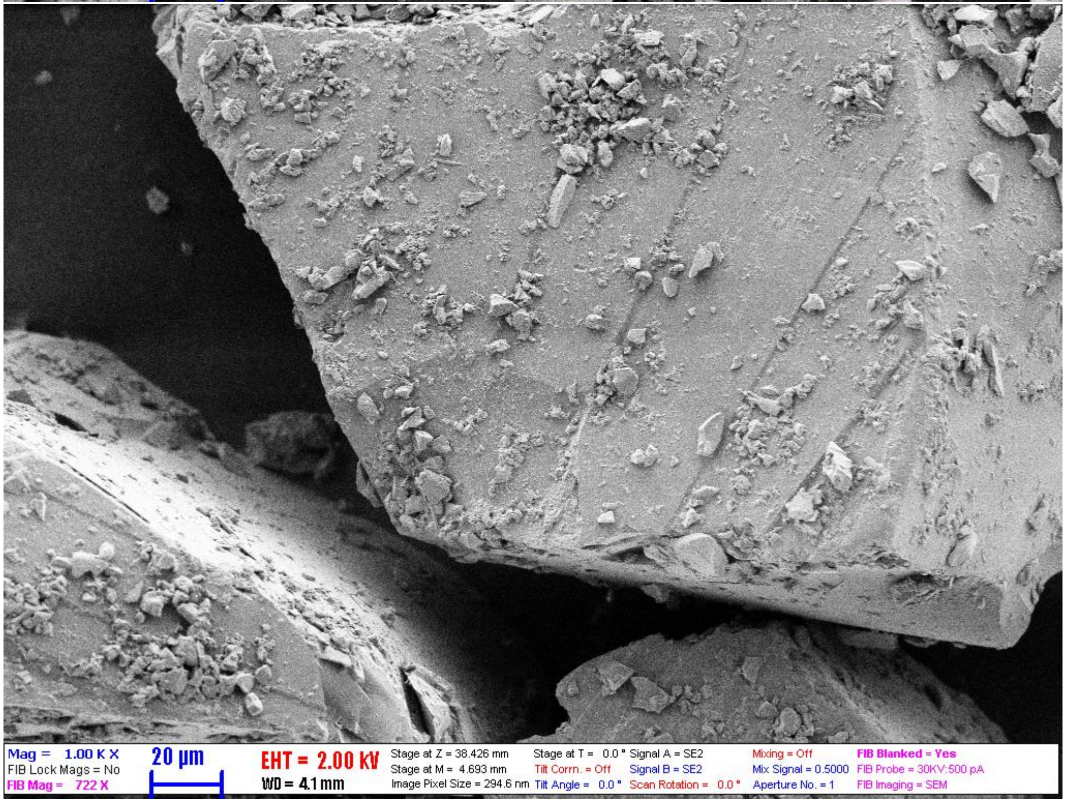
Mag = 1.00 K X
FIB Lock Mags = No
FIB Mag = 722 X
20 µm
EHT = 2.00 kV
WD = 4.1 mm
Stage at Z = 38.426 mm
Stage at M = 4.693 mm
Image Pixel Size = 294.6 nm
Stage at T = 0.0 °
Tilt Corrn. = Off
Tilt Angle = 0.0 °
Signal A = SE2
Signal B = SE2
Scan Rotation = 0.0 °
Mixing = Off
Mix Signal = 0.5000
Aperture No. = 1
FIB Blanked = Yes
FIB Probe = 30KV:500 pA
FIB Imaging = SEM

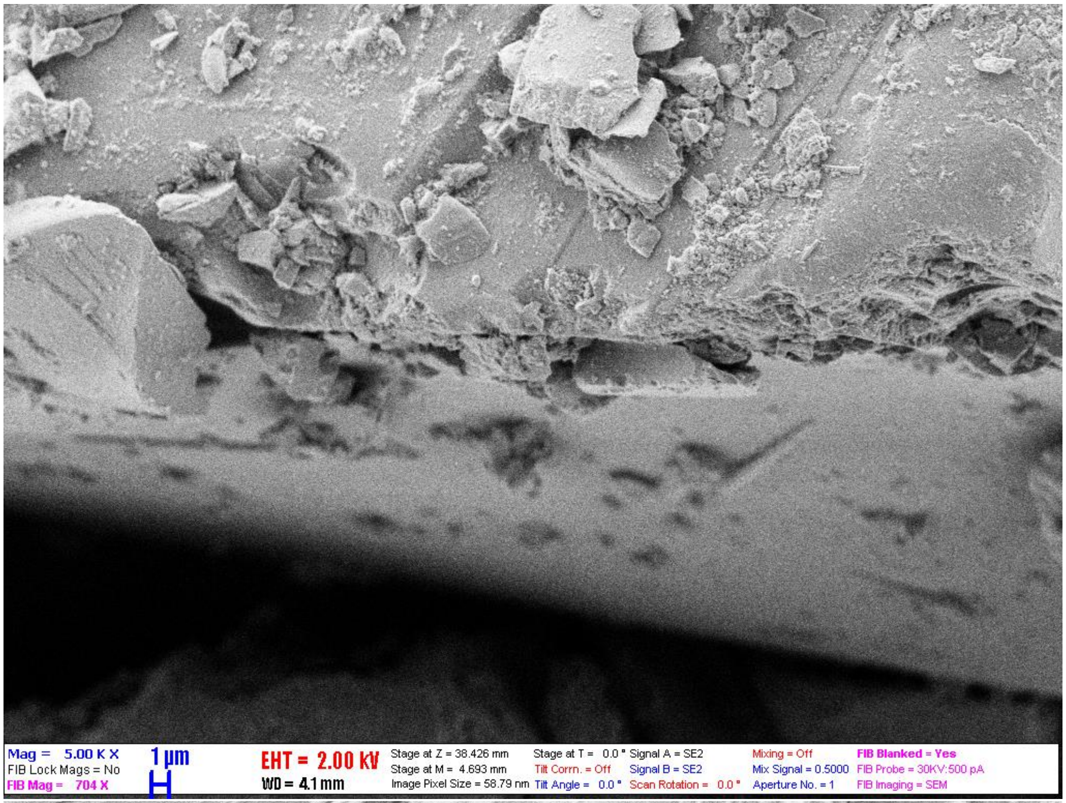
Mag = 5.00 K X
FIB Lock Mags = No
FIB Mag = 704 X
1 µm
EHT = 2.00 kV
WD = 4.1 mm
Stage at Z = 38.426 mm
Stage at M = 4.693 mm
Image Pixel Size = 58.79 nm
Stage at T = 0.0 °
Tilt Corrn. = Off
Tilt Angle = 0.0 °
Signal A = SE2
Signal B = SE2
Scan Rotation = 0.0 °
Mixing = Off
Mix Signal = 0.5000
Aperture No. = 1
FIB Blanked = Yes
FIB Probe = 30KV:500 pA
FIB Imaging = SEM

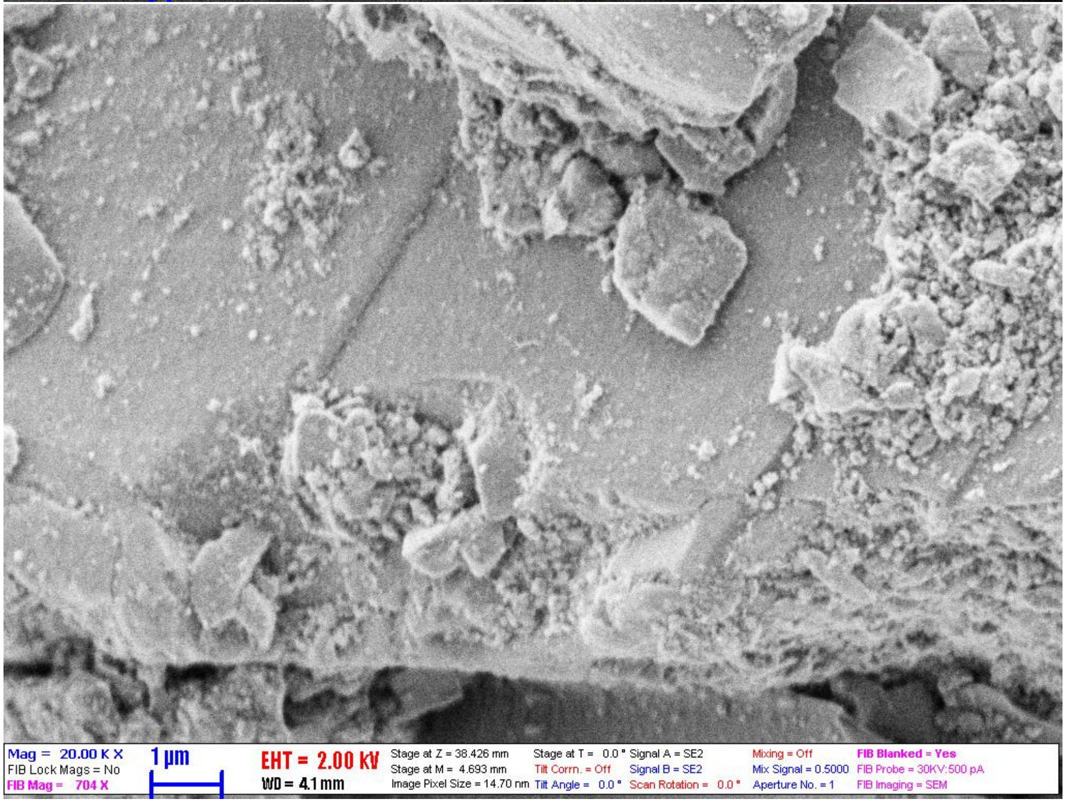
Mag = 20.00 K X
FIB Lock Mags = No
FIB Mag = 704 X
1 µm
EHT = 2.00 kV
WD = 4.1 mm
Stage at Z = 38.426 mm
Stage at M = 4.693 mm
Image Pixel Size = 14.70 nm
Stage at T = 0.0 °
Tilt Corrn. = Off
Tilt Angle = 0.0 °
Signal A = SE2
Signal B = SE2
Scan Rotation = 0.0 °
Mixing = Off
Mix Signal = 0.5000
Aperture No. = 1
FIB Blanked = Yes
FIB Probe = 30KV:500 pA
FIB Imaging = SEM

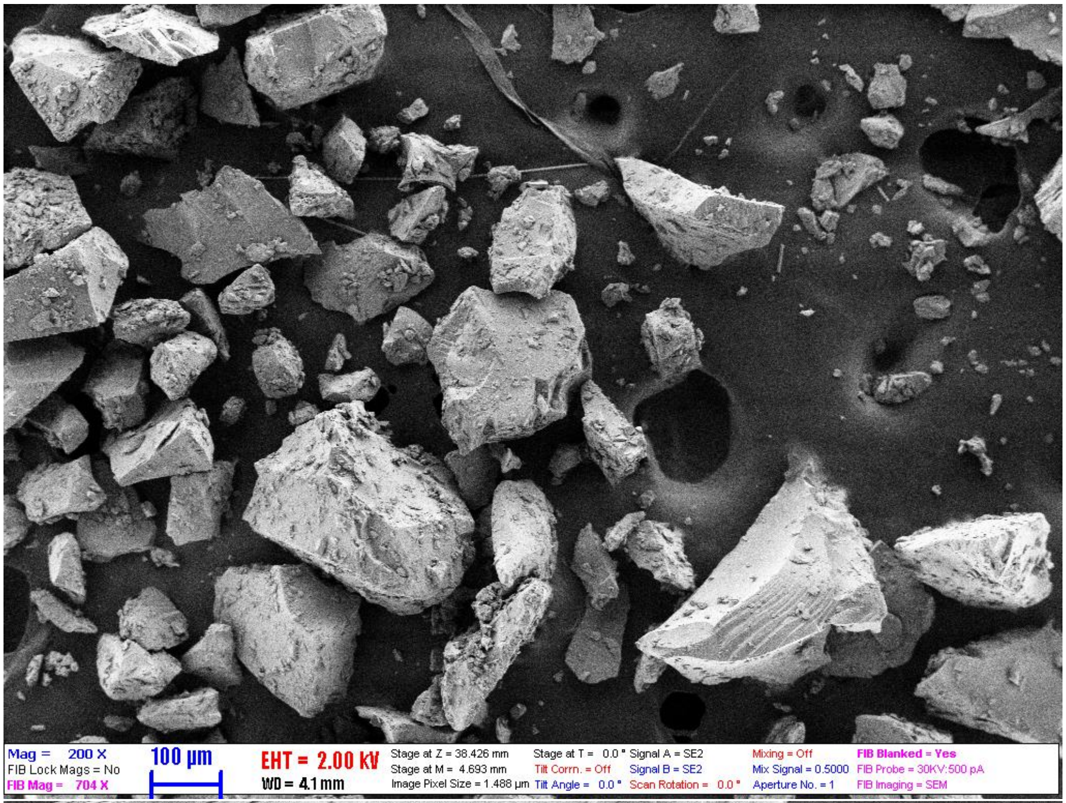
Mag = 200 X
FIB Lock Mags = No
FIB Mag = 704 X
100 µm
EHT = 2.00 kV
WD = 4.1 mm
Stage at Z = 38.426 mm
Stage at M = 4.693 mm
Image Pixel Size = 1.488 µm
Stage at T = 0.0 °
Tilt Corrn. = Off
Tilt Angle = 0.0 °
Signal A = SE2
Signal B = SE2
Scan Rotation = 0.0 °
Mixing = Off
Mix Signal = 0.5000
Aperture No. = 1
FIB Blanked = Yes
FIB Probe = 30KV:500 pA
FIB Imaging = SEM

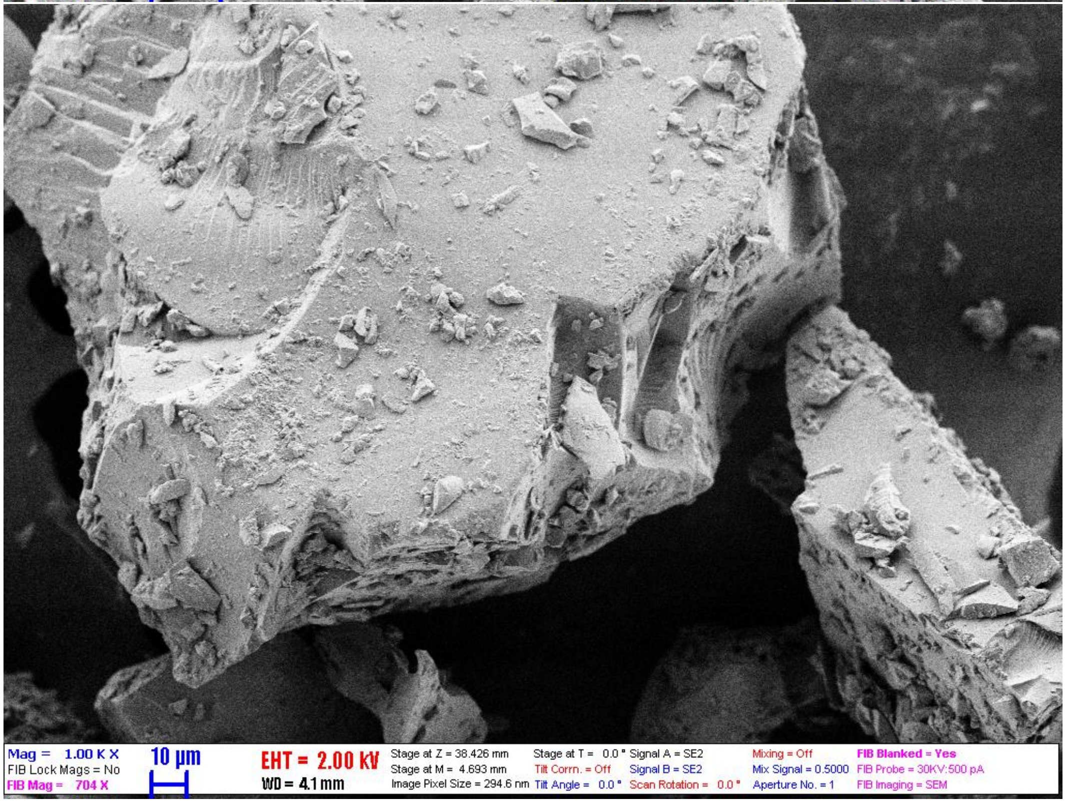
Mag = 1.00 K X
FIB Lock Mags = No
FIB Mag = 704 X
10 µm
EHT = 2.00 kV
WD = 4.1 mm
Stage at Z = 38.426 mm
Stage at M = 4.693 mm
Image Pixel Size = 294.6 nm
Stage at T = 0.0 °
Tilt Corrn. = Off
Tilt Angle = 0.0 °
Signal A = SE2
Signal B = SE2
Scan Rotation = 0.0 °
Mixing = Off
Mix Signal = 0.5000
Aperture No. = 1
FIB Blanked = Yes
FIB Probe = 30KV:500 pA
FIB Imaging = SEM

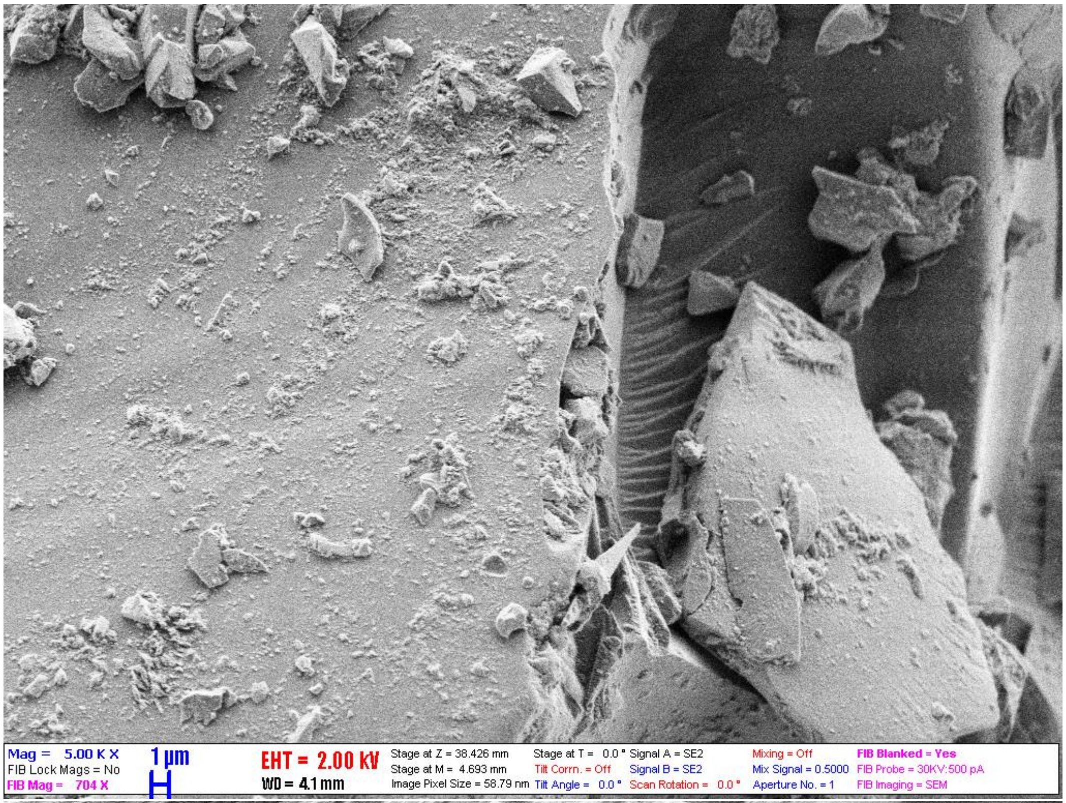
Mag = 5.00 K X
FIB Lock Mags = No
FIB Mag = 704 X
1 µm
EHT = 2.00 kV
WD = 4.1 mm
Stage at Z = 38.426 mm
Stage at M = 4.693 mm
Image Pixel Size = 58.79 nm
Stage at T = 0.0 °
Tilt Corrn. = Off
Tilt Angle = 0.0 °
Signal A = SE2
Signal B = SE2
Scan Rotation = 0.0 °
Mixing = Off
Mix Signal = 0.5000
Aperture No. = 1
FIB Blanked = Yes
FIB Probe = 30KV:500 pA
FIB Imaging = SEM

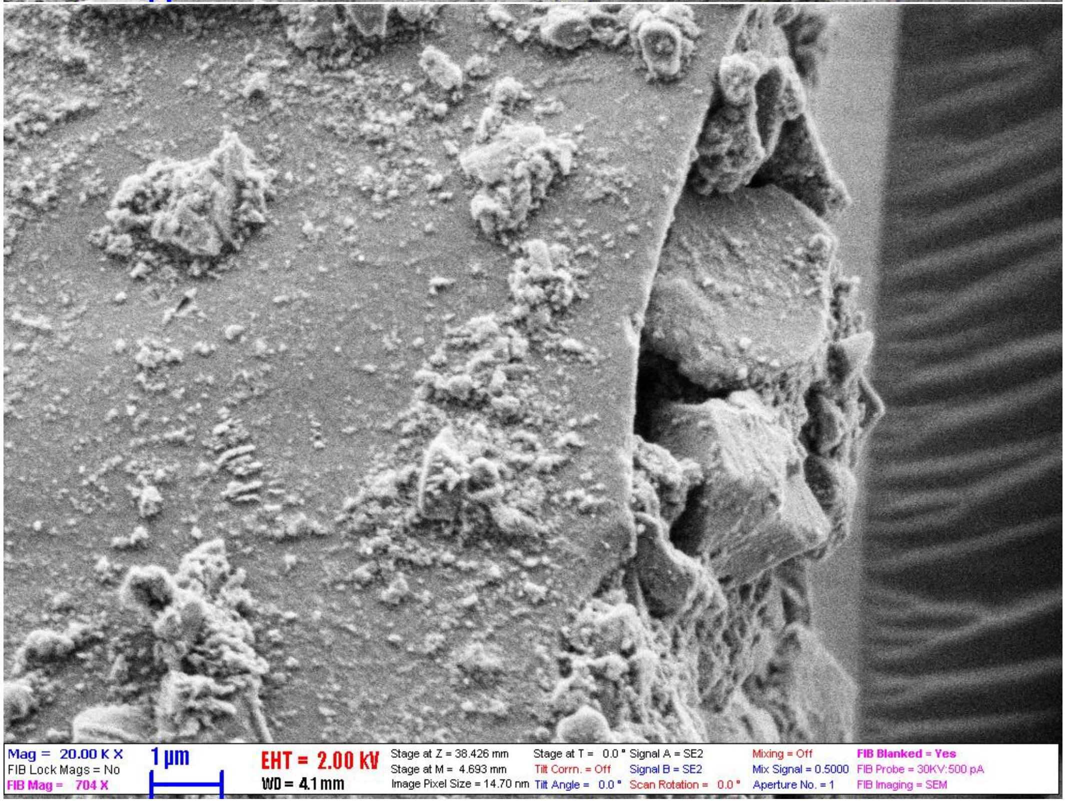
Mag = 20.00 K X
FIB Lock Mags = No
FIB Mag = 704 X
1 µm
EHT = 2.00 kV
WD = 4.1 mm
Stage at Z = 38.426 mm
Stage at M = 4.693 mm
Image Pixel Size = 14.70 nm
Stage at T = 0.0 °
Tilt Corrn. = Off
Tilt Angle = 0.0 °
Signal A = SE2
Signal B = SE2
Scan Rotation = 0.0 °
Mixing = Off
Mix Signal = 0.5000
Aperture No. = 1
FIB Blanked = Yes
FIB Probe = 30KV:500 pA
FIB Imaging = SEM

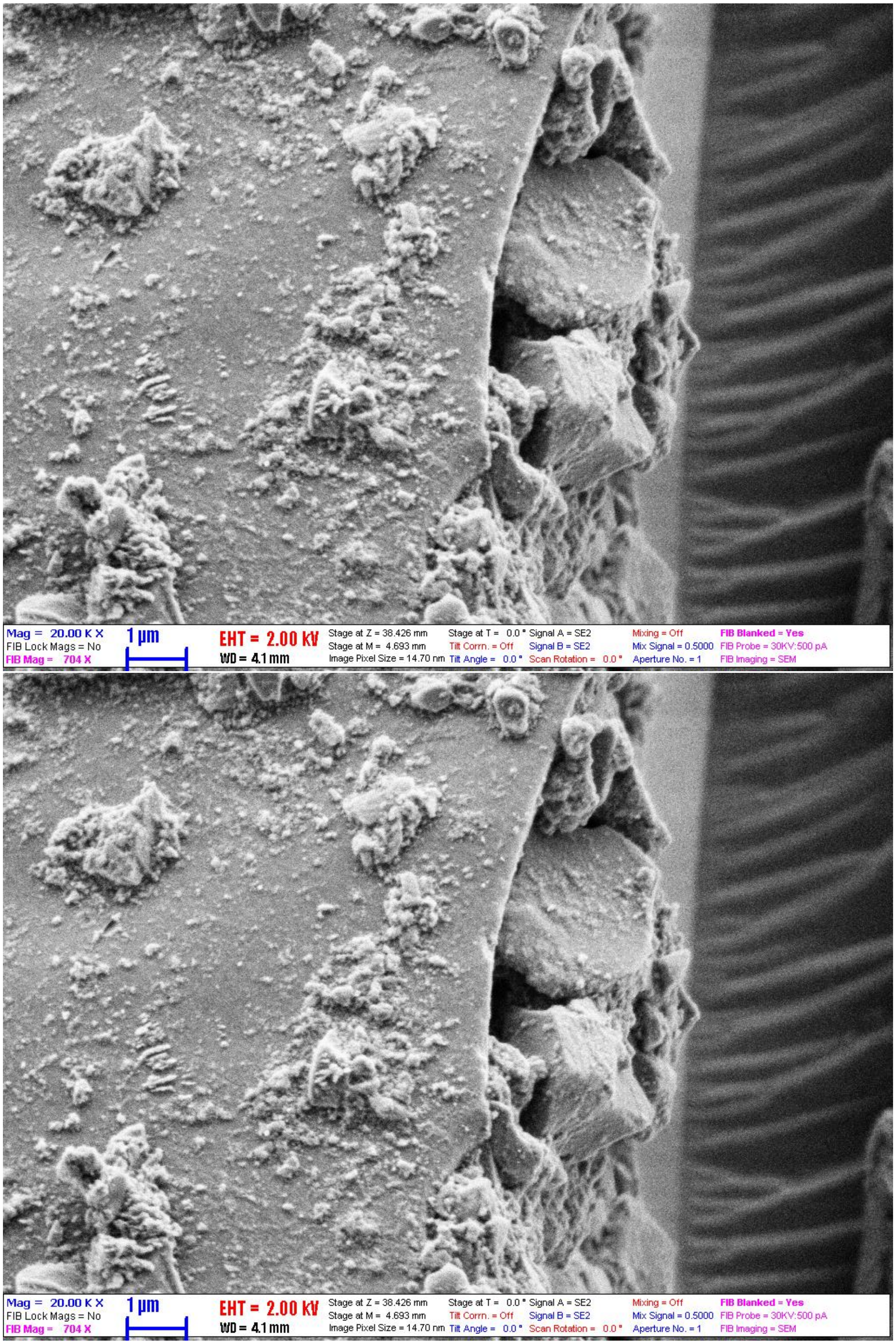

Figure S5 **IM-Cl-0.1**

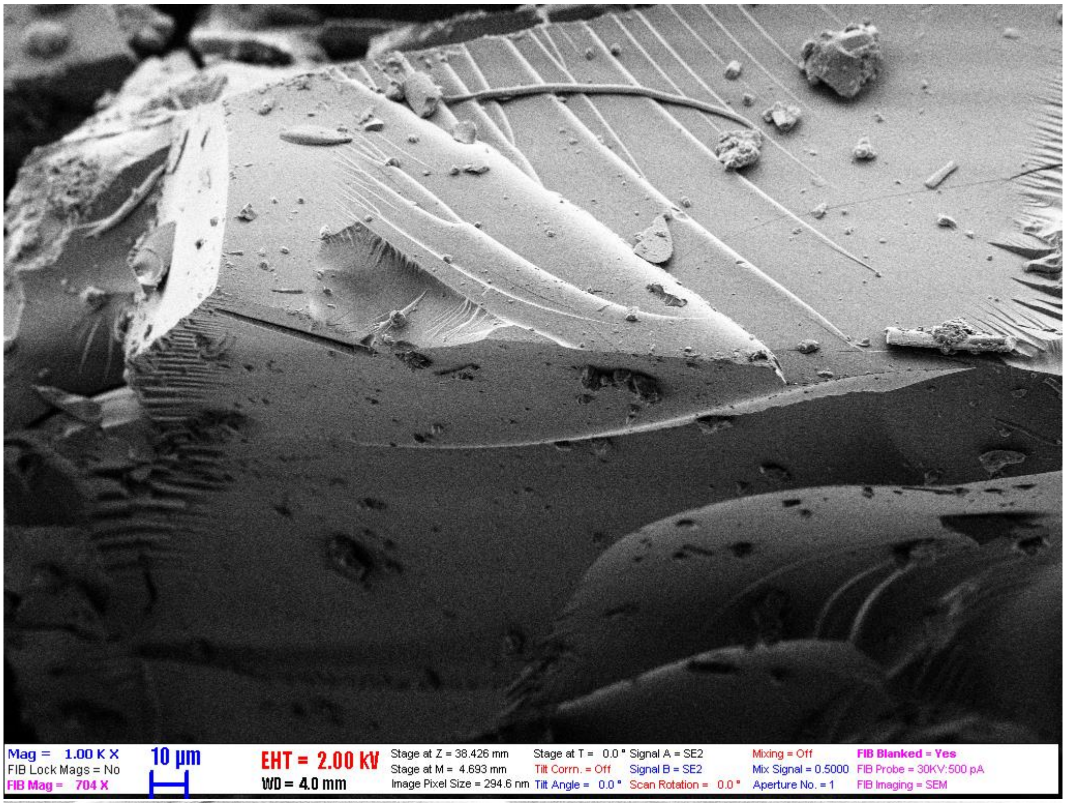
Mag = 1.00 K X
FIB Lock Mags = No
FIB Mag = 704 X
10 µm
EHT = 2.00 kV
WD = 4.0 mm
Stage at Z = 38.426 mm
Stage at M = 4.693 mm
Image Pixel Size = 294.6 nm
Stage at T = 0.0 °
Tilt Corrn. = Off
Tilt Angle = 0.0 °
Signal A = SE2
Signal B = SE2
Scan Rotation = 0.0 °
Mixing = Off
Mix Signal = 0.5000
Aperture No. = 1
FIB Blanked = Yes
FIB Probe = 30KV:500 pA
FIB Imaging = SEM

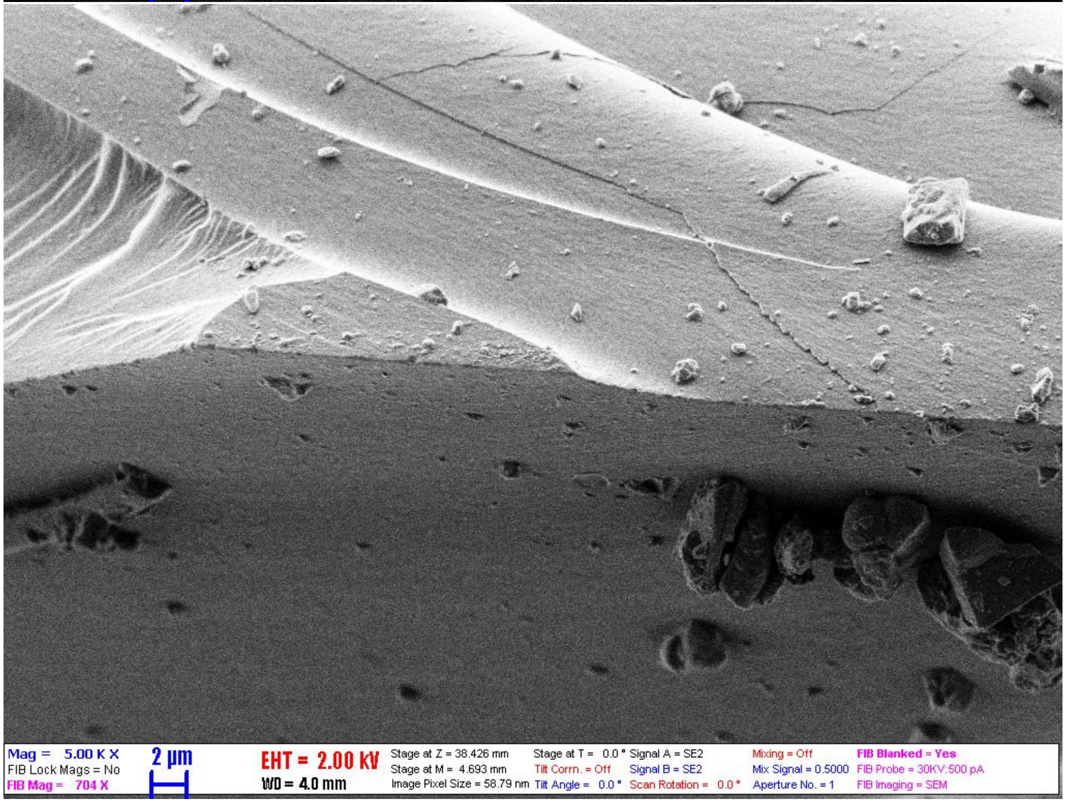
Mag = 5.00 K X
FIB Lock Mags = No
FIB Mag = 704 X
2 µm
EHT = 2.00 kV
WD = 4.0 mm
Stage at Z = 38.426 mm
Stage at M = 4.693 mm
Image Pixel Size = 58.79 nm
Stage at T = 0.0 °
Tilt Corrn. = Off
Tilt Angle = 0.0 °
Signal A = SE2
Signal B = SE2
Scan Rotation = 0.0 °
Mixing = Off
Mix Signal = 0.5000
Aperture No. = 1
FIB Blanked = Yes
FIB Probe = 30KV:500 pA
FIB Imaging = SEM

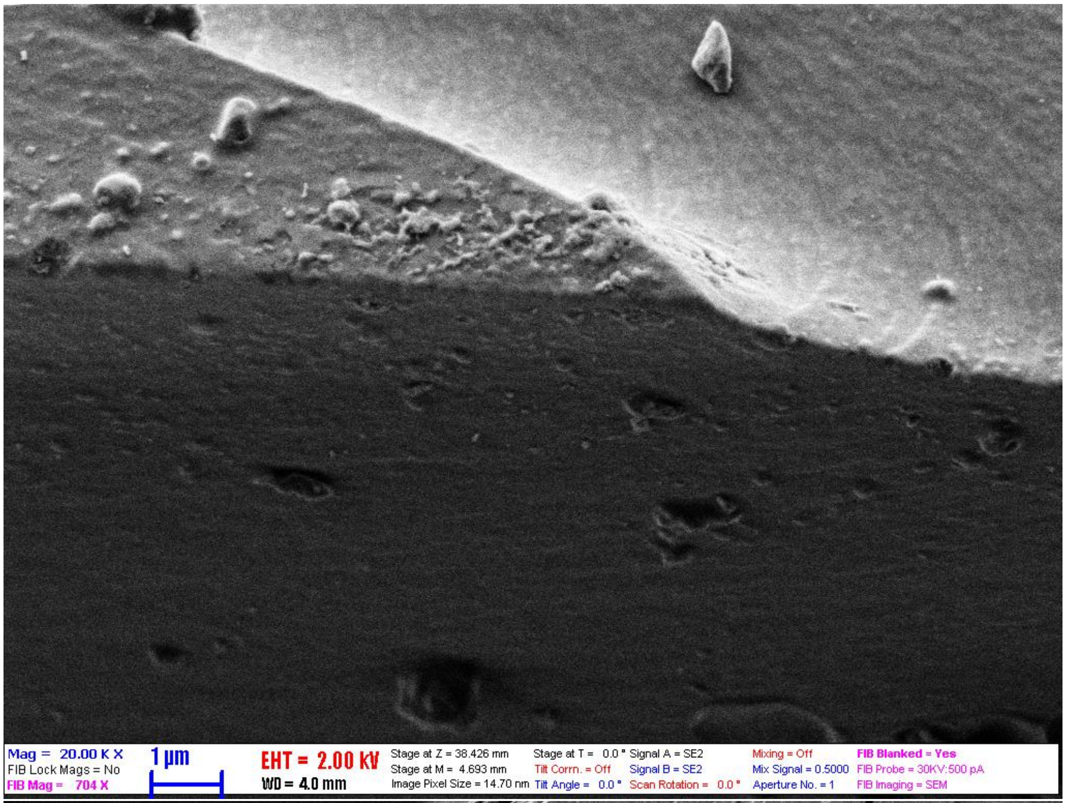
Mag = 20.00 K X
FIB Lock Mags = No
FIB Mag = 704 X
1 µm
EHT = 2.00 kV
WD = 4.0 mm
Stage at Z = 38.426 mm
Stage at M = 4.693 mm
Image Pixel Size = 14.70 nm
Stage at T = 0.0 °
Tilt Corrn. = Off
Tilt Angle = 0.0 °
Signal A = SE2
Signal B = SE2
Scan Rotation = 0.0 °
Mixing = Off
Mix Signal = 0.5000
Aperture No. = 1
FIB Blanked = Yes
FIB Probe = 30KV:500 pA
FIB Imaging = SEM

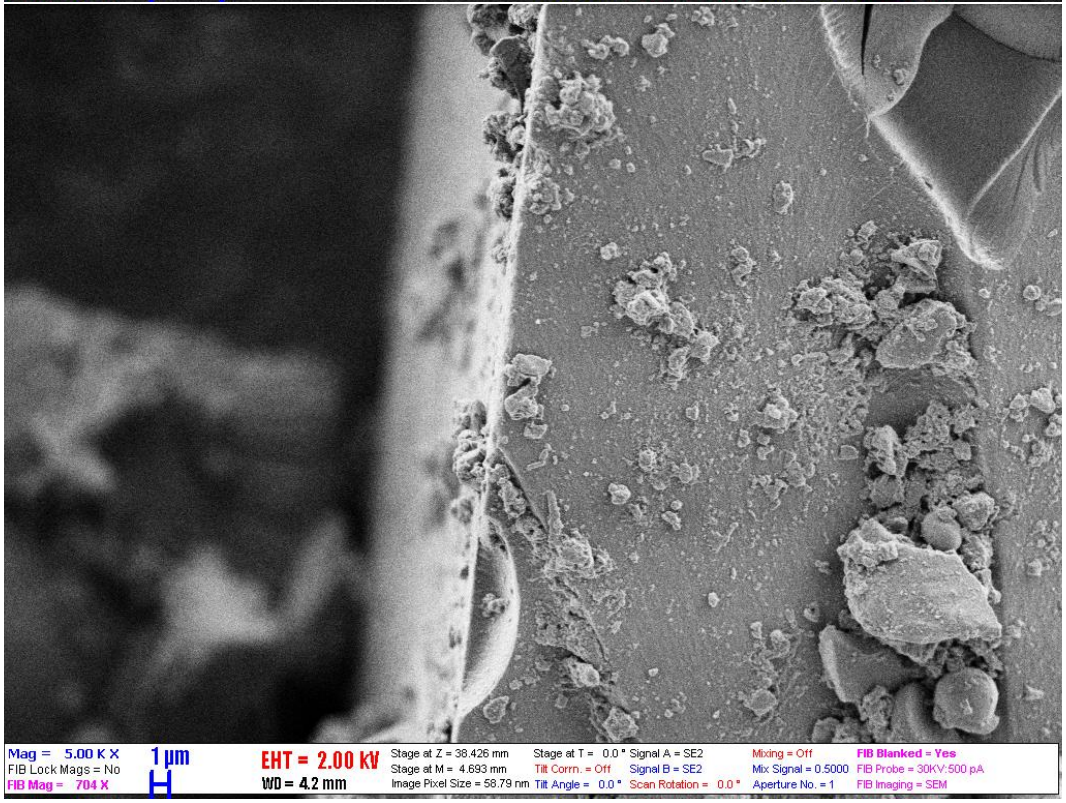
Mag = 5.00 K X
FIB Lock Mags = No
FIB Mag = 704 X
1 µm
EHT = 2.00 kV
WD = 4.2 mm
Stage at Z = 38.426 mm
Stage at M = 4.693 mm
Image Pixel Size = 58.79 nm
Stage at T = 0.0 °
Tilt Corrn. = Off
Tilt Angle = 0.0 °
Signal A = SE2
Signal B = SE2
Scan Rotation = 0.0 °
Mixing = Off
Mix Signal = 0.5000
Aperture No. = 1
FIB Blanked = Yes
FIB Probe = 30KV:500 pA
FIB Imaging = SEM

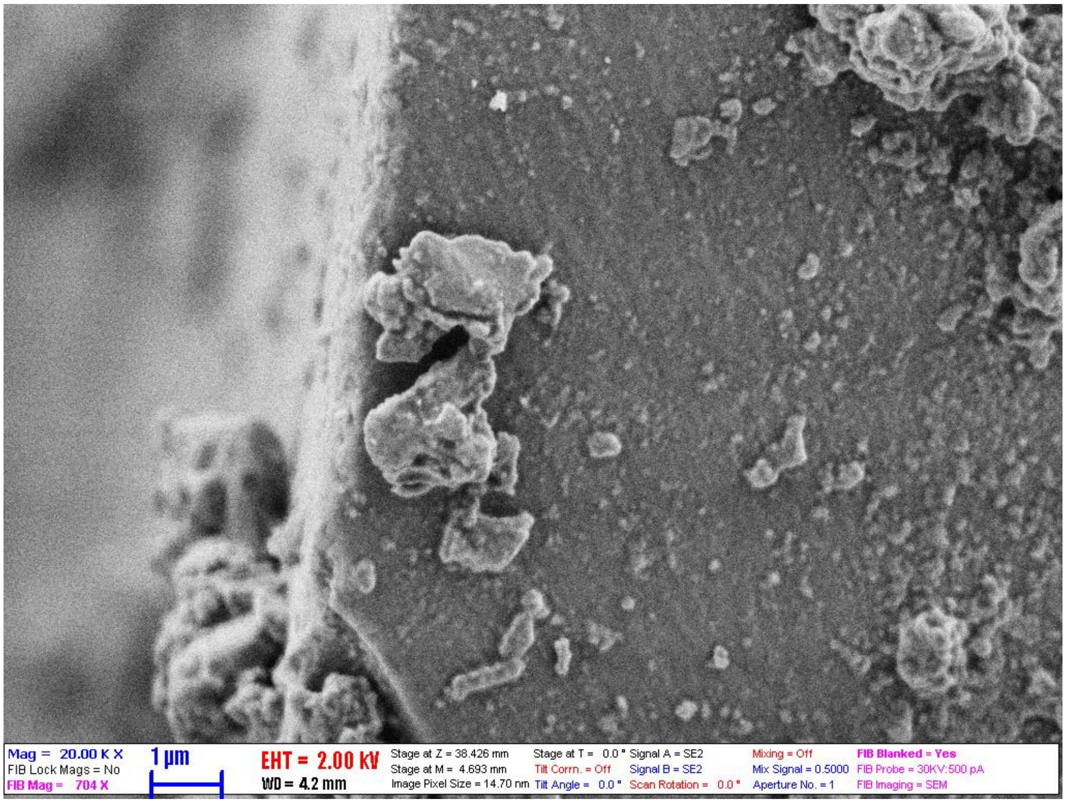

Figure S6 **IM-Cl-0.3**

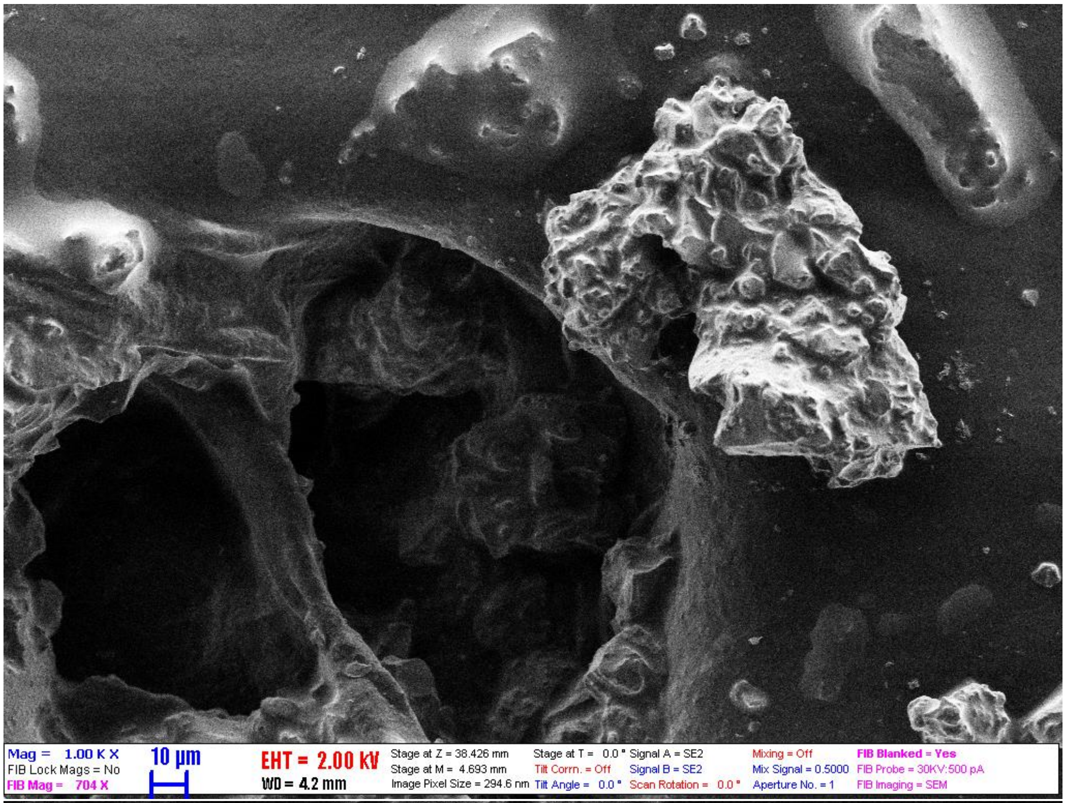
Mag = 1.00 K X
FIB Lock Mags = No
FIB Mag = 704 X
10 µm
EHT = 2.00 kV
WD = 4.2 mm
Stage at Z = 38.426 mm
Stage at M = 4.693 mm
Image Pixel Size = 294.6 nm
Stage at T = 0.0 °
Tilt Corrn. = Off
Tilt Angle = 0.0 °
Signal A = SE2
Signal B = SE2
Scan Rotation = 0.0 °
Mixing = Off
Mix Signal = 0.5000
Aperture No. = 1
FIB Blanked = Yes
FIB Probe = 30KV:500 pA
FIB Imaging = SEM

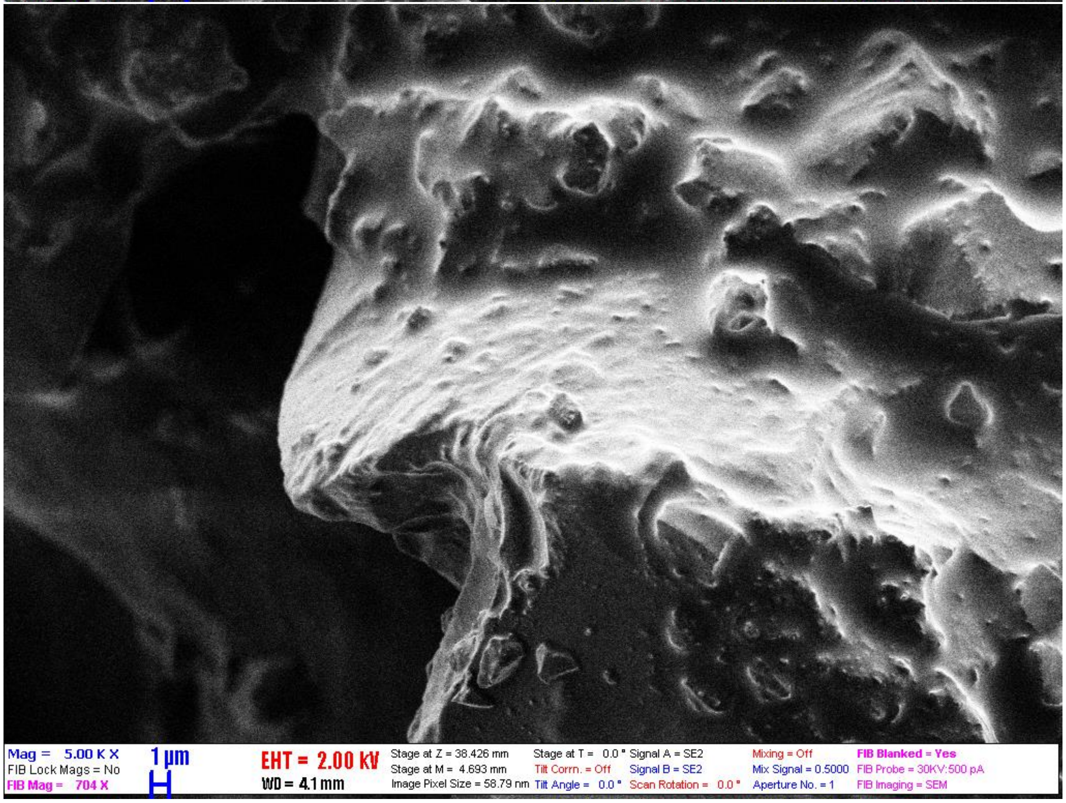
Mag = 5.00 K X
FIB Lock Mags = No
FIB Mag = 704 X
1 µm
EHT = 2.00 kV
WD = 4.1 mm
Stage at Z = 38.426 mm
Stage at M = 4.693 mm
Image Pixel Size = 58.79 nm
Stage at T = 0.0 °
Tilt Corrn. = Off
Tilt Angle = 0.0 °
Signal A = SE2
Signal B = SE2
Scan Rotation = 0.0 °
Mixing = Off
Mix Signal = 0.5000
Aperture No. = 1
FIB Blanked = Yes
FIB Probe = 30KV:500 pA
FIB Imaging = SEM

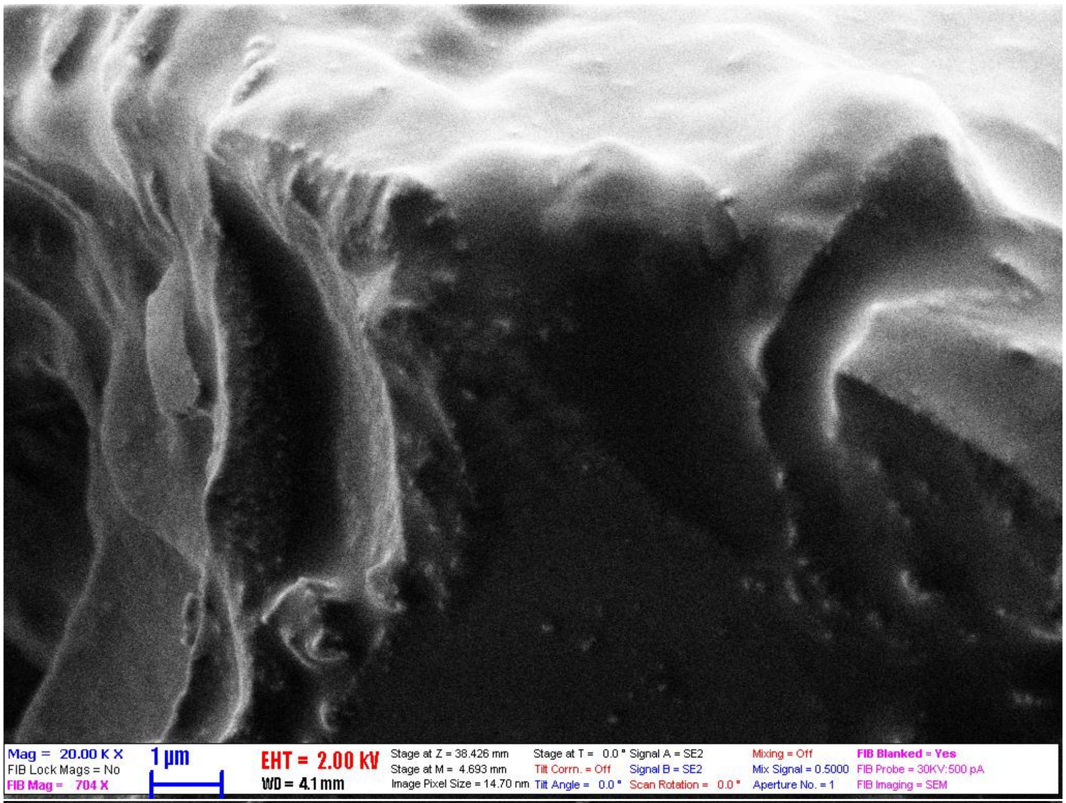
Mag = 20.00 K X
FIB Lock Mags = No
FIB Mag = 704 X
1 µm
EHT = 2.00 kV
WD = 4.1 mm
Stage at Z = 38.426 mm
Stage at M = 4.693 mm
Image Pixel Size = 14.70 nm
Stage at T = 0.0 °
Tilt Corrn. = Off
Tilt Angle = 0.0 °
Signal A = SE2
Signal B = SE2
Scan Rotation = 0.0 °
Mixing = Off
Mix Signal = 0.5000
Aperture No. = 1
FIB Blanked = Yes
FIB Probe = 30KV:500 pA
FIB Imaging = SEM

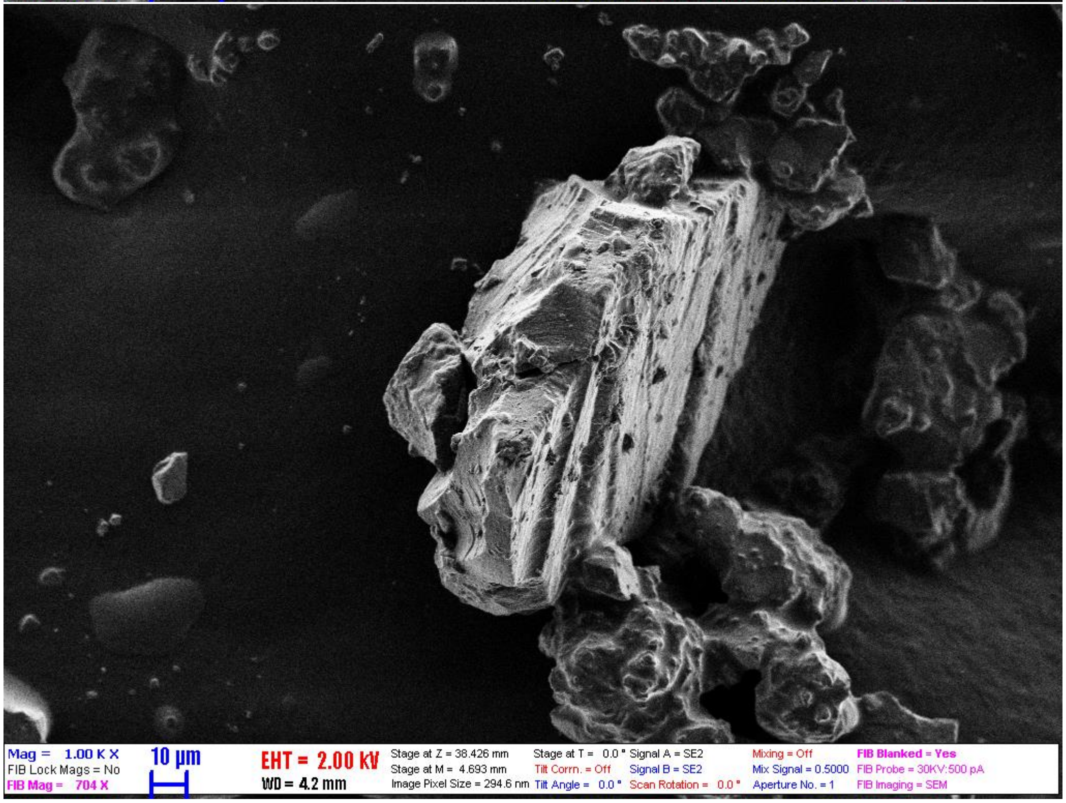
Mag = 1.00 K X
FIB Lock Mags = No
FIB Mag = 704 X
10 µm
EHT = 2.00 kV
WD = 4.2 mm
Stage at Z = 38.426 mm
Stage at M = 4.693 mm
Image Pixel Size = 294.6 nm
Stage at T = 0.0 °
Tilt Corrn. = Off
Tilt Angle = 0.0 °
Signal A = SE2
Signal B = SE2
Scan Rotation = 0.0 °
Mixing = Off
Mix Signal = 0.5000
Aperture No. = 1
FIB Blanked = Yes
FIB Probe = 30KV:500 pA
FIB Imaging = SEM

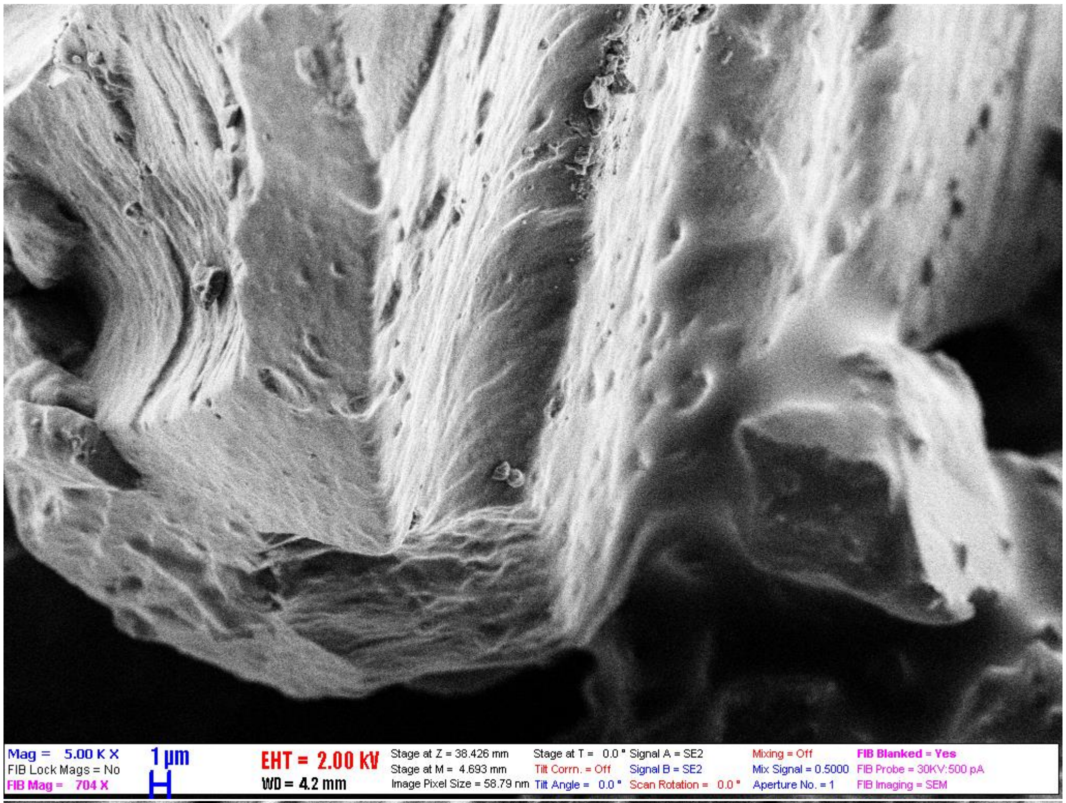
Mag = 5.00 K X
FIB Lock Mags = No
FIB Mag = 704 X
1 µm
EHT = 2.00 kV
WD = 4.2 mm
Stage at Z = 38.426 mm
Stage at M = 4.693 mm
Image Pixel Size = 58.79 nm
Stage at T = 0.0 °
Tilt Corrn. = Off
Tilt Angle = 0.0 °
Signal A = SE2
Signal B = SE2
Scan Rotation = 0.0 °
Mixing = Off
Mix Signal = 0.5000
Aperture No. = 1
FIB Blanked = Yes
FIB Probe = 30KV:500 pA
FIB Imaging = SEM

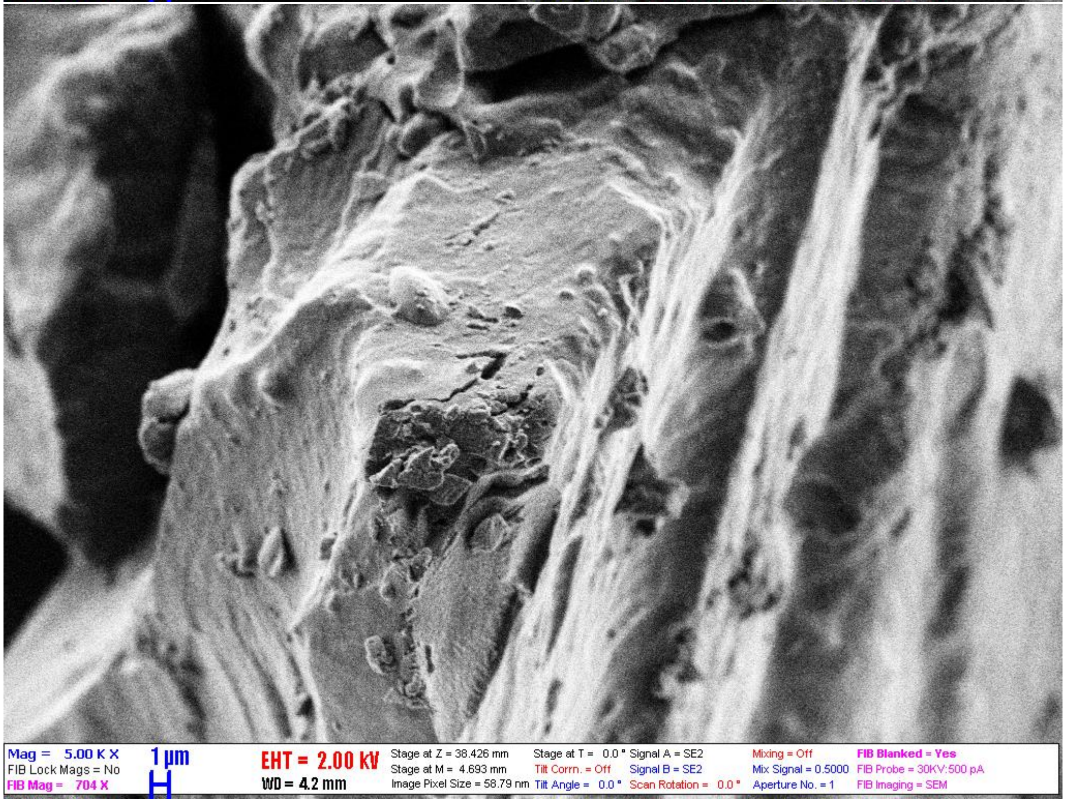
Mag = 5.00 K X
FIB Lock Mags = No
FIB Mag = 704 X
1 µm
EHT = 2.00 kV
WD = 4.2 mm
Stage at Z = 38.426 mm
Stage at M = 4.693 mm
Image Pixel Size = 58.79 nm
Stage at T = 0.0 °
Tilt Corrn. = Off
Tilt Angle = 0.0 °
Signal A = SE2
Signal B = SE2
Scan Rotation = 0.0 °
Mixing = Off
Mix Signal = 0.5000
Aperture No. = 1
FIB Blanked = Yes
FIB Probe = 30KV:500 pA
FIB Imaging = SEM

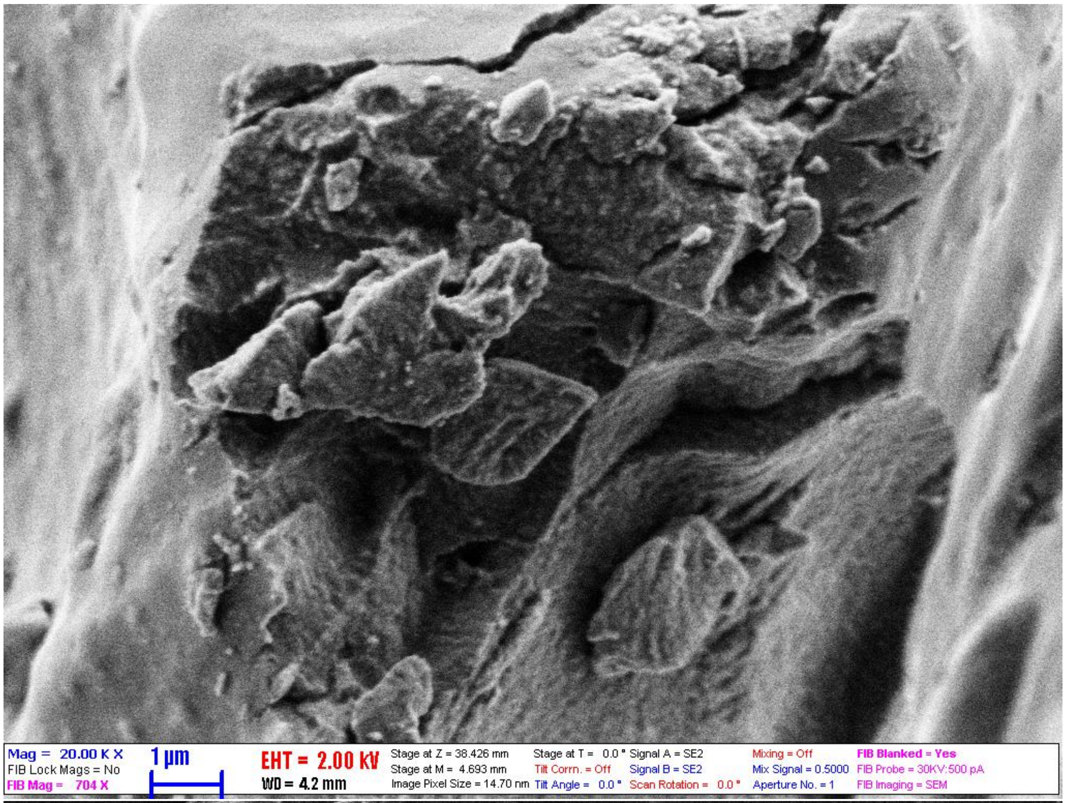
Mag = 20.00 K X
FIB Lock Mags = No
FIB Mag = 704 X
1 µm
EHT = 2.00 kV
WD = 4.2 mm
Stage at Z = 38.426 mm
Stage at M = 4.693 mm
Image Pixel Size = 14.70 nm
Stage at T = 0.0 °
Tilt Corrn. = Off
Tilt Angle = 0.0 °
Signal A = SE2
Signal B = SE2
Scan Rotation = 0.0 °
Mixing = Off
Mix Signal = 0.5000
Aperture No. = 1
FIB Blanked = Yes
FIB Probe = 30KV:500 pA
FIB Imaging = SEM

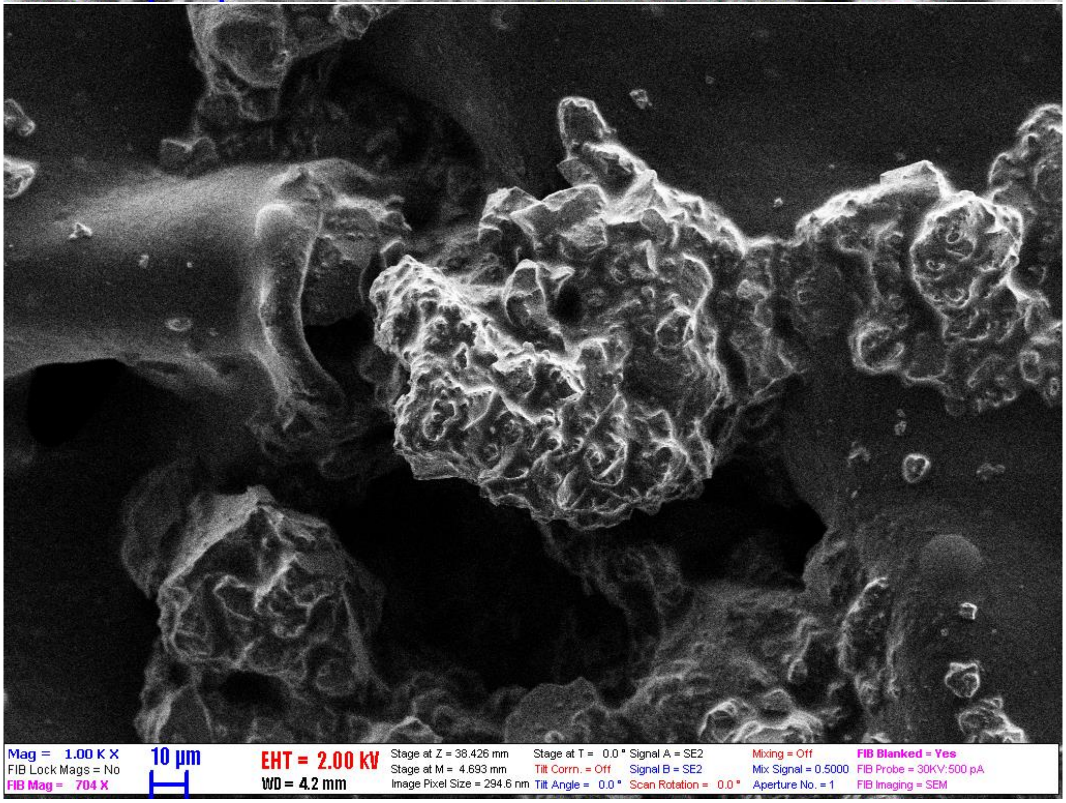
Mag = 1.00 K X
FIB Lock Mags = No
FIB Mag = 704 X
10 µm
EHT = 2.00 kV
WD = 4.2 mm
Stage at Z = 38.426 mm
Stage at M = 4.693 mm
Image Pixel Size = 294.6 nm
Stage at T = 0.0 °
Tilt Corrn. = Off
Tilt Angle = 0.0 °
Signal A = SE2
Signal B = SE2
Scan Rotation = 0.0 °
Mixing = Off
Mix Signal = 0.5000
Aperture No. = 1
FIB Blanked = Yes
FIB Probe = 30KV:500 pA
FIB Imaging = SEM

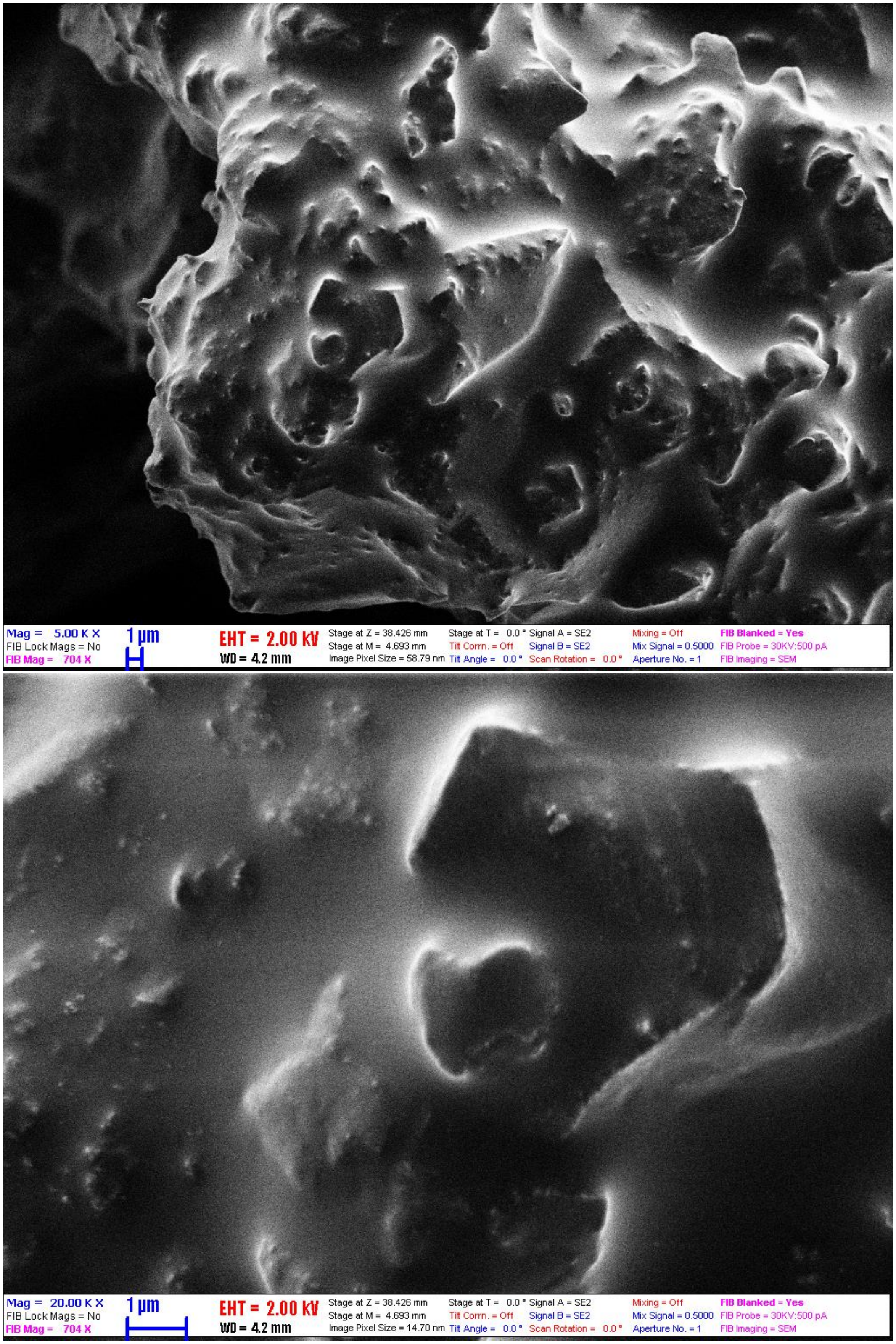

Figure S7 **IM-BF4-0.5**

2. EDS spectra of the IL – silica nanocomposites

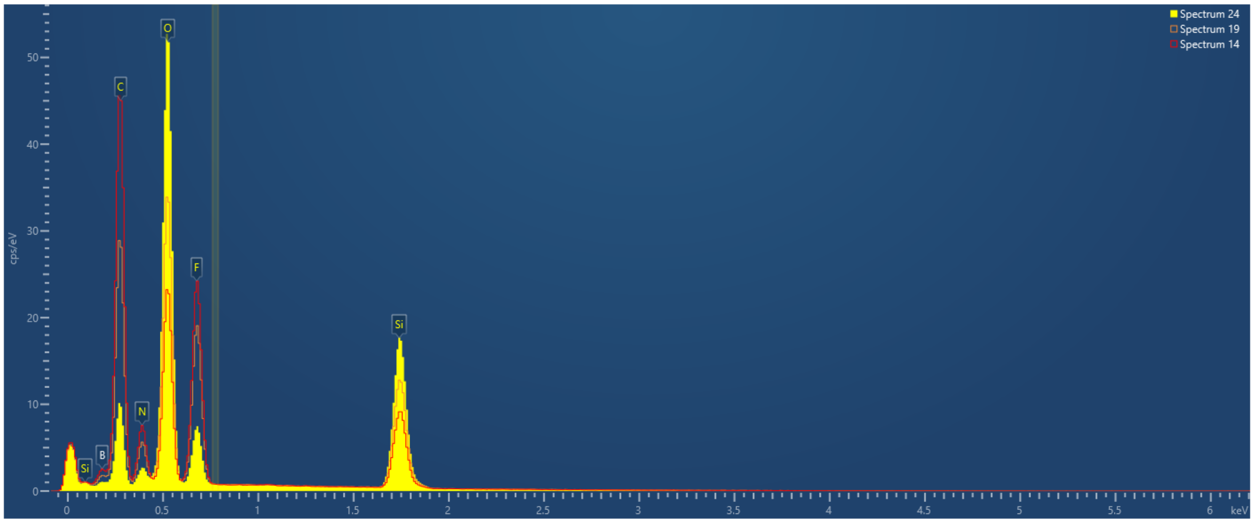

Figure S8 EDS spectra of samples **IM-BF4-0.1, IM-BF4-0.3, IM-BF4-0.5 .**

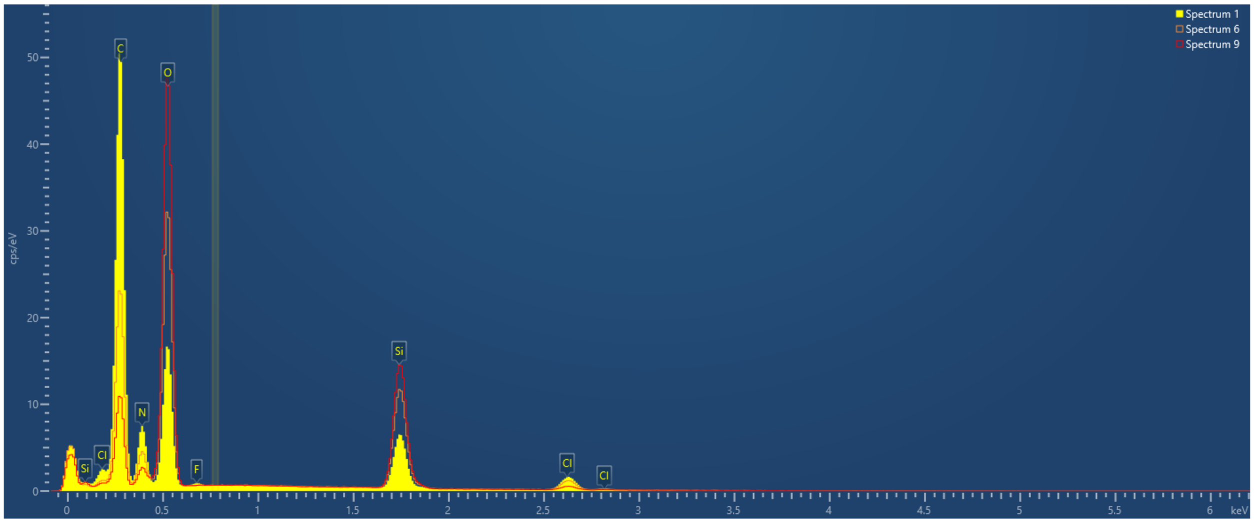

Figure S9 EDS spectra of samples **IM-Cl-0.1, IM-Cl-0.3, IM-Cl-0.5 .**
